# Genome-wide promoter methylation analysis in neuroblastoma identifies prognostic methylation biomarkers

**DOI:** 10.1186/gb-2012-13-10-r95

**Published:** 2012-10-03

**Authors:** Anneleen Decock, Maté Ongenaert, Jasmien Hoebeeck, Katleen De Preter, Gert Van Peer, Wim Van Criekinge, Ruth Ladenstein, Johannes H Schulte, Rosa Noguera, Raymond L Stallings, An Van Damme, Geneviève Laureys, Joëlle Vermeulen, Tom Van Maerken, Frank Speleman, Jo Vandesompele

**Affiliations:** 1 Center for Medical Genetics, Department of Pediatrics and Genetics, Ghent University, De Pintelaan 185, 9000 Ghent, Belgium; 2 Faculty of Education, Health and Social Work, University College Ghent, 9000 Ghent, Belgium; 3 Department of Mathematical Modelling, Statistics and Bio-informatics, Ghent University, Coupure Links 653, 9000 Ghent, Belgium; 4 MDxHealth, Tour 5 GIGA, Avenue de l'Hôpital 11, 4000 Liège, Belgium; 5 NXTGNT, Ghent University, De Pintelaan 185, 9000 Ghent, Belgium; 6 Children's Cancer Research Institute, St Anna Kinderkrebsforschug, Zimmermannplatz 10, A-1090 Vienna, Austria; 7 University Children's Hospital Essen, Hufelandstraße 55, 45122 Essen, Germany; 8 Department of Pathology, Medical School, University of Valencia, Blasco Ibañez 17, 46010 Valencia, Spain; 9 National Children's Research Centre, Our Lady's Children's Hospital, Crumlin, Dublin 12, Ireland; 10 Department of Molecular and Cellular Therapeutics, Royal College of Surgeons in Ireland, York House, York Street, Dublin 2, Ireland; 11 Department of Pediatrics, Brussels University Hospital, Laarbeeklaan 101, 1090 Brussels, Belgium; 12 Department of Pediatric Hematology and Oncology, Ghent University Hospital, De Pintelaan 185, 9000 Ghent, Belgium; 13 Pédiatrie, Hôpital de Jolimont, Rue Ferrer 159, 7100 La Louvière (Haine-Saint-Paul), Belgium; 14 Department of Clinical Chemistry, Microbiology and Immunology, Ghent University Hospital, De Pintelaan 185, 9000 Ghent, Belgium

## Abstract

**Background:**

Accurate outcome prediction in neuroblastoma, which is necessary to enable the optimal choice of risk-related therapy, remains a challenge. To improve neuroblastoma patient stratification, this study aimed to identify prognostic tumor DNA methylation biomarkers.

**Results:**

To identify genes silenced by promoter methylation, we first applied two independent genome-wide methylation screening methodologies to eight neuroblastoma cell lines. Specifically, we used re-expression profiling upon 5-aza-2'-deoxycytidine (DAC) treatment and massively parallel sequencing after capturing with a methyl-CpG-binding domain (MBD-seq). Putative methylation markers were selected from DAC-upregulated genes through a literature search and an upfront methylation-specific PCR on 20 primary neuroblastoma tumors, as well as through MBD- seq in combination with publicly available neuroblastoma tumor gene expression data. This yielded 43 candidate biomarkers that were subsequently tested by high-throughput methylation-specific PCR on an independent cohort of 89 primary neuroblastoma tumors that had been selected for risk classification and survival. Based on this analysis, methylation of *KRT19*, *FAS*, *PRPH*, *CNR1*, *QPCT*, *HIST1H3C*, *ACSS3 *and *GRB10 *was found to be associated with at least one of the classical risk factors, namely age, stage or *MYCN *status. Importantly, *HIST1H3C *and *GNAS *methylation was associated with overall and/or event-free survival.

**Conclusions:**

This study combines two genome-wide methylation discovery methodologies and is the most extensive validation study in neuroblastoma performed thus far. We identified several novel prognostic DNA methylation markers and provide a basis for the development of a DNA methylation-based prognostic classifier in neuroblastoma.

## Background

Neuroblastoma (NB) is a neuroectodermal tumor that originates from precursor cells of the sympathetic nervous system and represents the most common extra-cranial solid tumor of early childhood. NB displays a highly variable clinical course, ranging from spontaneous regression to life-threatening disease [1].

Despite advances in multimodal anti-cancer therapies, survival rates for children with aggressive NB remain disappointingly low. Survival rates vary widely, depending on clinical features, such as age at diagnosis and tumor stage, as well as biological characteristics of the tumor. Amongst the latter, *MYCN *amplification has been used for many years as a genetic marker for therapy stratification [1]. More recently, a subset of high-risk tumors with non-amplified *MYCN *and 11q deletions was identified, while absence of segmental aberrations upon genome-wide DNA copy number analysis was found to be associated with excellent survival [2,3]. In order to facilitate the comparison of risk-based clinical trials, a new consensus approach for pretreatment risk classification has been designed including genetic parameters [1,4]. Despite this progress, additional markers for therapeutic stratification are warranted in order to avoid under- or overtreatment and to improve selection of ultra-high-risk patients for new experimental therapies. Recently, prognostic mRNA and microRNA (miRNA) signatures were developed to accommodate this need [5-7]. Here, we propose that the use of DNA methylation markers is a new and promising method for prognostic classification.

DNA methylation is the addition of a methyl group to carbon 5 of the cytosine within the dinucleotide CpG. Dense clusters of CpG dinucleotides, termed CpG islands, are often present in gene promoters and methylation of those regions typically results in transcriptional silencing of the gene. As such, abnormal DNA methylation in cancer cells leads to aberrant expression patterns [8]. In NB, the most described epigenetic alterations are DNA methylation of *CASP8 *[9] and *RASSF1A *[10], both associated with risk factors, such as *MYCN *amplification (MNA), age at diagnosis and tumor stage [11-15]. Recently, a few genome-wide methylation screening methodologies have been applied in NB, including re-expression analysis after treatment with 5-aza-2'-deoxycytidine (DAC), DNA methylation promoter arrays after capturing with methylated DNA immunoprecipitation (MeDIP) and methylation microarrays. These studies indicate that aberrant DNA methylation makes an important contribution toward NB tumor biology by downregulating specific genes and show the potential of using DNA methylation in future patient therapy stratification protocols [16-18]. Furthermore, the power of DNA methylation as a non-invasive, sensitive and specific biomarker has been demonstrated by measuring DNA methylation of *RASSF1A *in serum of primary NB patients [15] (for a detailed review see [19]). In order to improve the outcome prediction of NB patients, this study aims at establishing robust DNA methylation biomarkers that can identify patients with unfavorable prognosis.

## Results

### Discovery and integrated analysis: genome-wide methylation screening for selection of candidate biomarkers

**Figure 1:**
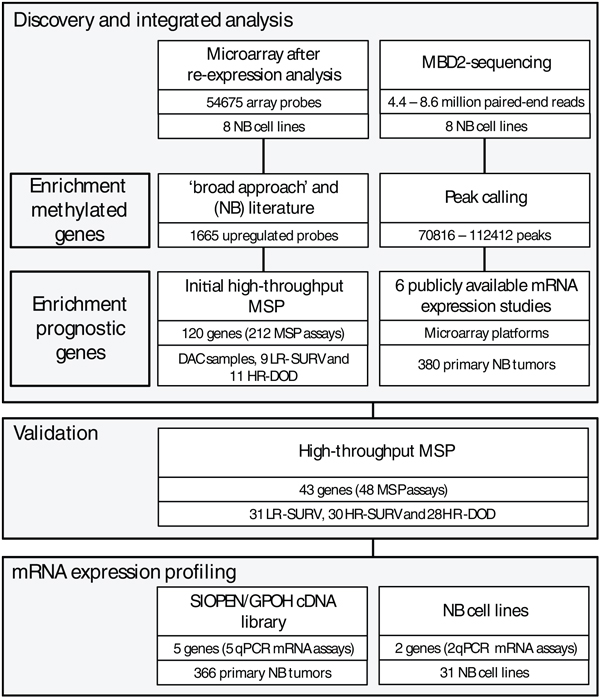
**Combining genome-wide methylation discovery and validation, several novel prognostic DNA methylation markers were identified in neuroblastoma (NB)**. Starting points are a microarray based re-expression study after treatment with 5-aza-2'-deoxycytidine (DAC) and a next-generation sequencing experiment using an enrichment strategy towards methylated DNA (methyl-CpG-binding domain (MBD) capture). Both were performed on the same panel of eight NB cell lines. Applying a bioinformatics and text-mining-based approach on the re-expression data, 120 candidate genes were selected and tested using an initial high-throughput methylation-specific PCR (MSP) screen. The MBD-seq data were combined with public mRNA expression studies to enrich for potential prognostic biomarkers. Using a rank-based scoring system, a final selection of 43 candidates was made, which were then tested using MSP on 89 primary NB samples (in the following subgroups: LR-SURV, low-risk patients with long follow-up; HR-DOD, high-risk patients that die of disease; HR-SURV, high-risk patients with long follow-up). Finally, mRNA expression levels of seven DNA methylation biomarkers were determined. qPCR, quantitative PCR.

The experimental setup of the study is summarized in Figure 1. In order to identify DNA methylation biomarkers in NB, we first applied two genome-wide methylation screening methodologies on eight NB cell lines: microarray after re-expression analysis and massively parallel sequencing after capturing with a methyl-CpG-binding domain (MBD- seq). The genome-wide assessment of gene expression reactivation upon DAC treatment is an indirect method to detect DNA methylation as the influence of the demethylating effect is measured at the transcriptional level using oligonucleotide chips. Out of 54,675 probes, a total of 3,624 were upregulated after DAC treatment compared to untreated controls (RankProd false discovery rate (FDR) <5%), of which 1,665 were upregulated at least two-fold in at least one cell line. Using a cutoff of at least a two-fold difference between the DAC-treated and the untreated sample, 989 probes were re-expressed in at least 2 cell lines. In order to select specific and sensitive methylation biomarkers from this high number of reactivated probes, an integrated bioinformatics approach was applied. The 1,665 upregulated probes identified by RankProd analysis were further filtered using a genome-wide promoter alignment strategy, referred to as the 'broad approach' in Hoque *et al*. [20]. This strategy consists of a genome-wide multiple alignment of promoter regions, where similar sequence regions thus cluster together and where the 'distance' (the number of nodes in the hierarchical alignment model) is shown to be able to predict novel biomarkers. Such approaches using DAC re-expression data have previously been successfully applied to enrich towards truly methylated genes [20,21]. We selected 150 genes that were either in the 'neighborhood' (less than 8 nodes away) of a known methylation marker or that clustered together in the promoter sequence alignment with a high number of reactivation events (at least two genes in the cluster showed at least three reactivation events). Integration with (NB) literature, using an in-house developed text-mining-based approach (using NCBI E-Utils to query PubMed, using all known gene aliases in combination with either DNA methylation-related or NB-related search terms), and selection for genes located in genomic regions reported as recurrently affected by DNA copy number changes in NB, eventually led to the selection of 120 candidate biomarkers, comprising 30 novel candidate markers and 90 known methylation markers in other tumor types. To obtain direct evidence for DNA methylation and to further select prognostic biomarkers, the selected 120 candidate biomarkers were tested on the DAC-treated and untreated NB cell lines CLB-GA, LAN-2, N206, SH-SY5Y and SJNB-1, and primary NB samples (9 low-risk survivors (LR-SURV) and 11 high-risk deceased (HR-DOD) patients; for details see Material and methods), using high-throughput methylation-specific PCR (MSP). In the NB cell lines, the DAC-treated samples show less methylation calls in comparison to untreated samples (130 MSP assays (64%) are more frequently methylated in the untreated samples), and taking all MSP assays into account the average number of methylated samples per assay is 0.39 for the DAC-treated cell lines versus 1.47 for the untreated cell lines (*P *= 0.0002), revealing dense methylation in genes upregulated upon DAC treatment and efficient demethylation by DAC (data not shown). The complete results of the initial high-throughput MSP screening on the primary NB samples can be found in Additional file 1.

The second genome-wide DNA methylation screening methodology we applied, to the same eight NB cell lines, was MBD-seq: massively parallel sequencing of methylation-enriched DNA fragments, whereby the enrichment is based on the capture of methylated sheared DNA using the high affinity of the methyl-CpG-binding domain () of the protein MBD2 towards methylated cytosines. Sequencing yielded 4.4 to 8.6 million paired-end reads, depending on the cell line, and after peak calling 70,816 to 112,412 peaks were detected, representing genomic regions methylated in the corresponding cell line. Between 7,612 and 11,178 of these peaks (around 10% of all identified peaks) are located in promoter regions of annotated genes (-1,500 bp to +1,000 bp around the transcriptional start site (TSS)). These 'methylation peaks' were visualized in the Integrative Genomic Viewer [22], showing that promoter regions that are well known to be heavily methylated in NB were confirmed - for example, the protocadherin β (*PCDHB*) family cluster (Additional file 2) [23,24]. In some regions (for example, in the promoter regions of *HIST1H3C *and *ACSS3*) it was also possible to distinguish different DNA methylation profiles between MNA (IMR-32, LAN-2 and N206) and *MYCN *single copy (SH-SY5Y, SK-N-AS, CLB-GA and SJNB-1) NB cell lines (Additional file 2). Using the R/BioC package DESeq [25], 510 regions were identified as differentially methylated between *MYCN *amplified and single copy cell lines, of which 95 are in close proximity to an annotated TSS (-1,500 bp to +1,000 bp). Also, some miRNAs appeared to be methylated in their promoter region.

After peak calling, we also performed gene set enrichment analysis [26], using a custom, ranked list of genes with at least one MBD peak present in a region -1,500 bp to +500 bp around its TSS, in order to explore whether promoter regions that are enriched after MBD capture are often re-expressed as well upon DAC treatment. This analysis clearly showed a high enrichment score for each cell line (enrichment scores from 0.32 to 0.36; FDR q-value <0.01), demonstrating that a large portion of methylated regions (captured by MBD) are indeed reactivated upon DAC treatment. The overlap between the two genome-wide datasets can be further explored by intersecting them. In total, 183 genes are both re-activated upon DAC treatment (at least 1 log2 difference after and before treatment) and have an MBD peak in their promoter regions (-1,500 bp to +1,000 bp around the TSS) in at least 2 of the 8 investigated NB cell lines. Of these 183 genes, 46 are both re-expressed and methylated in 3 cell lines, 9 in 4 cell lines and 5 in at least 5 cell lines.

As we feared that only using cell lines in the selection phase of potential prognostic DNA methylation biomarkers would lead to the identification of methylated markers not necessarily related with prognosis, six publicly available mRNA expression studies [27-34] were included in the analysis. In these studies, which comprise mRNA expression data of 380 primary NB tumors, identifying differentially expressed probes (genes) between prognostic groups would allow us to pinpoint potential prognostic methylated promoter regions in our methylome maps. Finally, a rank-based scoring system was used to prioritize genes that show methylation, re-expression after DAC treatment and differential expression (related with risk) across the prognostic groups. This score scheme uses the individual ranks of each analysis. In brief, DAC reactivation is ranked according to FDR rate (as determined by RankProd analysis), MDB-seq data are ranked according to peak *P*-values and expression data are ranked according to FDR (determined by RankProd analysis). Each data source is given the same weight and a combined rank is calculated (for details, see Materials and methods). This scoring system combined all generated data and allowed us to select 43 top-ranking and thus strong prognostic methylation candidate genes without the need to use rather artificial threshold values for the different datasets.

### Validation: determining the prognostic power of DNA-methylation biomarkers

For these 43 genes, 48 MSP assays were designed and tested on 3 NB cell lines (IMR-32, SK-N-AS and SH-SY5Y) and the HCT-116 DKO cell lines, along with an independent cohort of 89 primary NB samples. Within the 89 primary NB sample set all three prognostic groups (LR-SURV, HR-DOD and high-risk survivors (HR-SURV); for details see Material and methods) were approximately equally represented. The complete matrix with all MSP results of all samples and a global overview of the MSP results per assay can be found in Additional file 3. Over 60% of the designed assays indeed detected methylation for the respective marker in at least 10% of the selected NB tumors. Ten MSP assays (*COL6A3*, miR-1225, miR-3177, *PCDHA6*, *PLXNC1*, *ANKRD43*, *ADRB2*, *APOE*, miR-671 and *QPCT*) revealed methylation in at least 75% of the patient samples, and the MSP assays for *KCND2*, *PRPH*, *KRT19 *(assay 83159) and *TNFRSF10D *were methylated in 50% to 75% of the patient samples. We could also detect DNA methylation in the promoter region of miR-1225, miR-3177, miR-671 and miR-663, methylated in 99%, 99%, 79% and 4% of the patient samples, respectively.

Unique in this study is the use of three discrete prognostic patient groups, which allowed us to assess differential methylation across all these prognostic groups. Therefore, we performed hierarchical cluster analysis on the methylation data of all 48 MSP assays on the entire NB tumor cohort, revealing two clusters with a separation between high-risk (HR) and low-risk (LR) patients (heatmap in Additional file 3). Furthermore, the overall methylation pattern in the primary NB tumor samples was compared by calculating the number of methylation events for each sample. This indicates that HR patients show, on average, more methylation events compared to LR patients (*P *< 0.001; HR-DOD, 17.21 methylation events (95% confidence interval (CI) 15.62 to 18.81); HR-SURV, 17.13 methylation events (95% CI 15.81 to 18.46); LR-SURV, 13.00 methylation events (95% CI 11.86 to 14.14)). Also on the individual marker level, some MSP assays are differentially methylated across the prognostic patient groups: *KRT19 *and *ACSS3*. These genes are more frequently methylated in HR patients compared to LR patients (Table 1). Within the HR group, *HIST1H3C *shows a tendency to be more frequently methylated in HR-DOD compared to HR-SURV samples (21% in HR-DOD versus 7% in HR-SURV), while *KRT19 *(32% versus 48%) and *ACSS3 *(25% versus 47%) show the inverse pattern.

**Table 1:** Several individual markers are differentially methylated between the prognostic groups and neuroblastoma risk factors

Type	Subtype	*KRT19*	*FAS*	*PRPH*	*CNR1*	*QPCT*	*HIST1H3C*	*ACSS3*	*GRB10*
Prognostic group	LR-SURV	0/31 (0%)	1/31 (3%)	14/31 (45%)	2/31 (6%)	18/31 (58%)	0/31 (0%)	0/31 (0%)	6/31 (19%)
	HR-SURV	14/30 (48%)	8/30 (27%)	24/30 (80%)	10/30 (33%)	25/30 (83%)	2/30 (7%)	14/30 (47%)	13/30 (43%)
	HR-DOD	9/28 (32%)	6/28 (21%)	19/28 (68%)	10/28 (36%)	24/28 (86%)	6/28 (21%)	7/28 (25%)	11/28 (39%)
Stage	Stage 1	0/21 (0%)	0/21 (0%)	8/21 (38%)	1/21 (5%)	13/21 (62%)	0/21 (0%)	0/21 (0%)	4/21 (19%)
	Stage 2	1/12 (8%)	2/12 (17%)	8/12 (67%)	2/12 (17%)	8/12 (67%)	0/12 (0%)	1/12 (8%)	3/12 (25%)
	Stage 3	9/17 (53%)	4/17 (24%)	13/17 (77%)	9/17 (53%)	15/17 (88%)	3/17 (18%)	9/17 (58%)	8/17 (47%)
	Stage 4	13/39 (33%)	9/39 (15%)	28/39 (72%)	10/39 (26%)	31/39 (80%)	5/39 (18%)	11/39 (28%)	15/39 (39%)
*MYCN*	*MYCN *single copy	7/50 (14%)	2/50 (4%)	24/50 (48%)	5/50 (10%)	31/50 (62%)	0/50 (0%)	2/50 (4%)	14/50 (28%)
	*MYCN *amplified	16/39 (41%)	13/39 (33%)	33/39 (85%)	17/39 (44%)	36/39 (92%)	8/39 (21%)	19/39 (49%)	16/39 (41%)
Age	Age at diagnosis > 12 months	21/53 (40%)	14/53 (26%)	37/53 (69%)	18/53 (34%)	46/53 (87%)	8/53 (15%)	21/53 (40%)	24/53 (45%)
	Age at diagnosis < 12 months	2/36 (6%)	1/36 (3%)	20/396(56%)	4/36 (11%)	21/36 (58%)	0/36 (0%)	0/36 (0%)	6/36 (17%)
	Age at diagnosis > 18 months	20/45 (44%)	13/45 (29%)	33/45 (73%)	17/45 (38%)	40/45 (89%)	8/45 (18%)	19/45 (49%)	23/45 (51%)
	Age at diagnosis < 18 months	3/44 (7%)	2/44 (5%)	24/44 (55%)	5/44 (11%)	27/44 (61%)	0/44 (0%)	2/44 (5%)	7/44 (16%)
Overall total		23/89 (26%)	15/89 (17%)	57/89 (64%)	22/89 (25%)	67/89 (75%)	8/89 (9%)	21/89 (24%)	30/89 (34%)
									
Statistics (Fisher's exact *P*-value)									
Prognostic group		**<0.001**	0.151	0.112	0.068	0.165	0.0624	**<0.001**	0.405
*MYCN*		0.0594	**0.008**	**0.008**	**0.008**	**0.017**	**0.0146**	**<0.001**	0.708
Stage		**0.007**	0.287	0.221	0.059	0.683	0.448	**0.008**	0.700
Age cutoff 12 months		**0.008**	**0.045**	0.579	0.138	0.059	0.123	**<0.001**	0.059
Age cutoff 18 months		**0.002**	**0.045**	0.326	0.059	**0.045**	0.0594	**0.0015**	**0.012**

The number (percentage) of methylated samples in each stratum is given. *P*-values according to the Fisher's exact test, corrected for multiple testing (Benjamini-Hochberg). HR-DOD, high-risk deceased patients; HR-SURV, high-risk patients alive for at least 1,000 days follow-up; LR-SURV, low-risk patients alive for at least 1,000 days follow-up. *P*-values in bold indicate significant associations.

Some individual MSP assays were also associated with one or more NB risk factors (stage, *MYCN *status and age at diagnosis), and are thus potential prognostic biomarkers in NB (Table 1). In this analysis, the age at diagnosis was tested using two different age cutoffs. The 12 months cutoff was chosen as it was used for therapy stratification and as a criterion in the sample selection. The more recently established cutoff of 18 months [1,35,36] was also taken into account. Newly discovered methylated markers are *FAS*, *PRPH*, *CNR1*, *QPCT*, *HIST1H3C*, *ACSS3 *and *GRB10*, methylation of which is associated with at least one of the NB risk factors. Table 1 further indicates that the difference in the methylation status of *HIST1H3C *and *ACSS3 *between *MYCN *single copy and MNA NB cell lines as detected by MBD-seq is reflected in the MSP results of the primary tumors as well, as *HIST1H3C *and *ACSS3 *are almost exclusively methylated in MNA samples.

**Figure 2:**
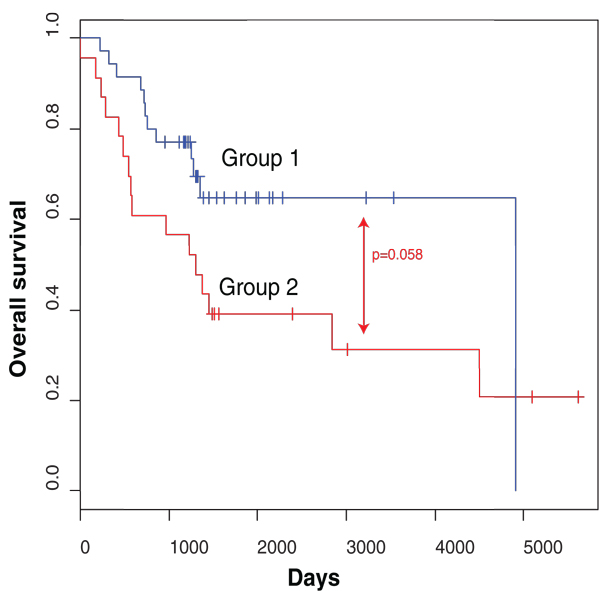
**The combined methylation status of *CNR1*, *ACSS3*, *HIST1H3C *and *PRPH *can potentially discriminate HR patients**. The Kaplan-Meier plot shows overall survival in the high-risk samples of the high-throughput MSP screening according to their predicted overall survival status based on leave-one-out decision tree analysis using the methylation data of *CNR1*, *ACSS3*, *HIST1H3C *and *PRPH*. Group 1 is predicted to survive, group 2 to die of disease. The *P*-value is determined using log-rank test (Mantel-Cox). Time is indicated in days, starting from diagnosis.

Survival analysis using the complete MSP data set indicates that patients with less methylation events showed better survival rates than patients with a high number of methylation events (*P *= 0.01; Additional file 3), as this analysis principally discriminates HR and LR patients. In order to assess to what extent our MSP data set is able to predict overall survival (OS) in HR-SURV versus HR-DOD patients, leave-one-out decision tree analysis was performed and repeated 58 times (the number of HR patients). For this analysis, we only included the data from MSP assays (un)methylated in at least three samples. Comparison of the 58 generated decision trees showed that 4 DNA methylation biomarkers (*CNR1*, *ACSS3*, *HIST1H3C *and *PRPH*) are included in at least 50% of the resulting classifiers. Then, leave-one-out decision tree analysis was redone, but this time using only the methylation data of *CNR1*, *ACSS3*, *HIST1H3C *and *PRPH*. Afterwards, the predictions for all 58 HR samples were visualized in a Kaplan-Meier plot (Figure 2). This analysis indicates that the combined methylation status of *CNR1*, *ACSS3*, *HIST1H3C *and *PRPH *has the potential to discriminate between HR-SURV and HR-DOD patients (*P *= 0.058).

**Figure 3:**
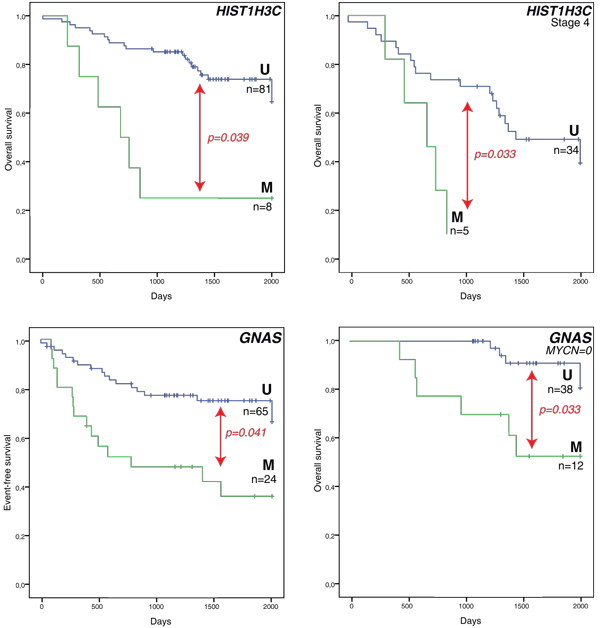
**Methylation of *HIST1H3C *and *GNAS *is associated with worse survival outcome**. Kaplan-Meier plots on the left show overall survival or event-free survival for all 89 primary neuroblastoma samples, those on the right overall survival in a specific stratum based on one of the risk factors only. Survival curves indicated with 'M' are the methylated samples, survival curves associated with the unmethylated assay are indicated with 'U'. The numbers of patients are indicated (n) and *P*-values are determined using a log-rank test (Mantel-Cox; multiple testing correction by Benjamini-Hochberg). Time is indicated in days, starting from diagnosis and censored to 2,000 days (censored samples are indicated with vertical lines crossing the overall survival curves). *MYCN *= 0: *MYCN *single copy.

Survival analysis was also performed on the individual marker level. We first tested differences between the HR-DOD and LR-SURV groups using the univariate log-rank test (with multiple testing correction). This first analysis indicates that six genes (*KRT19*, *FAS*, *CNR1*, *HIST1H3C*, *ACSS3 *and *GNAS*) are significantly related to survival when comparing these patient groups. As we also want to discriminate the HR patient groups (HR-DOD and HR-SURV), we then used the entire dataset (all samples) to assess which of these six genes were associated with survival (in a specific stratum only, such as only in *MYCN *single copy samples). These results are shown in Table 2. According to log-rank tests, *HIST1H3C *methylation is associated with both OS and event-free survival (EFS), while *GNAS *methylation is associated with EFS. As NB is a heterogeneous disease, these biomarkers may be suited to a specific subgroup of patients for predicting survival. For example, *HIST1H3C *methylation only occurs in high-stage tumors with MNA (6/17 (35%) in HR-DOD patients versus 2/22 (9%) in HR-SURV patients). Figure 3 shows the Kaplan-Meier plots for *HIST1H3C *and *GNAS *methylation (OS or EFS and OS in specific strata related to one of the risk factors).

**Table 2:** Several individual DNA methylation markers are associated with survival

Statistics	*KRT19*	*FAS*	*CNR1*	*HIST1H3C*	*ACSS3*	*GNAS*
HR-DOD versus LR-SURV (*P*-value OS)	**0.037**	**0.028**	**0.043**	**0.002**	**0.002**	**0.012**
HR-DOD versus LR-SURV (*P*-value EFS)	**0.039**	**0.049**	**0.039**	**0.039**	0.079	**0.039**
HR-DOD versus LR-SURV and HR-SURV (*P*-value OS)	0.687	0.639	0.423	**0.039**	0.691	0.221
HR-DOD versus LR-SURV and HR-SURV (*P*-value EFS)	0.665	0.467	0.414	**0.041**	0.939	**0.041**
HR-DOD versus LR-SURV and HR-SURV (*P*-value OS (stratum))			**Age < 12 months 0.035**	**Stage 4 0.033**		***MYCN *= 0 0.033**
HR-DOD versus LR-SURV and HR-SURV (*P*-value EFS (stratum))		**Age < 12 months 0.014**				***MYCN *= 0 0.001**

Log-rank test statistics (Mantel-Cox) are given (multiple testing correction by Benjamini-Hochberg) for comparison between the ultra-high risk group (HR-DOD) versus the low risk group (LR-SURV), and between the ultra-high risk group (HR-DOD) and all survivors (LR-SURV and HR-SURV). If a significant association (*P *< 0.05) was found in a particular stratum (associated with risk factors), this stratum is shown (multiple testing correction for the different comparisons by Benjamini-Hochberg). *MYCN *= 0: *MYCN *single copy ; EFS, event-free survival; OS, overall survival. *P*-values in bold indicate significant associations.

### mRNA expression profiling: determining transcriptional silencing of DNA methylation biomarkers

As it is known that promoter methylation may cause transcriptional silencing of the gene, we further measured the mRNA expression levels of five promising DNA methylation biomarkers that were methylated in a substantial fraction of HR patients (*CNR1*, *GRB10*, *KRT19*, *PRPH *and *QPCT*). Quantitative RT-PCR assays were developed and tested on 366 primary NB tumor samples. Table 3 displays the results of the comparisons of the expression levels of each DNA methylation biomarker between the different NB tumor stages, *MYCN *single copy and MNA tumors, the two age groups (using both the 12 and 18 months cutoff), and surviving and deceased patients. As an example, the mRNA expression levels of these genes across the NB tumor stages are depicted in Additional file 4. Out of the 366 primary NB tumors, 245 could be assigned to one of the prognostic groups defined in this study (Additional file 5), which allowed us to asses differential mRNA expression between these groups as well. For all genes mRNA expression levels were significantly higher in the LR group compared to the HR groups. As methylation of these genes was mainly detected in the HR groups, this suggests that methylation may contribute to the transcriptional silencing of these genes.

**Table 3:** mRNA expression level of several markers associates with neuroblastoma risk factors, prognostic groups and survival

Grouping variable	Statistics	*CNR1*	*GRB10*	*KRT19*	*PRPH*	*QPCT*
Stage	Kruskal-Wallis *P*-value	**<0.001**	**0.008**	0.118	**0.010**	**<0.001**
*MYCN*	Mann-Whitney *P*-value	**<0.001**	**<0.001**	**<0.001**	**<0.001**	**<0.001**
Age cutoff 12 months	Mann-Whitney *P*-value	**<0.001**	0.609	**<0.001**	**0.005**	**<0.001**
Age cutoff 18 months	Mann-Whitney *P*-value	**<0.001**	0.810	**<0.001**	**0.003**	**0.006**
Overall survival status	Mann-Whitney *P*-value	**<0.001**	**0.003**	**0.023**	**<0.001**	**<0.001**
Prognostic group	Kruskal-Wallis *P*-value	**<0.001**	**0.002**	**0.005**	**<0.001**	**<0.001**

The statistical test used is shown and *P*-values (corrected for multiple testing using Benjamini-Hochberg) are indicated. *P*-values in bold indicate significant associations.

Survival analysis using Cox proportional hazards further shows that low mRNA expression levels of *CNR1 *(hazard ratio (HR) 0.768; 95% CI 0.619 to 0.953; *P *= 0.028), *GRB10 *(HR 0.613; 95% CI 0.433 to 0.866; *P *= 0.015) and *PRPH *(HR 0.714; 95% CI 0.566 to 0.922; *P *= 0.015) were significantly associated with poor survival. After dichotomization of the mRNA expression data, using the median relative mRNA expression value as a cutoff, Kaplan-Meier survival curves were plotted (log-rank test; Additional file 5).

**Figure 4:**
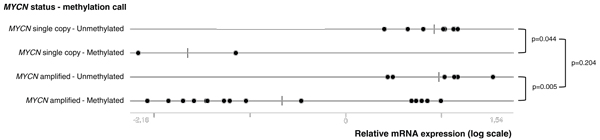
***HIST1H3C *has lower mRNA expression levels in NB cell lines in which the *HIST1H3C *promoter is methylated**. Thirty-one NB cell lines were categorized according to their *MYCN *amplification and *HIST1H3C *methylation status. The relative *HIST1H3C *mRNA expression level of each of these cell lines is indicated (*MYCN *single copy - Unmethylated, *MYCN *single copy - Methylated; *MYCN *amplified - Unmethylated, *MYCN *amplified - Methylated). *P*-values according to the Mann-Whitney test are also indicated.

An interesting observation in our MBD-seq and MSP data is the fact that *HIST1H3C *and *ACSS3 *are differentially methylated between *MYCN *single copy and MNA NB cell lines and primary tumors (Table 1; Additional file 2). To further explore this finding, the *HIST1H3C *and *ACSS3 *MSP assays were tested on 31 NB cell lines, of which 10 were *MYCN *single copy and 21 MNA (Additional file 4). In addition, we also profiled *HIST1H3C *and *ACSS3 *mRNA expression levels in these cell lines, in order to assess the direct relationship between promoter methylation and mRNA expression and to compare this relationship between *MYCN *single copy and MNA cell lines. The significant differential methylation status of *HIST1H3C *and *ACSS3 *between *MYCN *single copy and MNA samples was confirmed in the NB cell lines (*HIST1H3C*, methylated in 15/21 (71%) MNA cell lines and in 2/10 (20%) *MYCN *single copy cell lines, *P *= 0.018; *ACSS3*, methylated in 20/21 (95%) MNA cell lines and in 3/10 (30%) *MYCN *single copy cell lines, *P *< 0.001). Moreover, expression of *HIST1H3C *mRNA was significantly lower in methylated samples compared to unmethylated samples, both in MNA (*P *= 0.005) and *MYCN *single copy (*P *= 0.044) cell lines (Figure 4). These data support the idea that *HIST1H3C *promoter methylation contributes to transcriptional silencing of the gene. Figure 4 further indicates that the *MYCN *status itself is not significantly associated with *HIST1H3C *mRNA expression levels (*P *= 0.204). As *ACSS3 *is expressed at very low mRNA levels, we could not correlate its mRNA expression data with the methylation data (data not shown).

## Discussion

Thus far, most of the studies analyzing DNA methylation patterns in NB have been candidate gene-based, with the methylation status of the promoter region for only a limited number of genes being tested. These candidate genes were selected based either on prior knowledge of NB tumor biology or on the fact of being methylated in other tumor types. As a consequence, only few DNA methylation biomarkers, such as *KRT19*, *TNFRSF10D*, *CASP8*, *ZMYND10 *and *RASSF1A*, were previously related with NB risk factors or survival [11,13-15,18,37-41]. In order to identify new DNA methylation biomarkers in NB, we applied a multilevel experimental approach. In the discovery phase we established a genome-wide methylome map of eight NB cell lines. These cell lines were profiled using gene expression microarrays before and after DAC treatment, and using MBD capture followed by next-generation sequencing (NGS). The combination of both methodologies enabled the identification of regions that are both methylated and undergo re-expression upon DAC treatment. So far, only MeDIP chips were used in whole promoter profiling studies on NB [9], making this study the first one using NGS for unbiased and more sensitive assessment of genome-wide DNA methylation patterns in NB. Our results emphasize the potential of this epigenetic sequencing technique, as it enables the investigation of the methylome or epigenome of a sample in great detail at a feasible cost.

Integration of these methylome maps with genome-wide gene expression profiles led to a selection of 43 candidate biomarkers that were tested on 89 primary NB patient samples. All samples were assigned to one of three discrete prognostic patient groups (low-risk survivors (LR-SURV), high-risk deceased (HR-DOD) and high-risk survivors (HR-SURV)). While most NB methylation studies did not discriminate between HR-SURV and HR-DOD patients, we believe this is an important clinical question, as both prognostic groups are currently considered high-risk and uniformly treated, making the present study unique in its concept. As we make use of amplified bisulfite-converted DNA, only limited amounts (100 to 200 ng) of tumor DNA are required to test over 100 MSP assays. The MBD-seq results greatly help in designing the assays in the most informative regions, which is important as the assay location is critically important, again confirmed in this study for a number of genes for which multiple assays were designed (for example, *TGFBI *and *KRT19*). The combination of the number of samples and assays used in this study further makes it the most comprehensive methylation study in NB. Furthermore, the high-throughput validation pipeline allows fast and accurate follow-up validation of potential candidate DNA methylation biomarkers for large numbers of patients. Indeed, PCR-based detection methodologies are robust and can thus be used in a wide range of laboratory settings for a low price without the need of special equipment other than for qPCR and (microfluidic) electrophoresis, both present in most molecular laboratories. The presented DNA methylation screening and validation methodology can thus easily be adapted by (cancer) researchers addressing similar questions in other research fields.

In this study, several novel biomarkers were established in addition to known DNA methylation biomarkers in NB, such as *KRT19*, *TGFBI*, *TNFRSF10D *and *TNFRSF10A *[14,18,37,42,43]. Interestingly, some of these novel genes were previously reported to be important in NB biology (without reference to their epigenetically altered status) or were described as epigenetic biomarkers in other tumor entities, such as *FAS*, which encodes a member of the tumor necrosis factor receptor (TNFR) superfamily [44-50]. Several other novel methylation biomarkers were also shown to be differentially methylated between HR and LR patients, and many of these were associated with NB risk factors or with survival. However, discriminating HR-DOD and HR-SURV patients is challenging. While only a few individual MSP designs (*HIST1H3C*, *KRT19 *and *ACSS3) *were moderately discriminatory between these two HR subgroups, the combined methylation data analysis of *CNR1*, *ACSS3*, *HIST1H3C *and *PRPH *indicates the potential of DNA methylation biomarkers in stratifying HR NB patients. In this study, the difficulty of identifying individual biomarkers that differentiate between HR-DOD and HR-SURV patients may be explained by the fact that NB cell lines were used in the discovery phase, thus enriching for genes discriminating between HR and LR patients as NB cell lines can be considered models for aggressive HR tumors. To accommodate this, we plan to perform a large-scale discovery using MBD capture followed by NGS on primary NB tumors equally distributed over the three prognostic groups used here.

*PRPH *is one of the novel biomarkers identified and is differentially methylated across the prognostic patient groups. This gene encodes the cytoskeletal protein peripherin found in neurons of the peripheral nervous system, and is probably associated with maturation of the neuronal phenotype and hence serves as a differentiation marker for tumors derived from the neural crest [51]. In our study, *PRPH *methylation was mainly detected in more advanced tumor stages. Since promoter methylation may cause transcriptional silencing of the gene and advanced NB tumor stages are less differentiated [52], this is in line with the idea that high levels of peripherin contribute to more differentiated tumor stages. As demonstrated in this study, this idea is further strengthened by the fact that *PRPH *mRNA expression levels gradually decreased with increasing aggressiveness of the tumor. As whole genome sequence analysis recently showed that genes involved in neuritogenesis are recurrently affected in high-stage NB [53], the identification of *PRPH *methylation opens new research perspectives regarding NB therapy.

Next to protein-coding genes, some MSP assays were designed in the promoter region of miRNAs. Aberrant miRNA expression contributes majorly to NB tumor biology and has been extensively studied during the past few years. Most of these studies used miRNA microarrays or high-throughput RT-qPCR to analyze the miRNA expression patterns in primary NB tumor samples [54-56]. Although a broad deregulation of the miRNA expression profile in NB has been described, miRNA promoter hypermethylation is relatively unexplored. Up until now, the only miRNA for which the promoter region is known to be methylated in NB is miR-200b [57]. Interestingly, miR-1225, miR-3177 and miR-671 were found to be methylated in their promoter region in more than 75% of the NB tumors in our study. Currently, little is known about the putative function of these miRNAs, as they are not well described or not described at all in the literature [58,59].

Another interesting finding is that *MYCN *single copy and MNA samples show differential promoter methylation of *HIST1H3C *and *ACSS3*. Currently, little is known about the association between *MYCN *and DNA methylation of certain genes in NB, nor about the underlying molecular mechanisms. Previously, Teitz *et al*. [9,60] showed that DNA methylation of *CASP8 *is almost exclusively associated with MNA in both NB cell lines and primary tumors. They further noticed that *CASP8 *was hemi-methylated (only one allele) in stage 1, 2 and 3 NB, which may indicate that complete methylation of *CASP8 *may be coupled to another event, such as amplification of the *MYCN *gene. While this suggests that MNA is functionally linked to complete methylation of both *CASP8 *alleles, it is not clear if these two events occur concurrently, or if one event leads to the other. Obviously, genes differentially methylated between MNA and *MYCN *single copy samples need to be further functionally characterized, as this may lead to new insights into NB biology.

## Conclusions

Although international collaboration in the field of NB has invested tremendous effort in optimizing patient stratification and therapy protocols, OS rates remain low. This study shows that DNA methylation biomarkers have the potential to refine current risk assessment schemes. In contrast to most NB methylation studies that are candidate gene-based, we applied two genome-wide detection methodologies to discover hypermethylated regions in NB: re-expression analysis after demethylating DAC treatment and NGS after MBD capture. Furthermore, we present a high-throughput and semi-automated MSP pipeline, which was used to test the candidate DNA methylation markers on a large patient tumor cohort. We have identified novel aberrant promoter hypermethylation of protein coding genes and miRNAs in NB. Some of these DNA methylation biomarkers are associated with NB risk factors and/or survival, emphasizing the prognostic value of these markers and their potential to be used in a DNA methylation-based prognostic classifier in NB. The use of such a DNA methylation signature, discriminating HR patients, is demonstrated here by the combined methylation data analysis of *CNR1*, *ACSS3*, *HIST1H3C *and *PRPH*. Furthermore, some DNA methylation biomarkers showed low levels of mRNA expression in patient groups with high methylation levels. This suggests that promoter methylation may contribute to transcriptional silencing of these genes, which may be important in the pathogenesis of NB. Encouraged by these results, we will now extensively further validate these DNA methylation biomarkers and refine the methylome map of different prognostic NB patient groups.

## Materials and methods

### Neuroblastoma cell lines and primary tumors

In total, 33 well-characterized NB cell lines, authenticated using array comparative genomic hybridization and short tandem repeat genotyping, were included in this study (Additional files 2 and 4). DNA was isolated using the QIAamp DNA Mini Kit (Qiagen, Venlo, The Netherlands). In addition, 109 primary tumor samples of NB patients were collected prior to therapy at the Ghent University Hospital (Ghent, Belgium), the University Children's Hospital Essen (Essen, Germany), Our Lady's Children's Hospital Dublin (Dublin, Ireland) or the Hospital Clínico Universitario (Valencia, Spain). Informed consent was obtained from each patient's guardian and the study was approved by the ethical committee of the Ghent University Hospital (approval number B67020109912). Clinical characteristics of the patients are shown in Additional files 1 and 3. All NB patient samples were assigned to one of three defined risk groups based on risk parameters (tumor stage, *MYCN *status and age at diagnosis) and disease outcome. First, HR patients that died of disease (HR-DOD) as defined by stage 2/3, MNA, DOD; stage 4, age at diagnosis <12 months, MNA, DOD; or stage 4, age at diagnosis >12 months, DOD (n = 39). Second, HR patients alive (HR-SURV) after follow-up time >1,000 days (n = 30). Third, LR patients alive (LR-SURV) defined by stage 1/2, *MYCN *single copy, follow-up time >1,000 days; stage 3, *MYCN *single copy, age <12 months, follow-up time >1,000 days (status at last known follow-up is alive; n = 40). The clinical data of the 366 primary NB tumors (SIOPEN/GPOH cDNA library [6]), used to test the mRNA expression levels of the most promising DNA methylation biomarkers, can be found in Additional file 5.

### Microarray after re-expression analysis

Eight NB cell lines (CHP-902R, CLB-GA, IMR-32, LAN-2, N206, SH-SY5Y, SK-N-AS and SJNB-1) were grown in the presence of 3 µM DAC (Sigma, Bornem, Belgium)) for 3 days, as previously described, and untreated controls were also prepared [61]. After harvesting, RNA was extracted with the RNeasy Mini kit (Qiagen), accompanied by RNase free DNase treatment on column (Qiagen). After RNA quality check on the Experion (Bio-Rad, Nazareth, Belgium), sample preparation, hybridization to Affymetrix Human Genome U133 Plus 2.0 oligonucleotide chips and scanning were carried out according to the manufacturer's protocol at the VIB MicroArray Facility. Standard quality metrics (simpleaffy BioC package [62] - boxplots, visual inspection of the slides, 5'-3' degradation plot) demonstrated that the oligonucleotide chip data were of good quality. The BioC affy package was used to normalize (gc-RMA normalization) the expression levels and to obtain present/absent (expression/no expression) MAS 5.0 calls for each probe set. For all cell lines and for each probe set, the number of reactivation events was counted (absent in untreated cells and present in treated cells). Expression data (before and after DAC treatment) have been deposited into the Gene Expression Omnibus [GEO: GSE31229], according to the MIAME guidelines.

### MBD-seq

DNA samples (1 µg DNA) of the eight NB cell lines were sheared (Covaris S2) to an average length of 200 bp. Fragment distribution was determined by the Agilent 2100 Bioanalyzer and the concentration was determined using the Quant-iT PicoGreen dsDNA HS Assay Kit (Invitrogen, Ghent, Belgium). Starting from 200 ng sheared DNA, the MethylCollector Kit (ActiveMotif, La Hulpe, Belgium) was used to enrich for methylated fragments. Library preparation for multiplex Illumina sequencing was done by combining the DNA Sample Prep Master Mix Set 1 (New England Biolabs, Frankfurt am Main, Germany) and the Multiplexing Sample Preparation Oligo Kit (Illumina). Size selection of the library was done on a 2% agarose gel. Fragments of around 300 bp (±50 bp) were excised and purified. Illumina library amplification (21 cycles) was performed and concentration was determined. Paired-end sequencing was used for high confidence mapping of captured fragments (2 × 45 bp sequencing - Illumina GAIIx, NXTGNT). Paired-end reads were mapped on the human reference genome (GRCh37) using Bowtie 0.12.7 and peaks were called using MACS 1.4beta. For differential methylation analysis, PCR duplicates were removed and sequence tags counted by using the BioC packages ShortRead and rtracklayer [63,64]. Sequence tag counts per sample were used to compose a count matrix that could be processed by the BioC package DESeq [25]. Sequencing data (raw sequence files, WIG files for visualization of the mapping results and the BED peak files as determined by MACS) have been deposited into GEO [GEO:GSE31353].

### Selection of candidate biomarkers

#### Initial high-throughput MSP

In total, 212 MSP assays (Additional file 1) were designed in the promoter region of 120 corresponding genes re-expressed after DAC treatment, and tested on both the DAC-treated and untreated NB cell lines, 9 LR-SURV patients and 11 HR-DOD patients (Additional file 1). A total of 500 to 1,000 ng DNA of these samples was bisulfite-treated (EZ DNA Methylation Kit, Zymo Research, Irvine, CA, USA), eluted in 30 µl elution buffer and then tested on the BioTrove OpenArray (Life Technologies, Ghent, Belgium). Beta actin (*ACTB*) was used as a control and to normalize samples. The *in vitro *methylated HCT-116 DKO cell line (treated with *SssI*, Zymo Research) was used as a positive control. The methylation status for each MSP assay was determined, and called methylated if the melting temperature (Tm) of the amplicon was within a specific interval as defined by the positive control sample. These methylation calls were further analyzed by determining specificity and sensitivity of the HR-DOD samples versus LR-SURV samples.

#### Publicly available mRNA expression studies

Six publicly available mRNA expression studies [27-34] [GEO:GSE19274, GEO:GSE16237, GEO:GSE14880, GEO:GSE12460, GEO:GSE13136, GEO:GSE3960] were analyzed using RankProd analysis (BioC package [18]), to identify differentially expressed probes between prognostic groups (high-risk versus low-risk, high-stage versus low-stage, and MNA versus *MYCN *single copy).

#### Scoring system

Each analysis score of a promoter region (for example, RankProd FDR value and *P*-value for differential expression between risk groups, and *P*-values of the peak after MBD-seq) was ranked and given a score, ranging from tan(1) to 0 according to their rank. These individual scores were then summed and 43 top-ranking genes were selected for further analysis.

### High-throughput MSP

MSP assays were designed to only amplify the bisulfite-converted target region of interest and do not anneal to genomic DNA. As each primer contains at least two CpG sites, this means that a PCR product will only be generated if the template is methylated. We choose not to design the according U primers (that would amplify the non-methylated bisulfite-converted DNA) as we do not assess methylation in a quantitative way. After *in silico *assay evaluation, 48 selected MSP primers (including the *ACTB *control; Additional file 3) were empirically validated on the Roche LightCycler 480 (LC480) using the *in vitro *methylated HCT-116 DKO (positive control), the HCT-116 DKO (negative control) and NB cell lines. Based on melting curve and amplicon size analysis, all assays were considered amplicon specific. The MSP assays were tested on 89 samples, selected from the previously described patient groups (31 LR-SURV patients, 28 HR-DOD patients and 30 HR-SURV patients; Additional file 3). A no template control (NTC) sample was loaded as well. For all samples, 500 to 1,000 ng DNA was bisulfite-treated (EZ DNA Methylation Kit, Zymo Research) and eluted in 40 µl elution buffer. Prior to MSP, bisulfite-treated DNA (BT-DNA) was amplified using the EpiTect Whole Bisulfitome Kit (Qiagen), starting from 100 ng BT-DNA. After amplification, the yield was determined by the Qubit 2.0 fluorometer in combination with the Quant-iT PicoGreen dsDNA BR Assay Kit (Invitrogen). The MSP was performed on the LC480 and plates were prepared using the Tecan freedom Evo robot, using a design that assures that all samples were tested for the same assay in the same run [65]. MSP amplifications were performed in 10 µl containing 5 µl LC480 SYBR Green I Master Mix (2×; Roche, Vilvoorde, Belgium), 1 mg/ml bovine serum albumin (Roche), 1 mM MgCl_2 _(Roche), 125 nM forward and reverse primer (IDT, Leuven, Belgium), sample (20 ng amplified BT-DNA) and nuclease-free water (Sigma). MSP conditions were as follows: activation for 10 minutes at 95°C, 45 amplification cycles (10 s at 95°C, 30 s at 60°C and 5 s at 72°C), followed by melting curve analysis (5 s at 95°C - melting curve from 60 to 95°C) and cool down to 45°C. Afterwards, the size of the amplicons was determined using the Caliper LabChip GX. A MSP assay was considered methylated if (1) its Cq value <35 (calculated by the LC480 software using the second derivative maximum method), (2) its melting temperature (Tm) differed no more than 2°C from that of the positive control sample, and (3) the amplicon length differed no more than 10 bp from the band size of the positive control sample. In addition, the band height, as determined by the LabChip GX software, was required to be higher than 20.

### mRNA expression profiling

The mRNA expression levels of *CNR1*, *GRB10*, *KRT19*, *PRPH *and *QPCT *were profiled on the NB SIOPEN/GPOH cDNA library generated from 366 primary NB tumor samples (Additional file 5) [6]. For each DNA methylation marker a qPCR mRNA assay was designed and validated *in silico *and *in vitro *(Additional file 5) [66]. PCR plates were prepared as described in the previous section and RT-qPCR was performed on the LC480 as described in [6]. Relative gene expression levels were then normalized using the geometric mean of five reference sequences (*HPRT1*, *SDHA*, *UBC*, *HMBS *and *AluSq*) [67]. For *HIST1H3C *and *ACSS3*, a qPCR mRNA assay (Additional file 4) was designed and tested on 31 NB cell lines on which the corresponding MSP assay was tested as well. Here, qPCR amplifications were performed in 5 µl containing 2.5 µl SsoAdvanced SYBR Green Supermix (2×; Bio-Rad), 0.25 µl forward and reverse primer (5 µM each) and 2 µl cDNA sample (corresponding to 5 ng cDNA). Relative gene expression levels were normalized using the geometric mean of the reference sequences *SDHA*, *UBC *and *AluSq*. All RT-qPCR data analysis was done in qbase^PLUS ^version 2.0 (Biogazelle, Ghent, Belgium) [65]. Logged and normalized qPCR data can be found in Additional file 4 and 5.

### Statistical analysis

Statistical analyses were performed using IBM SPSS software version 19.0. All statistical tests were two-sided and *P*-values <0.05 were considered statistically significant. Differential methylation across the prognostic groups was determined by the Chi square test. The relationship between the methylation status and NB risk factors was determined using Fisher's exact test. Univariate survival analysis was performed with the Kaplan-Meier method and log-rank statistics (Mantel-Cox) to determine the impact of methylation status on EFS and OS. EFS was defined as the time between initial diagnosis and relapse or death of disease, or time between diagnosis and last follow-up if no event had occurred. OS is the time to disease-related death or last follow-up. Hierarchical clustering and leave-one-out decision tree analysis were performed using R 2.13.0 (rpart package). The relationship between logged mRNA expression levels and the prognostic groups, OS status and NB risk factors was determined using the nonparametric Kruskal-Wallis test or Mann-Whitney test. Hazard ratios between logged mRNA expression data and survival were estimated using the Cox proportional hazard model. Kaplan-Meier curves were created by dichotomizing the logged mRNA expression data, using the median mRNA expression value as a cutoff. For *HIST1H3C*, the relationship between logged mRNA expression levels and the methylation status of the gene, and the *MYCN *status, was determined using the Mann-Whitney test. For all the above mentioned statistical tests, multiple hypothesis testing correction was performed (Benjamini-Hochberg method by using the R function p.adjust).

## Abbreviations

BT-DNA: bisulfite-treated DNA; CI: confidence interval; DAC: 5-aza-2'-deoxycytidine; EFS: event-free survival; FDR: false discovery rate; GEO: Gene Expression Omnibus; HR: high-risk; HR-DOD: high-risk deceased patients; HR-SURV: high-risk survivors; LR: low-risk; LR-SURV: low-risk survivors; MBD: methyl-CpG-binding domain; MBD-seq: massively parallel sequencing after capturing with an MBD; MeDIP: methylated DNA immunoprecipitation; miRNA: microRNA; MNA: *MYCN *amplification; MSP: methylation-specific PCR; NB: neuroblastoma; NGS: next-generation sequencing; OS: overall survival; TSS: transcriptional start site.

## Competing interests

The authors declare that they have no competing interests.

## Authors' contributions

AD performed the high-throughput MSP study, was involved in data analysis and drafted the manuscript. MO performed data analysis of the re-expression data and sequencing data, supervised the work and helped draft the manuscript. JH carried out the DAC experiments and the initial high-throughput MSP. KDP was involved in advanced data analysis and helped draft the manuscript. GVP was involved in optimizing the high-throughput MSP experiments. WVC optimized the MBD- seq techniques and was involved in the data analysis of re-expression data. RL, JHS, RN, RLS, AVD, GL and TVM were involved in sample collection and management and helped to define the clinical questions. JV was involved in the mRNA expression profiling experiments. FS and JVa supervised the entire study and helped draft the manuscript. All authors read and approved the final manuscript.

## Supplementary Material

Additional file 1 **Clinical patient annotation, MSP assays and results on the BioTrove discovery platform**. Click here for file

Additional file 2 **Visualization of the protocadherin beta gene cluster and the *HIST1H3C *promoter region in the Integrative Genomic Viewer**. Eight neuroblastoma cell lines (SK-N-AS, CLB-GA, SH-SY5Y, SJNB-1, CHP-902R, IMR-32, LAN-2 and N206) and the MBD-seq results are displayed. Click here for file

Additional file 3 **Clinical patient annotation, summary of clinical characteristics, MSP assays, results and summarized results (per clinical parameter) on the LC480 platform for 89 NB patient samples**. Assays differentially methylated between prognostic groups and between neuroblastoma risk factors are discussed in detail, as well as extended analyses on the MSP data (hierarchical clustering (heatmap) and survival analysis according to the number of methylation events (Kaplan-Meier plot)). Click here for file

Additional file 4 **Quantitative PCR and MSP assays for *HIST1H3C *and *ACSS3 *and matched results (expression levels - methylation call) for a panel of 31 NB cell lines**. Click here for file

Additional file 5 **Clinical annotation, summary of clinical characteristics, qPCR assays and results of qPCR experiments on 366 NB patient samples (SIOPEN/GPOH cDNA library)**. Boxplots of the expression levels for *CNR1*, *GRB10*, *KRT19*, *PRPH *and *QPCT *in each of the five different NB stages (stages 1, 2, 3, 4 and 4S). A Kaplan-Meier plot shows overall survival according to the relative mRNA expression levels of *CNR1*, *GRB10*, *KRT19*, *PRPH *and *QPCT*. Click here for file
