# Supplementary material for: Genome-wide promoter methylation analysis in neuroblastoma identifies prognostic methylation biomarkers
Source: Genome Biol. 2012 Oct 3;13(10):R95. doi: 10.1186/gb-2012-13-10-r95 (PMC3491423; doi:10.1186/gb-2012-13-10-r95)
Supplement: Additional file 1 — Clinical patient annotation, MSP assays and results on the BioTrove discovery platform. [file gb-2012-13-10-r95-S1.pdf]

Part BioTrove clinical annotation: Patient characteristics of the samples used for the initial high-throughput MSP screening on the BioTrove OpenArray. PatientID is a unique patient number, the group indicates the risk. Clinical characteristics given are the age at diagnosis in months, International Neuroblastoma Staging System (INSS) stage, *MYCN* status (0 is non-amplified/single copy and 1 is amplified), follow-up time (FU) in days after diagnosis, and overall survival (OS) and event-free survival (EFS) time in days after diagnosis. OS indicates whether the patient was alive (0) at the last known FU or died of disease (1). Similar for EFS, indicating events, such as relapse or progression. Empty cells represent missing values.

Part BioTrove assays: MSP assays used in the initial high-throughput MSP screening on the BioTrove OpenArray. For each tested gene, the assay name(s) and corresponding forward and reverse primer(s) (5’ to 3’) are indicated, as well as the genomic location of the amplicon on the hg19 reference genome.

Part BioTrove results: Results of the initial high-throughput MSP screening on the BioTrove OpenArray. For each assay and sample, the methylation call (dark blue is methylated, green unmethylated) is given. Samples are subdivided into two prognostic groups (HR-DOD and LR-SURV). The specificity and sensitivity of the assays is indicated as well. Assays selected for further testing are indicated in yellow. HR-DOD: high-risk deceased patients and LR-SURV: low-risk patients alive for at least 1000 days follow-up.

PatientID	Group	Age at diagnosis (months)	INSS Stage	*MYCN*	FU time (days)	OS time (days)	EFS time (days)	OS	EFS
589	HR-DOD	421	4	0	4569	4569	2958	1	1
389	HR-DOD	42	4	0	581	581	449	1	1
7	HR-DOD	65	4	0	3364	3364	2539	1	1
911	HR-DOD	64	4	1	300	300		1	1
N432	HR-DOD	36	4	1	549	549	480	1	1
811	HR-DOD	67	4	0	944	944	209	1	1
499	HR-DOD	47	4	1	268	268	254	1	1
857	HR-DOD	71	4	0	877	877	728	1	1
3	HR-DOD	20	3	0	639	639	438	1	1
N159	HR-DOD	57	4		653	653	553	1	1
498	HR-DOD	23	3	1	531	531	450	1	1
510	LR-SURV	4	4S	0	2207	2207		0	0
711	LR-SURV	17	2	0	1885	1885		0	0
278	LR-SURV	15	1	0	3404	3404		0	0
774	LR-SURV	4	4S	0	2182	2182		0	0
1383	LR-SURV	0	4S	0	2122	2122		0	0
397	LR-SURV	18	2	0	3555	3555		0	0
518	LR-SURV	0	4S	0	2050	2050		0	0
58	LR-SURV	10	2	0	4858	4858		0	0
824	LR-SURV	1	2	0	997	997		0	0

BioTrove clinical annotation

Name	Forward primer	Reverse primer	Genomic location
TNFRSF10D_23780	TCGGGGTATAAAAGCGGAG	ATCAAAAATCGTCCCCGTAA	chr8:23021486-23021578
KRT19_83159	GGTCGCGGGTATAAAAGGC	CATAACGAAACGAAACACGAAC	chr17:39684496-39684598
TNFRSF10A_65529	GGTATTTAGTAGGCGTTCGGTG	ACTCGCCCGATAATAACGAAA	chr8:23082862-23082978
RARRES1_57581	GCGAAATCGTAGGGGAAAC	ATAAAAACGCCTCCCCGAAA	chr3:158450497-158450595
KRT19_61596	TTATAGTTATCGTTAGTCGTCGGT	ACCCCCGTAAATACTAAACGC	chr17:39684382-39684491
CASP8M_1	TAGGGGATTCGGAGATTGCGA	CGTATATCTACATTCGAAACGA	chr2:202123060-202123380
CYGB_83388	TCGTGTGGGTAAGAGTTAATGTCG	CTCCGCCGTAACCTACGAA	chr17:74533911-74534004
CTDSPL_23804	GTTTTGGGAGAGGCGGTTC	TCATAATAACGAAACGACGACC	chr3:37903464-37903550
FBN2_18150	TCGGAGTTTTATAGGGTAACGAA	CTCTTACTAACCGCACGCC	chr5:127873815-127873902
PDLIM4_4	GGCGTTTAGGTTAATTTTTCGT	CGATCCCATATCTAAAACCGA	chr5:131593549-131593644
CXCR4_70046	GCGTGGGTGTAGTGGGTAGTC	TAAAAACCTCCAAACGATAAACACG	chr2:136874383-136874463
RBP4_24106	GGTCGTTTCGTTGTTTTATAGC	GCGTTATACAAATACCCCCG	chr10:95361003-95361106
RBP1_83281	TGCGTTCGTTTTCGTTAAGC	CTAAATTCCCAACGCGCAC	chr3:139258517-139258597
NPTX2_57773	TTTTAGTTTGTGACGTTCGCGTT	TAAAACTCTCGAAAACCTCGACT	chr7:98246269-98246349
PPIC_69739	CGGTTGTTGTTATTTTTCGTGT	CTCTCGATCACCTTAACCGTC	chr5:122372192-122372305
GREM1_83135	TCGGTTTCGTTGATTTCGC	GCGCTCACCTATCAATACGAC	chr15:33010259-33010370
GATA4_13295	GGTATTGTTATTTTGCGTTTTC	CCCGAAACAAACTACACGAC	chr8:11562180-11562274
CSRP1_12560	TAGTGGCGCGTAGAATCGT	CTAAACACAATTAACTTCCGCGT	chr1:201475888-201475990
HPGD_82365	TATTGGTAGGGGAGCGTTTC	GATAAACGAACTCCGCGTCT	chr4:175443626-175443719
NPY_18477	TTTTATTCGATGGCGTTGTC	AACAACTAACGATCTCCCTACGA	chr7:24324196-24324288
CDKN2B_27347	TTTTTATTTTGTTAGAGCGAGGC	ACCGAACTCAAAACCGCTC	chr9:22009225-22009309
FAS_18143	TTTTTGATTATCGGGGTTTTTC	AACCAAATCACTCGTAAACCG	chr10:90750369-90750484
S100A6_83933	AGAATTTATCGGTAGGGAGGC	ATAAAACCAATCGAACTACGACA	chr1:153508415-153508496
IGFBP1_82382	GATTGTTTAGGTCGGCGTG	GAATAACCTCCGAACACGAA	chr7:45928298-45928408
PRTFDC1_82627	GTTTTTAGAGTAGTACGCGGGG	ATAACTCGCTCCTACAACGTCC	chr10:25241095-25241214
EOMES_82239	TTCGTCGTTGTAGTCGTCGT	CGAAAAACTAAAAATAATACCGCTC	chr3:27763337-27763440
LATS2_82421	GAGAGGTTTTGGCGAGTTATC	ACCGTACTTCCTACCTCCGAC	chr13:21635825-21635906
TMEFF2_8419	AGTCGGAGGATAGGAGGAGAC	AAAACGACTACCGAACTACGAAA	chr2:193059529-193059633
DLC1_9164	CGGGAGATAGTTCGTTTCGAT	GAAAAATCCGTAAACCGACGC	chr8:12990704-12990806
NQO1_52551	TATAAGGTTGTAGTCGGAGTCGT	CTCTAATACAATCCGAAACGCTA	chr16:69760344-69760451
SST_23808	TGGTTGCGTTGTTTATCGTTT	TTACCTACTTCCCCGCGAC	chr3:187387934-187388048
SLIT3_23619	AGGGGTATTTATAGGCGTTTAGC	TACCTACTCCGCTACCAACGTAA	chr5:168727504-168727600
HOXA9_77983	GGATGAGTTGAGCGTTGGTC	CACGAACTAAAATCGAAATACTCG	chr7:27204915-27205011
TNFSF9_30093	TAGGGTTTAAGGTAGTACGCGGT	AATCTCTCGTCATAAAATACGCC	chr19:6531036-6531140
NF2_23823	TGAGGACGGTGATAGTTACGC	AAACCCCTAAACGACAACGA	chr22:29999568-29999681
NAB2_69824	GAGTTTGGATAGCGGTGGATAC	CTACCCTTCTCGATACCCGAC	chr12:57482944-57483032
NADK_83330	TTGGTCGTTAGAGGTGGGC	AACTACGACAACGCTACGCTC	chr1:1710436-1710542
TBX3_83233	ATATGATTAGAGGGCGGGC	CCAAAACGAACGAACGAAC	chr12:115122200-115122311
FOXE1_13317	CGGAGAGTTTAGGGGATCGT	CTCTATCTACACCGCGCCA	chr9:100615988-100616099
DSC3_52554	GTCGGGTAGGGTTAGGAGAAC	CTTAAAAACAAACAACGACGAAA	chr18:28622632-28622738
PCDH10_82547	TTTCGTTTTTGCGTAGCGT	TTAACCAATCATCGACAACTCG	chr4:134070348-134070428
KRAS_82400	TTTTTGTGGAGATCGGTTAGC	AACCGCGACGAACTATACG	chr12:25404255-25404335
SOCS3_53602	GCGTAGTTTTAGGAATCGGGG	ACCGAAACGACAACAACGAAA	chr17:76356116-76356228
TP73_1	CGGGTTTTGTAGGAGCGAC	AACTAACCGCGTCCAAATCG	chr1:3568823-3568913
ADAM23_66271	TTCGTCGGTTATACGGAGC	GACAAAACTACAACCCGCCA	chr2:207308550-207308647
PPIC_69743	CGAGTTTGAATTTGGGAGGAAC	GAAACTACCGACCGACTACGC	chr5:122372395-122372503
MYST4_82478	GTAAAGTTTGGGCGAAGGAC	AACGAAAAATATACCCGCCA	chr10:76584918-76585025
FABP5_82270	TTATTGGTCGTATAGCGTCGT	AACAACGTACTATAAAACACCCGA	chr8:82192702-82192783
MAL_74689	TTGTGGCGGTGGTTTAGTTTC	TTCATTTTTCCGCTAAATACGTT	chr2:95690978-95691062
SLC44A3_83942	GGGTACGGTGTAGCGTCGT	AACGAACCCAATAAACCGC	chr1:95285787-95285867
TP73_72009	TTGTCGTTTGGTTTTTGTTTTAC	TCGCTCTACCCAATCAACG	chr1:3567209-3567290
TNFRSF10C_9282	TTTGTTGTTAGTGAGTTTCGGTC	ATCCCTACCGTATCCCCGTCT	chr8:22960684-22960777
CTCFL_23792	TTCGGGTTAGTGTATATTCGG	ACTAAATCTAAAACCGTACACGA	chr20:56099721-56099801
PCDH20_23327	GCGTTATTGTGTTTAATTTCGAGT	ATAATTTCCTACCCCGAACG	chr13:61989460-61989572
THBD_82800	GTCGGAGTAGAGGGGTATAGGAC	AAAACTACGCGCAACGACA	chr20:23030201-23030289
MORN1_83172	AATTATTTTCGTTTTCGGCGT	GTTTCCGCCGCTATCGTCT	chr1:2323131-2323211
ROBO3_64828	GAGGAGGGTACGAAGAGGTATC	AAACCCGTAAACTAAAAACCGTAA	chr11:124735301-124735418
MRC2_9206	TTCGTAGTTTAGGTTTTATTACGTC	AAAATCCCCAAACGCTACGTC	chr17:60704638-60704742
NPTX2_57779	GCGTCGTTTTGTATGGGTATC	CCCGATAACCGCTTCGTAT	chr7:98246839-98246928
GATA4_2	AGGTTAGTTAGCGTTTTAGGGTC	ACGACGACGAAACCTCTCG	chr8:11561654-11561760
DNAJC15_2	TTGGGTAACGTGTTTCGTGT	ACTCCCTAAAAATCCGCGT	chr13:43597490-43597604
IGFBP3_54583	TGTAGACGATAAGGTGATTCGG	CTCGAAAACGTACAACTCGAAA	chr7:45961026-45961109
RASSF1A	GCGTTGAAGTCGGGGTTC	CCCGTACTTCGCTAACTTTAAACG	chr3:50378248-50378323
CCNA1_1	GTTATATGTATCGGGAGTCGGG	CGACTAAAACGCGACGAAA	chr13:37005782-37005893
TNFRSF10D_23781	AAGAGGAGAGATAGGCGGTTC	AAAACTAAAACGCTACCCGCT	chr8:23021780-23021893
SLC44A3_83941	AGTTGAGTCGGCGTCGTTA	GCCACTACACTATTACTCGACC	chr1:95285531-95285641
DNAJC15_1	ACGAGGTTTTATTATGTCGGTT	CGAATACGATAACTCACGCTA	chr13:43597084-43597174
EGFR_74891	GATTTTCGAGAGGGTTTCGTAG	CCGAACCTTAAAACCAACGTC	chr7:55086203-55086306
SPINT2_23336	TTTGCGTTTGCGTTTAGTTC	GCCTATTAAAAACGACTCCCG	chr19:38755119-38755208
HTATIP2_82372	TACGGTATTTTCGGTTCGGT	CCGCCCTACTCCTACTACGTC	chr11:20385406-20385521
NAB2_83179	CGTGGGCGGTATAGAACGAT	CCGAAATAACCGAAACCCG	chr12:57482521-57482601
DSC3_52537	TTTACGTTTTTCGGTTGTTTC	TACATTAAAATACACCGCGCA	chr18:28622958-28623078
FOXE1_13314	TTTGTTCGTTTTTCGATTGTTC	TAACGCTATAAAACTCCTACCGC	chr9:100615353-100615448
DIRAS3_9259	CGTAGTTTTTAATGTATTCGTCGT	TTTCCAAATACGCCTACGTCT	chr1:68517353-68517455
ZIM2_82923	TAGTTCGGGTTGTTGCGTT	CTAATACCCCCGATTTACTACGA	chr19:57351593-57351711
PRDX2_83202	TTGTTATTAGTATTTTACGAGCGAG	GCAAACCTAATCGCCCGAC	chr19:12912285-12912403
CTDSPL_23803	GGTGGGTGTGTTTCGGTTC	ACCCTATATCGACCCCGCT	chr3:37903189-37903288
SYCP3	TGTATTCGTAATTCGTTTCGGC	CAAAACGCAAACGTAAACTAAATAACG	chr12:102133313-102133421
HPGD_82367	GAGGTTTTGAGTCGGTTTGC	GACTATATCACCTACCCCCTAAACG	chr4:175443763-175443849
CDKN1C_83412	ATTGGGAAGGTTTTACGGGC	CTCCTCTCTCGCTACCCGC	chr11:2906651-2906757
NQO1_82535	ATTTTGGTCGGAATTAGGTTTTC	AATACACCGTACACTACACACGC	chr16:69760395-69760480

BioTrove assays

Name	Forward primer	Reverse primer	Genomic location
IGFBP3_RadRes	ATGCGAGTATTGCGGTTGGGC	CCTACAACCGAAACACGCTACT	chr7:45960743-45960869
EGFR_23302	TAGGAGCGTTGTTTCGGTC	CACGACCCCCTAACTCCGT	chr7:55086905-55087006
TPM2_83253	CGGTTTTTATCGTTTGTCGG	TCGACCTACTCGACGCGAT	chr9:35689737-35689857
SLC4A11_82709	AGTTTCGTTTTGTTTATTTTCGAGT	AAAACTCTACTATCGCCGTCT	chr20:3219429-3219526
TNFSF9_82827	GGGGTACGTTTTATTTGTCGT	GTATCCGAAACTCGCGCCT	chr19:6531209-6531328
CEPBD_GT_2	ATTTTCGAGGAGAGTAGCGAGA	AAAACCAAACGTAAAAACAACGA	chr8:48651275-48651384
CDKN2B_27345	TTAGAAGTAATTTAGGCGCGTTC	AAACCCCGTACAATAACCGA	chr9:22009111-22009201
EOMES_82240	AAGTAGTTAGTTGTCGTTTGCGTT	CGAATAAAAATACGCGCCA	chr3:27763722-27763818
F2R_74866	AGAGATTTTTATTGTACGTCGGAG	AACGATTAACGAAACGAAACGA	chr5:76011867-76011957
NPY_18473	AAGTAGGGATCGGGTATTGTTC	GCGTAAACGAAACAACGCA	chr7:24323503-24323588
ID4_56956	GTTATTTAGGTTGTGGTCGTGTTC	ACGATAAACCCGATACGCC	chr6:19837991-19838096
DBC1_23888	TTTTGGATGTTCGTGCGTAT	CCCGAATAAACTAAAACTAAACCG	chr9:122131953-122132039
SLC4A11_82719	TTCGGGTTATACGGGGGTC	CAACGACTCTAACTTCCAAACGA	chr20:3218744-3218824
TRIM59_82865	GTTTGGAAAAGTGGCGTTC	GAAATCCGAACTTAAAACTACCG	chr3:160117628-160117746
PCSK6_56483	ATAGGATTGCGGAGGCGTT	ACCAAATAAATACGACCGACC	chr15:102029497-102029583
TPM2_82850	GTTTTTCGAGAGATATTTAATTCGG	ACGACAACTACCCGAACGC	chr9:35690281-35690395
CDKN1C_1	TTTATAGGTTAAGTGCGTTGTGTTC	TCCGATAATAAACTCTTCTACGTC	chr11:2906870-2907006
CEPBD_GT_1	TATGGAGTCGATGTAGGCGTTG	CTTCTACGAACCGAACCGAAC	chr8:48650478-48650614
PRDX2_82604	GGTATAAAAGGTTTCGCGGT	AAAAACTAAACGCACGAACGA	chr19:12912563-12912647
RPRM_1	CGGGTTGGTTTAGTTTCGTT	TCTTCTAAAACCGTCCGCTC	chr2:154335270-154335365
FBXO2_83916	CGTGAAAGTTTGTCGCGTT	CTAAACCGCTCGCTACGAC	chr1:11714253-11714364
ADAMTS5_82116	CGGAGTTTGGAATTTTTATAGTCG	CACCGAACTAACAACTAATACGC	chr21:28339098-28339179
EDNRB_3	GTCGGGTGTTATATGGTGCGT	AAAAACAATCCTCGTCCGAAA	chr13:78493861-78493977
RBP4_24108	GATTTTAGAAGGTAGACGGAGGC	TAACCTCACAAAACGAAAACGA	chr10:95361343-95361443
TNFAIP2_83961	TAGCGGAGTTCGAGGACGTA	GAATAAACGCCAACCCCGT	chr14:103592969-103593088
NNAT_82085	GAATTATGGCGGTAGTGGC	TTACCTACAACAACACGCGAA	chr20:36149728-36149809
DMRTB1_12825	GCGCGGTTTATTTTAGCGT	ATACGCACCATTTTATCGACC	chr1:53925051-53925149
PENK_18538	ATTTAGGTTATTACGGGTTCGC	AACTACCTCCGACCCACCG	chr8:57358226-57358311
ID4_56952	GCGAGTAGGGTTTAGGCGTT	AAAACTACGAAAATATACGACCGA	chr6:19837124-19837214
RARB_60785	CGATGATAGGTGGAAAGAGTAACG	GAAACATAACCACCAACGACC	chr3:25215883-25215988
DMRTB1_83381	CGCGTAGAAGGTGTTTAAGACGTAG	CGAAAACGAAAACGACGACC	chr1:53925278-53925388
SLC25A31_82698	TTTATTATGTTTTTGTATCGCGTTT	ACGATAACGCCCATCGAAC	chr4:128651808-128651903
INHA_13228	TGGAGTTAGGCGGTTGTTTC	GATTCCCCCACCCTCGTTA	chr2:220437254-220437384
IGFBP1_82385	ATATTTAGTTTTTAGCGTGCGG	ACGATAACCGATACTCGCTA	chr7:45927987-45928098
DAZL_22631	GTAGTTGATAGGCGCGAGG	AAATTCGTAAAAACCTCAACGTAA	chr3:16646022-16646131
PDLIM4_60076	ATGGGTCGTAGGTGTGTTAGTC	AAACGATACTCCAACCCGAAA	chr5:131592952-131593057
HLA-B_82358	GTTTTAGGTTTCGGACGGG	AAAAATCCCCAACGCTACG	chr6:31325123-31325207
CCNA1_83097	GTTATGGCGATGCGGTTTC	ATCGACCAATCAACGACGC	chr13:37006314-37006406
RAMP3_82633	TAGTCGCGTTTTTAGCGGG	AAAAATTACGAACGCCGCA	chr7:45197376-45197462
ZIM2_82925	GGTAATCGTAGTTTGATTGGTACG	AACTTCTCCGCAAAAACGA	chr19:57352125-57352234
FAS_18144	GTGTTTCGTGTTAGTGTTCGTTT	TCGTCTCTAATCTCGCGCA	chr10:90750397-90750517
AHR_82118	TTTTGTTATTTAGTCGGTGCG	TAACGATCCGAAACACGCT	chr7:17338238-17338330
S100A6_83934	TTGTCGAGTTACGTGTTACGAAG	CCACTACACGCCTAATAACGA	chr1:153508676-153508760
EPHB2_54970	CGGTCGTTTTTCGTTTTTATC	CTACTACTACTACCGCTACTCGCC	chr1:23037502-23037583
TNFRSF10A_9277	CGTTGGGATTAAGTGGTAAAAC	CCTCTATAACCGCCCTTACCG	chr8:23082710-23082811
LATS2_82419	ATTTATTGTGGTGTCGAGGTTTC	AAAACGCAAAACTCCGTCC	chr13:21635316-21635397
MYO18B_24620	GAAAGGTCGGATTTGTTTTTC	ACCATCTCATCACGCCTCG	chr22:26157062-26157175
DPP4_24687	TTCGGGTTTCGTATTTTGC	ACACTAACATCCCGACCGA	chr2:162930720-162930815
CSRP1_12558	TTTTTAGGTTTCGGGAAGGTC	GATAACGACGACAAAACGCA	chr1:201476383-201476493
RARB_60784	GAGAGGTAGGTAGCGAGCGTAT	ACCAAAAACAACGTCCCGACT	chr3:25469475-25469591
NADK_83183	TCGACGGATAGTTTCGAGG	CACGATATAAACCGAACCGAA	chr1:1710393-1710476
GREM1_29777	GAATTTGGTACGATTTTACGGAG	ATCTAAACTTTCCCTATCGACCG	chr15:33009752-33009860
ADAM23_A	GAGGTTTTAAGTTGGCGGAGC	ACTCGAAACTAAACGACGCCC	chr2:207308329-207308441
MRC2_82455	TTTCGATTCGGAGGAGGAC	ACGCTCAATAAACGCGAAA	chr17:60705039-60705152
RARRES1_57577	GTCGTTTGTTGTAGGAGTTTGC	CTACTACTATTACTCGCCCCGAT	chr3:158450013-158450123
CAV1_58524	TTCGTTAAAGGTTTGTTTTGTTC	TCTCTACTCGCTACGAACGCT	chr7:116164859-116164973
CRBP_1	TTGGGAATTTAGTTGTCGTCGTTTC	AAACAACGACTACCGATACTACGCG	chr3:139258429-139258529
SPINT2_23333	GAGGGTCGTTGAGTGTCGTAG	CTTCAATATAAAAACGCCCGA	chr19:38755270-38755363
CYP1A1_23831	TATTCGTGGTGGTGTTGAGC	AAAATATAAAAATCGAACCGACC	chr15:75015103-75015197
WNT5A_65596	CGAGTAGGAAGGAGGTAGCGTT	AAATCCTATACGCGAAACACG	chr3:55515157-55515251
SLC25A31_82696	GATGAGGCGTTAAATAGTCGTTT	CCTTCATCGTAACTCCCGT	chr4:128651636-128651756
ZBTB25_83276	TATTAGATACGCGGTTACGTGG	ATAACGCCGACTCACGCAC	chr14:64970452-64970543
MORN1_83171	TTTTAGAGATTGCGCGGGT	TTAACGACCAACAACGCCC	chr1:2322991-2323083
TSHR_83270	AGTTTGCGTATTGGGCGGT	ATTCGTCTCCACCCTACGA	chr14:81422094-81422192
ESR1_2	CGGGAGTTTAGGAGTTGGC	CTACCCCGAAAACCTACGA	chr6:152128804-152128901
TCEB3_83953	TCGCGTTAGTGATAGCGATG	CGCTTACCTTCTTAAAATCCGAA	chr1:24069976-24070075
MAL_74692	GGTAGGGTGTTGTTTTTCGTC	AAAATCTACGCGAAATCTAAACG	chr2:95691475-95691575
HCRTR2_82338	GAGTTTTAGTTGAGTCGGACGTAG	TCTTTACAAACGATATTACACCGAA	chr6:55039076-55039193
ATM_9746	TTTAATATAAGTCGGGTTACGTTCG	ATACGACGCAAAAACTATCGC	chr11:108093225-108093342
EMP3_82232	ATTTGTGAAATTCGGTAGGGC	CTCAAATAATCTACCCGTCTCG	chr19:48828134-48828216
RUNX3_2	TATTTATTTTGAAGGCGACGG	TAACGAACCACGCAAACGA	chr1:25256070-25256184
CTGF_31218	TCGTTGAGAGGAGATAGTTAGTGC	GATAATCGAAAATAAAAACCGAA	chr6:132272383-132272496
GPR50_82319	TATTTTTATGTTTTGCGCGATG	TTCCGAAACTTCTTATTCTTCGT	chrX:150345276-150345375
TMEFF2_8421	CGGGGAAGTAGTAGTTAAATTCG	GCAACTTCTCAACGAACGACC	chr2:193059381-193059484
NNAT_83189	TTTTAGTGCGTTCGCGTTT	ATAACGAATTTTTCTTACCGCGT	chr20:36149997-36150108
MAGEB2_71596	CGTTAGAATAGTGACGTTCGGT	GAAAATCAAAATAACTCACATCCG	chrX:30233556-30233639
MSH4_27679	TGTTGTTTCGGTCGTTCGTAG	AAAATCCCCGCTACAATTTCG	chr1:76262749-76262866
TEX14_56176	TTGTGGGTTAGTTTTAACGGTC	CGAAATACGACTCCCGAAA	chr17:56769364-56769449
TRIM59_82871	GATTTTTCGGAGTAGCGGG	ATCTTTTACTTAACGCCACACGA	chr3:160168002-160168100
NF2_61304	TTTTTGGGGCGTTATATTCGG	CACTTAAAAACTAAATCCGAAACG	chr22:29999128-29999228
PCDH20_23326	ATATTGTAATTTGTCGGGGTC	AAATATAAAACGTCTACATCGTCC	chr13:61989111-61989214

BioTrove assays

Name	Forward primer	Reverse primer	Genomic location
GREM1_29775	AGGTTTTGTATGTGACGGAGC	ACGACTATTACAACCTTCCTCGT	chr15:33023126-33023224
CASP8U_1	TAGGGGATTTGGAGATTGTGA	CCATATATCTACATTCAAAACAA	chr2:202123060-202123381
CTCFL_23794	TTTTTATTTTCGCGTTAGTTCG	ATACTCGACCCACTCCGTCT	chr20:56100102-56100201
HCRTR2_82341	CGAAGTAGATGATTAGTTGCGATA	TTACAACATTAAACGAAACCGAA	chr6:55039350-55039442
SST_83224	AGGTAGTAGCGATGGTTTCG	AAAACGCCTCCTAACCTAACGTC	chr3:187388135-187388242
PCSK6_56480	TCGGGTCGTTTAGTATTTGTATC	CTAACGCTACCTACCGCCT	chr15:102029628-102029711
CYGB_17997	TCGTAGTTGGTATAGAGTCGGG	CGAAATAAAAATCGAACGCA	chr17:74533508-74533607
F2R_31099	TTTCGTTCGCGTATTTTATTTTC	ACTTCAATCTATACGACCCGCT	chr5:76012166-76012256
TADA3L_82757	GATAAGTCGAAGCGGACGA	CGAACGCCAAAAACTACGA	chr3:9834370-9834461
WNT5A_83273	TTCGCGTATAGGATTTTAGCG	AACTACGCTTCTCCTCCGT	chr3:55515053-55515173
TCEB3_83956	GTTTTCGTCGTCGGTATTTTC	GCGATAACCTCATTCCGTCT	chr1:24069633-24069732
TNFRSF10C_9283	TTATAGTAGCGTAGTGGGTAGCGT	CGAACAAAAACTAACTCTTTCCG	chr8:22960155-22960279
DIRAS3_9258	GCGTATTATCGTTTATCGAGCG	ACCAAATACTACCGTCAACGACT	chr1:68517452-68517557
THBD_82802	GTCGATTTAGAGGCGTCGG	GAAACCACCTAATAACAATACGCT	chr20:23029874-23029975
TADA3L_82759	TACGGAAGTGGTTAGTTTCGC	GTAAAAACTCTCTACCCCGCTC	chr3:9834659-9834774
DAZL_18008	GTCGGGAATTGTTGTTTGTC	ACCGAATTTCACCCACGAA	chr3:16646644-16646738
SLIT3_23622	TATTGAATGGAGAGAGCGAGC	ATAAAACGTAACGCCCGAAA	chr5:168728027-168728144
ESR2_74878	CGTAGGTGTGTGATAAAGTCGG	CTACGCGAAAACGTAACGAAC	chr14:64805138-64805227
MYST4_83331	TTATCGAAGGAGTTGGCGA	AAAAATAAACGCGAACGAAC	chr10:76585748-76585829
VASH1_83272	TTAATTGCGAGAATAATGACGG	TCTACAACCGCTAATCCGAAC	chr14:77228772-77228871
SOCS3_53605	TTTAGGGGAAGTTCGAGGGAC	ACGACGAAACGACCAACGATA	chr17:76355893-76355995
XYLB_65485	GTTAGGCGATTGTATTTGTTGC	TTACCCGAAAAACCATAACGA	chr3:38388345-38388431
FABP5_82271	GTAGAGGCGTCGAAACGTG	CCTCTTACCAAAATACGCGAC	chr8:82192511-82192622
TBX3_82767	TCGGTTCGTTTGTTCGTTG	GCTCTAATTCAACCCACGTCT	chr12:115122304-115122424
HOXA9_2	GTTGCGGAGAAAGATACGAG	CGAACGAAACATCACGACC	chr7:27208970-27209060
DLC1_83387	TGGAGAAATTAACGGGGACG	CAACGAAAAACTAAAACGCCA	chr8:13372115-13372196
XYLB_65481	CGGAGATGTTTTGTTAATGTTATTC	TAAACACCGAAAATCCTACCGA	chr3:38388598-38388708
FABP3_83914	CGTGGGTATTAGTTTAAGGGCGT	GCATACCATAATAAATATCTTCCCG	chr1:31846228-31846333
PCDH10_83191	GTTAGCGTTTCGGGATTTGC	ACCGCCAAACAAACACGTC	chr4:134070224-134070344
FABP3_83912	GATGCGGTAGGAGTGTTGC	CGATAAACAAACCGCGAAA	chr1:31845705-31845790
PCDHB6_82558	TATAATTTGTTTGGCGACGATT	ACTCTACAAACTCGATAAACTCCGT	chr5:140529614-140529733
FBN2_18151	TTGGAGATTTCGATAGAGCGT	AAACTACCGACTACACCTCCG	chr5:127873440-127873551
HTATIP2_82369	TGTTAGGAGTTCGTTTTTAGGTTTC	GACTTAATCTTCCGAATAACCTCG	chr11:20385212-20385293
FBXO2_83919	CGTTTCGGTTCGTTAGGTTTC	TACGACGCGATAAAAATACGTC	chr1:11714840-11714926
ESR2_55656	TGAGTTGTAGGAGGTGCGTTC	AACACGTACTTTTCCCGCAT	chr14:64760791-64760881
TNFRSF10B_22599	TATAAATTAGTACGCGGTCGGAG	CCTTAAAATAAATCGAACATCGTC	chr8:22926454-22926534
DUSP6_18077	AATTTGTTTTAGTCGGTTCGTTT	ACGTCTCAATAAATACATTCTCCG	chr12:89746289-89746378
BCL2_17881	AATGAATTAGGAGTCGCGG	AAATCTCCGATTAAAATTCCTACG	chr18:60986532-60986645
CTGF_31220	TCGGAGCGTATAAAAGTTTCGG	GCACTAACTATCTCCTCTCAACGA	chr6:132272472-132272552
CXCR4_70043	CGGGAGTGTTAGAAAATGAATAAAC	GCGCCAAATAATAAACACGAA	chr2:136874129-136874228
EPHB2_62234	TAGTCGCGTTAGACGTTTTTC	AACAATAATCTCTCCCGACGC	chr1:23036966-23037079
DPP4_74913	TGGCGAAGAGGTTTTTATAGTTC	TACGCTCCTTCTCTAAACGCTC	chr2:162930431-162930534
DBC1_23879	AGGATAGGTATGAATTTCGGTTTC	AAACGAACGAACAACAACGA	chr9:122131521-122131614
TNFRSF10B_2	CGTAGTCGTTAGGGCGAAG	TAAATAAAAATCTCGTCAACGCA	chr8:22926654-22926750
EDNRB_1	CGTTTTAGTTAAGCGTGCGTG	CGAAATATAAAAATTCCCCTACGA	chr13:78493099-78493204
CRBP_2	TTTTAGCGCGTATAAAGTTTGC	GACCTAAACAAACCTAACCCTTCG	chr3:139258523-139258635
PENK_18533	GGGATCGTCGAGTAAAAGTTC	ATCTAAATCGCCTCCACGTCT	chr8:57358604-57358698
BCL2_17882	GATAGAGATTTATGTTTGTGTTCGC	TTTCAATCCGATATTCGCAA	chr18:60986696-60986793
KRAS_24235	AGGAGGGATTGTCGGATTTAC	GCTCCGAATCAAAATTAACGA	chr12:25404163-25404248

BioTrove assays

Rank	1	2	3	4	5	6	7	8	9	10	11	12	13	14	15	16	17
Assay	TNFRSF10D_23780	CXCR4_70046	FBN2_18150	NPY_18477	SOCS3_53602	FABP5_82270	MAL_74689	CDKN2B_27347	MYST4_82478	CYGB_83388	HPGD_82365	FAS_18143	TMEFF2_8419	DSC3_52537	VASH1_83272	CASP8M_1	NPTX2_57773
sens HR-DOD	64%	45%	36%	36%	36%	36%	36%	27%	27%	18%	18%	18%	18%	18%	18%	9%	9%
spec LR-SURV	100%	100%	100%	100%	100%	100%	100%	100%	100%	100%	100%	100%	100%	100%	100%	100%	100%

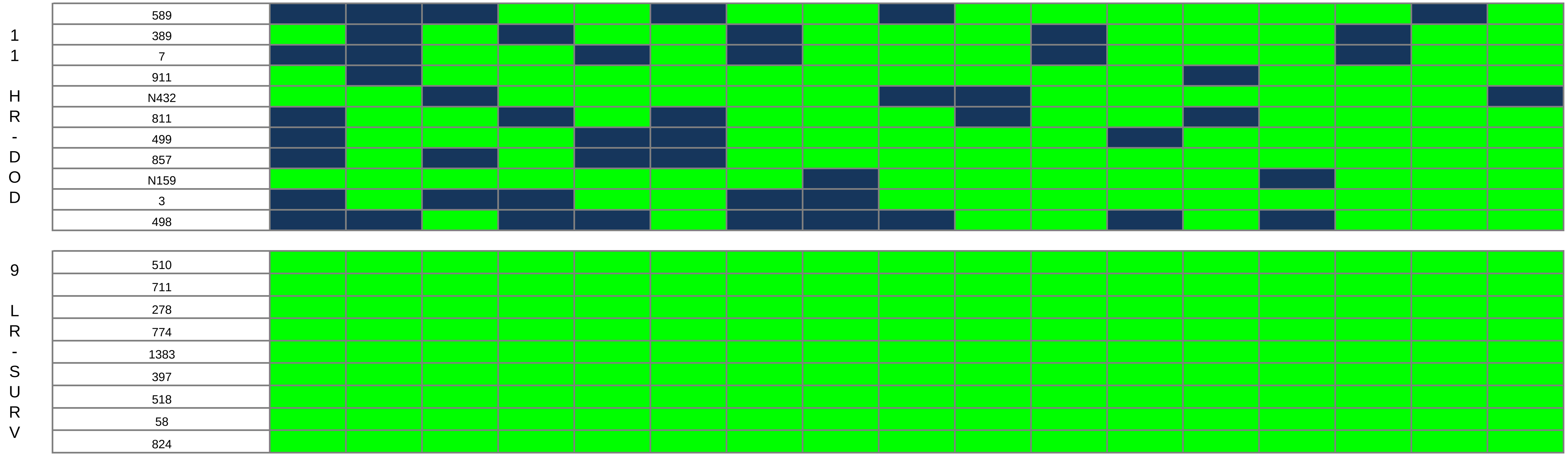

methylated
unmethylated

BioTrove results

Rank	18	19	20	21	22	23	24	25	26	27	28	29	30	31	32	33	34
Assay	PPIC_69739	PCDH10_82547	GATA4_2	CEPBD_GT_2	CASP8U_1	S100A6_83933	RARRES1_57581	CDKN2B_27345	KRT19_61596	RARRES1_57577	CSRP1_12560	TP73_72009	TADA3L_82757	TNFRSF10A_65529	NF2_23823	SLC4A11_82709	KRT19_83159
sens HR-DOD	9%	9%	9%	9%	9%	73%	55%	55%	45%	45%	36%	36%	36%	64%	64%	91%	55%
spec LR-SURV	100%	100%	100%	100%	100%	89%	86%	86%	83%	83%	80%	80%	80%	78%	78%	77%	75%

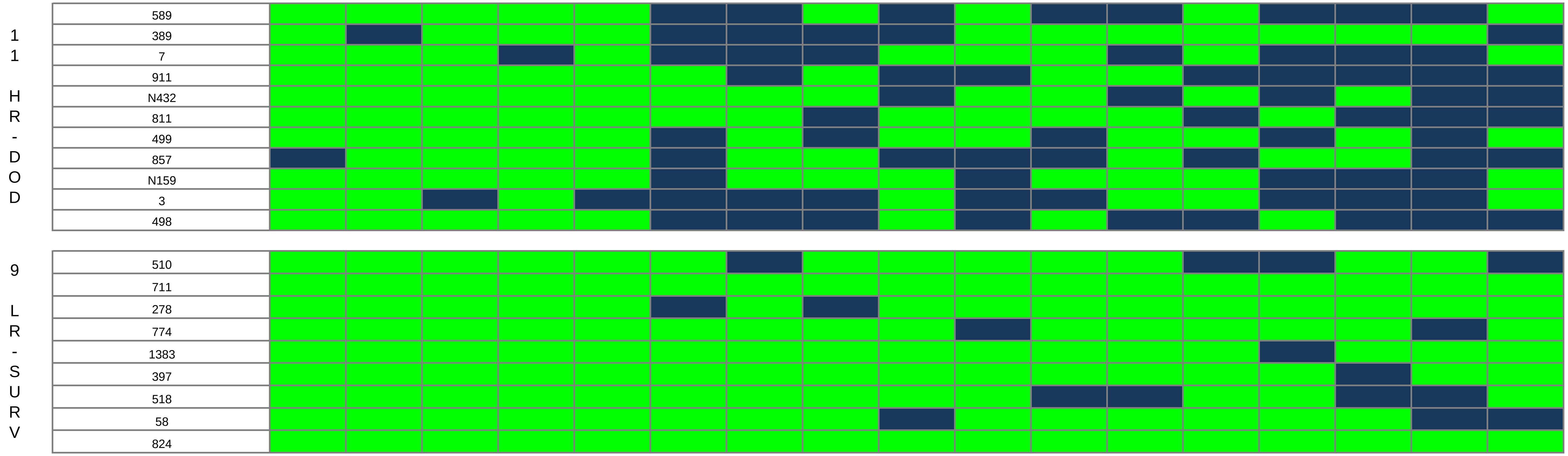

methylated
unmethylated

BioTrove results

Rank	35	36	37	38	39	40	41	42	43	44	45	46	47	48	49	50	51
Assay	NADK_83330	THBD_82800	NQO1_82535	NF2_61304	PCDHB6_82558	GATA4_13295	IGFBP1_82382	NAB2_83179	CDKN1C_83412	EGFR_23302	CTDSPL_23804	PDLIM4_4	RBP1_83281	CTCFL_23792	IGFBP3_54583	HPGD_82367	MAL_74692
sens HR-DOD	27%	27%	27%	27%	27%	73%	73%	73%	73%	73%	91%	45%	45%	45%	45%	64%	64%
spec LR-SURV	75%	75%	75%	75%	75%	73%	73%	73%	73%	73%	71%	71%	71%	71%	71%	70%	70%

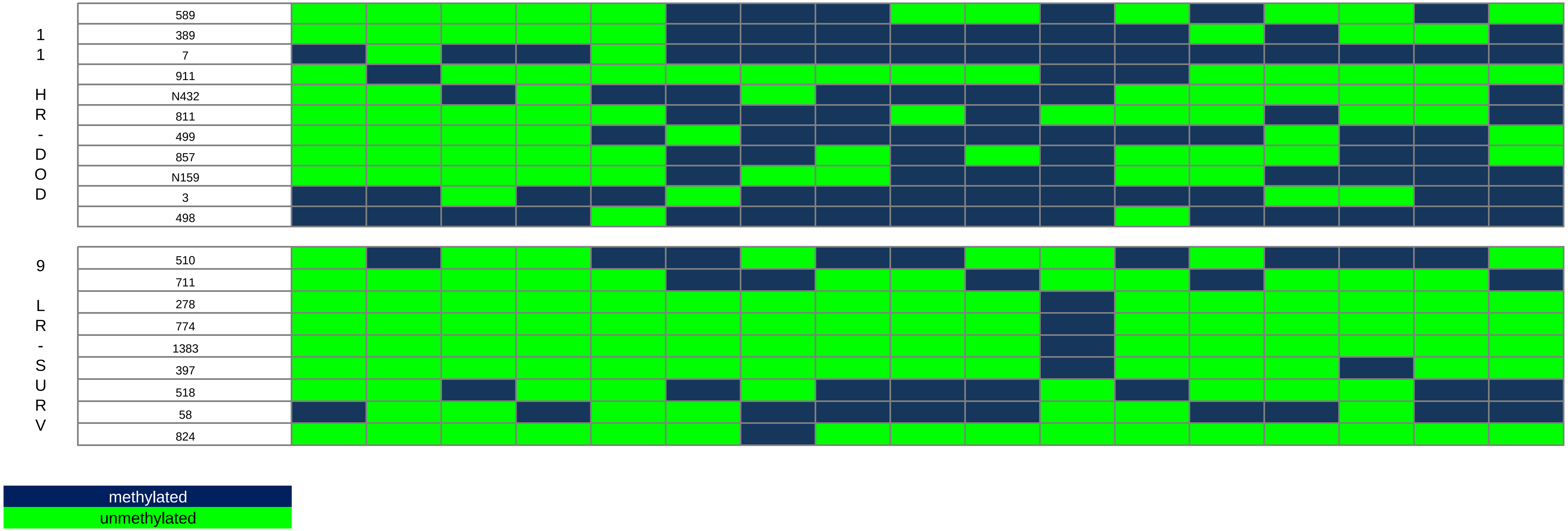

BioTrove results

Rank	52	53	54	55	56	57	58	59	60	61	62	63	64	65	66	67	68
Assay	EOMES_82239	DLC1_9164	PPIC_69743	EGFR_74891	INHA_13228	LATS2_82421	SLIT3_23619	CCNA1_1	TBX3_83233	F2R_74866	HOXA9_77983	TNFSF9_30093	F2R_31099	SOCS3_53605	NAB2_69824	ADAM23_66271	NPY_18473
sens HR-DOD	82%	82%	82%	91%	91%	73%	73%	73%	55%	55%	36%	36%	36%	36%	18%	18%	18%
spec LR-SURV	69%	69%	69%	67%	67%	67%	67%	67%	67%	67%	67%	67%	67%	67%	67%	67%	67%

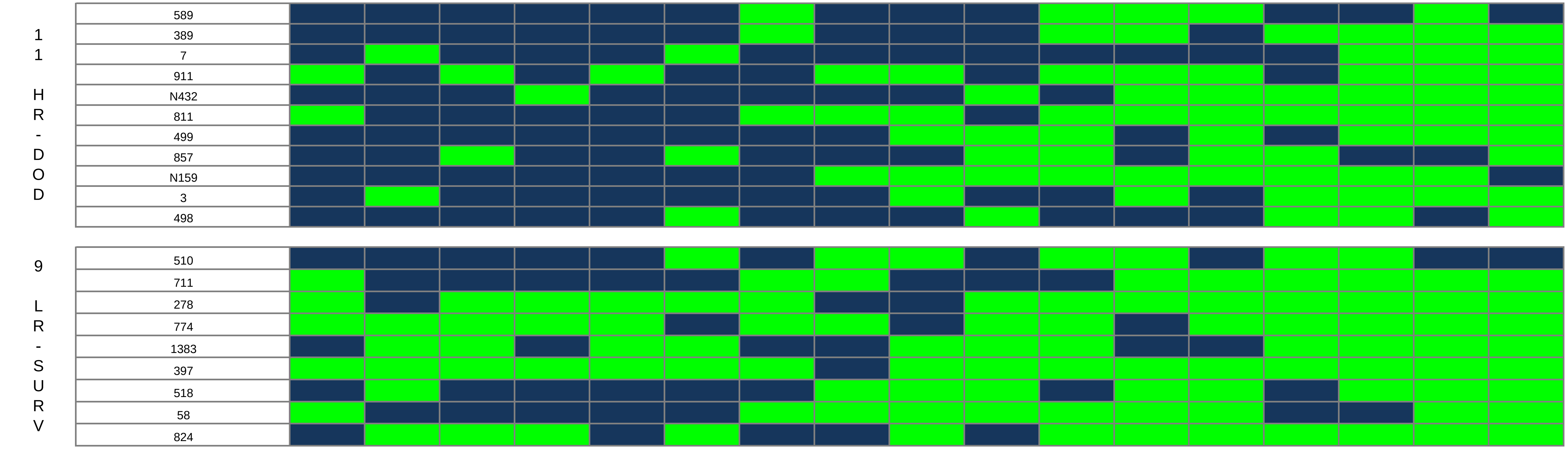

methylated
unmethylated

BioTrove results

Rank	69	70	71	72	73	74	75	76	77	78	79	80	81	82	83	84	85
Assay	SLC4A11_82719	CDKN1C_1	GREM1_29777	TNFRSF10B_22599	RPRM_1	SLC44A3_83941	TNFSF9_82827	EOMES_82240	DBC1_23888	EDNRB_3	PRTFDC1_82627	DSC3_52554	KRAS_82400	TNFRSF10C_9282	MRC2_82455	TCEB3_83953	ATM_9746
sens HR-DOD	18%	18%	18%	18%	100%	82%	82%	82%	82%	82%	64%	64%	64%	64%	64%	64%	64%
spec LR-SURV	67%	67%	67%	67%	65%	64%	64%	64%	64%	64%	64%	64%	64%	64%	64%	64%	64%

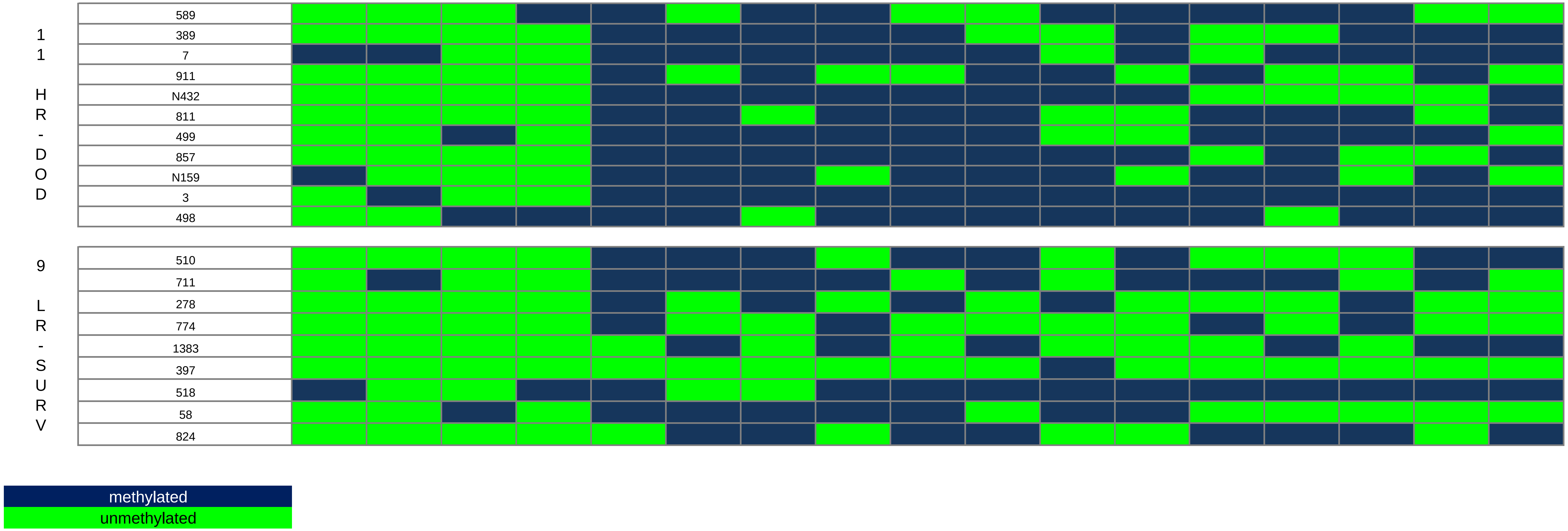

BioTrove results

Rank	86	87	88	89	90	91	92	93	94	95	96	97	98	99	100	101	102
Assay	NQO1_52551	DNAJC15_2	SPINT2_23336	ADAMTS5_82116	MYO18B_24620	DPP4_24687	TCEB3_83956	MORN1_83172	GREM1_29775	FOXE1_13317	HTATIP2_82372	SYCP3	TPM2_83253	TNFAIP2_83961	NNAT_82085	LATS2_82419	DIRAS3_9259
sens HR-DOD	91%	91%	91%	91%	91%	91%	45%	73%	73%	100%	100%	100%	100%	100%	100%	100%	82%
spec LR-SURV	63%	63%	63%	63%	63%	63%	63%	62%	62%	61%	61%	61%	61%	61%	61%	61%	60%

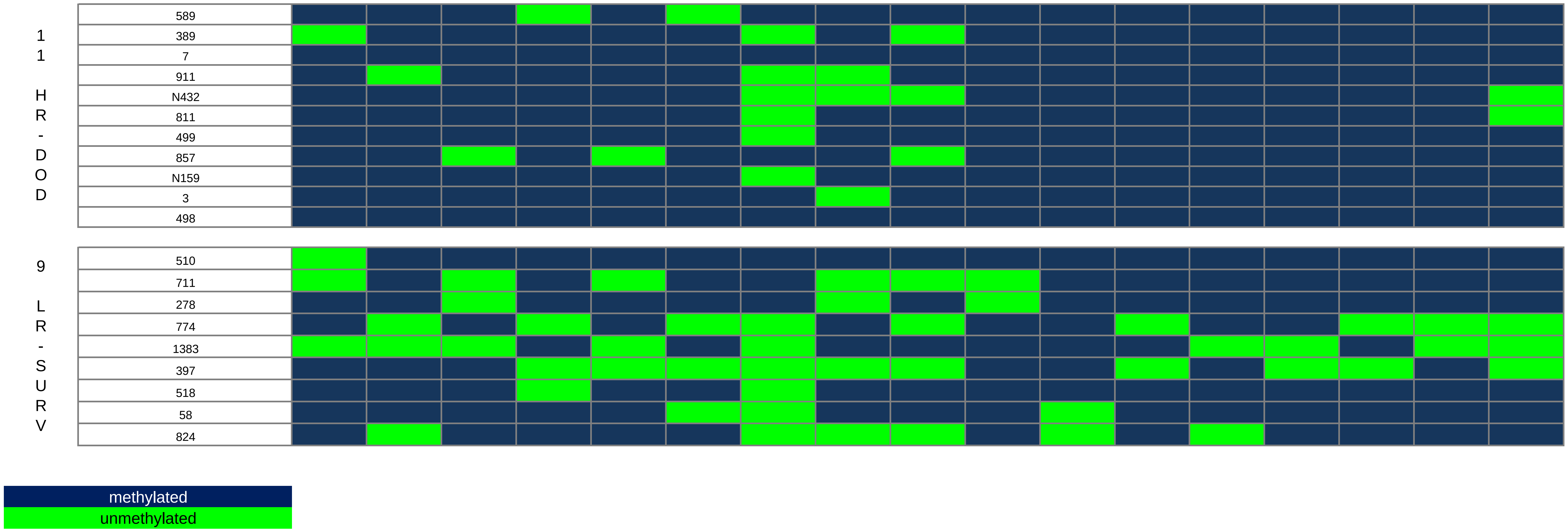

BioTrove results

Rank	103	104	105	106	107	108	109	110	111	112	113	114	115	116	117	118	119
Assay	ZIM2_82923	RAMP3_82633	GPR50_82319	PCSK6_56483	CAV1_58524	PCDH20_23327	MRC2_9206	ESR2_74878	RASSF1A	TNFRSF10D_23781	DUSP6_18075	IGFBP3_RadRes	CEPBD_GT_1	PDLIM4_60076	NNAT_83189	PCDH20_23326	DNAJC15_1
sens HR-DOD	82%	82%	82%	55%	55%	27%	27%	27%	91%	91%	91%	91%	91%	91%	91%	64%	100%
spec LR-SURV	60%	60%	60%	60%	60%	60%	60%	60%	59%	59%	59%	59%	59%	59%	59%	58%	58%

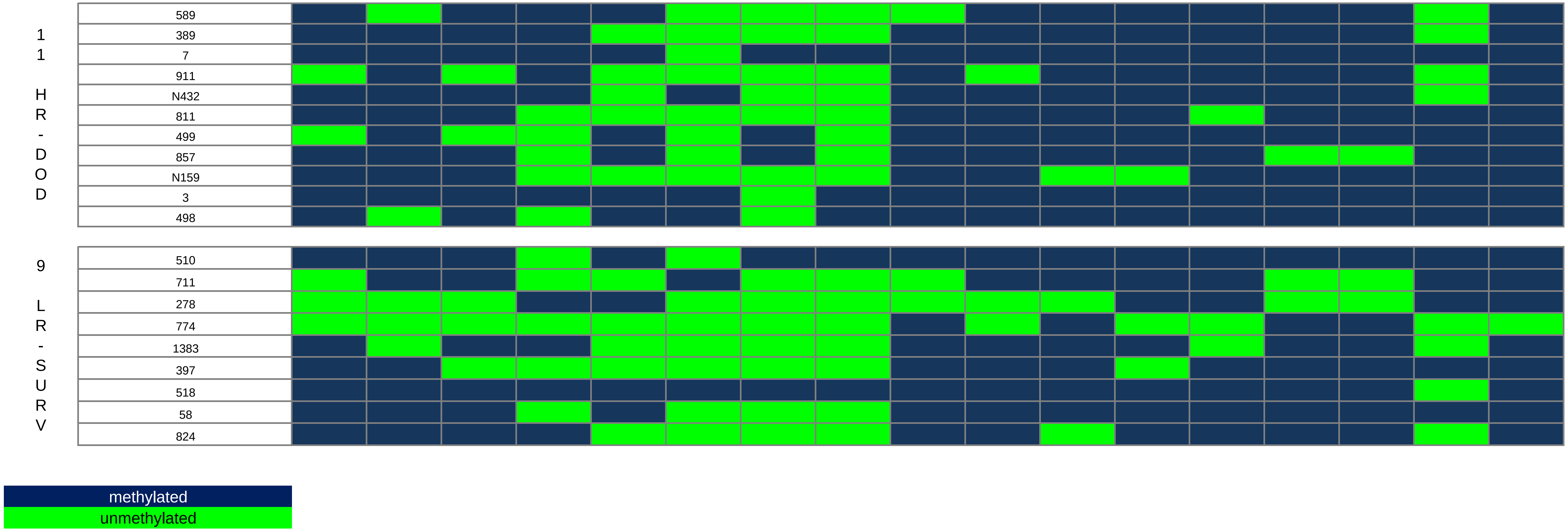

BioTrove results

Rank	120	121	122	123	124	125	126	127	128	129	130	131	132	133	134	135	136
Assay	DMRTB1_12825	PENK_18538	ID4_56952	RARB_60785	DMRTB1_83381	CCNA1_83097	CSRP1_12558	ESR1_2	TPM2_82850	IGFBP1_82385	CTGF_31218	TMEFF2_8421	HOXA9_2	TP73_1	PCSK6_56480	WNT5A_83273	PRDX2_82604
sens HR-DOD	100%	100%	100%	100%	100%	100%	100%	100%	73%	73%	73%	73%	73%	36%	36%	36%	82%
spec LR-SURV	58%	58%	58%	58%	58%	58%	58%	58%	57%	57%	57%	57%	57%	57%	57%	57%	56%

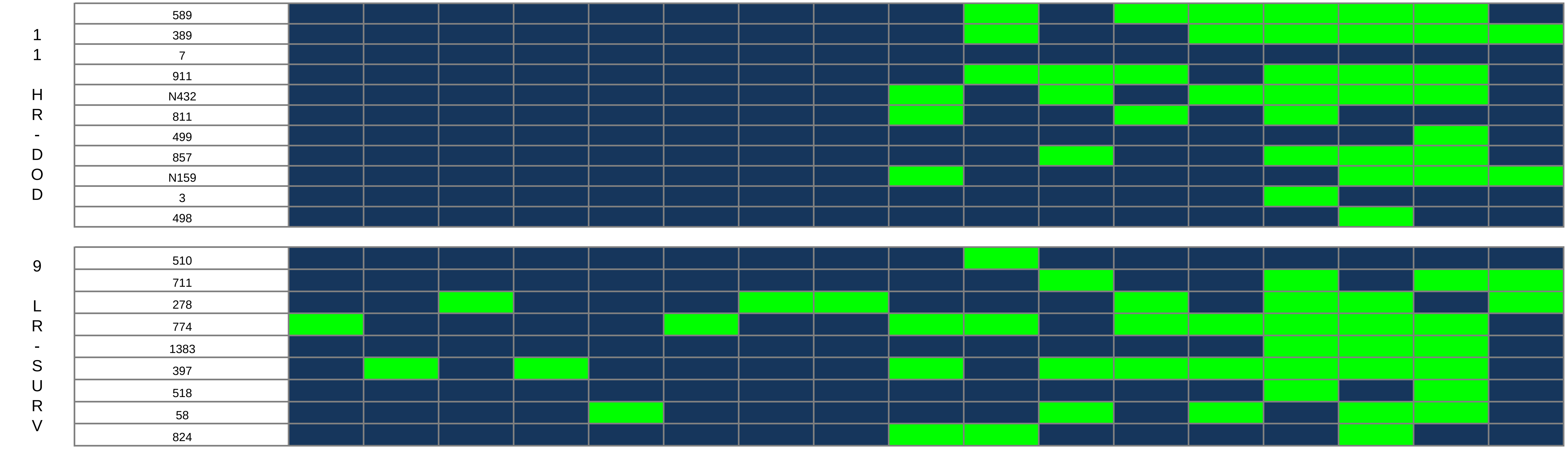

methylated
unmethylated

BioTrove results

Rank	137	138	139	140	141	142	143	144	145	146	147	148	149	150	151	152	153
Assay	EMP3_82232	CTCFL_23794	TNFRSF10C_9283	TBX3_82767	EPHB2_54970	NADK_83183	ROBO3_64828	RBP4_24108	SLC25A31_82698	HLA-B_82358	RARB_60784	ADAM23_A	CRBP_1	SPINT2_23333	CYP1A1_23831	WNT5A_65596	SLC25A31_82696
sens HR-DOD	82%	82%	82%	82%	91%	91%	45%	45%	100%	100%	100%	100%	100%	100%	100%	100%	100%
spec LR-SURV	56%	56%	56%	56%	56%	56%	56%	56%	55%	55%	55%	55%	55%	55%	55%	55%	55%

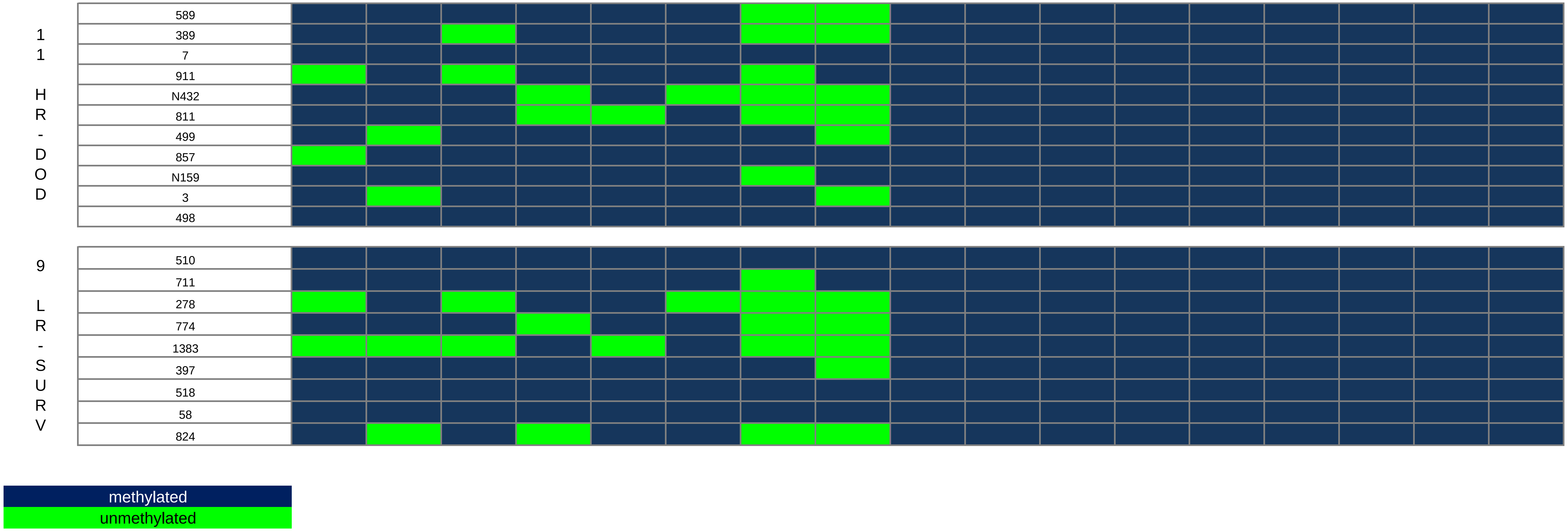

BioTrove results

Rank		154	155	156	157	158	159	160	161	162	163	164	165	166	167	168	169	170
Assay		ZBTB25_83276	MORN1_83171	TSHR_83270	TEX14_56176	TRIM59_82871	DAZL_18008	ID4_56956	SST_83224	DAZL_22631	AHR_82118	FBXO2_83916	FAS_18144	MAGEB2_71596	MSH4_27679	TADA3L_82759	DIRAS3_9258	SLIT3_23622
sens HR-DOD		100%	100%	100%	100%	100%	100%	55%	55%	64%	64%	73%	73%	91%	91%	91%	82%	82%
spec LR-SURV		55%	55%	55%	55%	55%	55%	55%	55%	54%	54%	53%	53%	53%	53%	53%	50%	50%
11 HR-DOD	589	methylated	methylated	methylated	methylated	methylated	methylated	methylated	unmethylated	unmethylated	methylated	methylated	methylated	methylated	methylated	methylated	methylated	methylated
	389	methylated	methylated	methylated	methylated	methylated	methylated	unmethylated	methylated	methylated	unmethylated	methylated	methylated	methylated	methylated	methylated	methylated	methylated
	7	methylated	methylated	methylated	methylated	methylated	methylated	unmethylated	methylated	methylated	methylated	unmethylated	methylated	methylated	methylated	methylated	methylated	methylated
	911	methylated	methylated	methylated	methylated	methylated	methylated	unmethylated	unmethylated	methylated	unmethylated	methylated	unmethylated	methylated	methylated	methylated	unmethylated	methylated
	N432	methylated	methylated	methylated	methylated	methylated	methylated	methylated	methylated	unmethylated	methylated	methylated	methylated	methylated	unmethylated	methylated	unmethylated	unmethylated
	811	methylated	methylated	methylated	methylated	methylated	methylated	methylated	unmethylated	unmethylated	methylated	unmethylated	unmethylated	methylated	methylated	methylated	methylated	methylated
	499	methylated	methylated	methylated	methylated	methylated	methylated	methylated	unmethylated	methylated	unmethylated	unmethylated	methylated	methylated	methylated	methylated	methylated	methylated
	857	methylated	methylated	methylated	methylated	methylated	methylated	unmethylated	unmethylated	methylated	methylated	methylated	methylated	unmethylated	methylated	methylated	methylated	unmethylated
	N159	methylated	methylated	methylated	methylated	methylated	methylated	methylated	methylated	unmethylated	unmethylated	methylated	unmethylated	methylated	methylated	methylated	methylated	methylated
	3	methylated	methylated	methylated	methylated	methylated	methylated	unmethylated	methylated	methylated	methylated	methylated	methylated	methylated	methylated	unmethylated	methylated	methylated
	498	methylated	methylated	methylated	methylated	methylated	methylated	methylated	methylated	methylated	methylated	methylated	methylated	methylated	methylated	methylated	methylated	methylated
9 LR-SURV	510	methylated	methylated	methylated	methylated	methylated	methylated	unmethylated	methylated	methylated	methylated	methylated	methylated	methylated	methylated	methylated	methylated	methylated
	711	methylated	methylated	methylated	methylated	methylated	methylated	methylated	methylated	methylated	methylated	methylated	methylated	methylated	methylated	methylated	methylated	methylated
	278	methylated	methylated	methylated	methylated	methylated	methylated	unmethylated	unmethylated	unmethylated	unmethylated	methylated	unmethylated	methylated	methylated	methylated	methylated	methylated
	774	methylated	methylated	methylated	methylated	methylated	methylated	unmethylated	unmethylated	methylated	unmethylated	methylated	methylated	methylated	methylated	methylated	methylated	methylated
	1383	methylated	methylated	methylated	methylated	methylated	methylated	methylated	unmethylated	unmethylated	methylated	unmethylated	methylated	methylated	methylated	methylated	methylated	methylated
	397	methylated	methylated	methylated	methylated	methylated	methylated	unmethylated	unmethylated	unmethylated	unmethylated	methylated	unmethylated	methylated	methylated	methylated	methylated	methylated
	518	methylated	methylated	methylated	methylated	methylated	methylated	methylated	methylated	methylated	methylated	methylated	methylated	methylated	methylated	methylated	methylated	methylated
	58	methylated	methylated	methylated	methylated	methylated	methylated	methylated	methylated	methylated	methylated	methylated	methylated	methylated	methylated	methylated	methylated	methylated
	824	methylated	methylated	methylated	methylated	methylated	methylated	methylated	methylated	methylated	methylated	unmethylated	methylated	methylated	methylated	methylated	methylated	methylated

methylated
unmethylated

BioTrove results

Rank	171	172	173	174	175	176	177	178	179	180	181	182	183	184	185	186	187
Assay	ZIM2_82925	HCRTR2_82341	CYGB_17997	SST_23808	HCRTR2_82338	CTDSPL_23803	THBD_82802	MYST4_83331	FABP3_83914	RBP4_24106	FOXE1_13314	TNFRSF10A_9277	RUNX3_2	XYLB_65485	FABP5_82271	XYLB_65481	FBXO2_83919
sens HR-DOD	64%	55%	55%	45%	45%	36%	27%	27%	27%	18%	18%	9%	9%	9%	9%	9%	9%
spec LR-SURV	50%	50%	50%	50%	50%	50%	50%	50%	50%	50%	50%	50%	50%	50%	50%	50%	50%

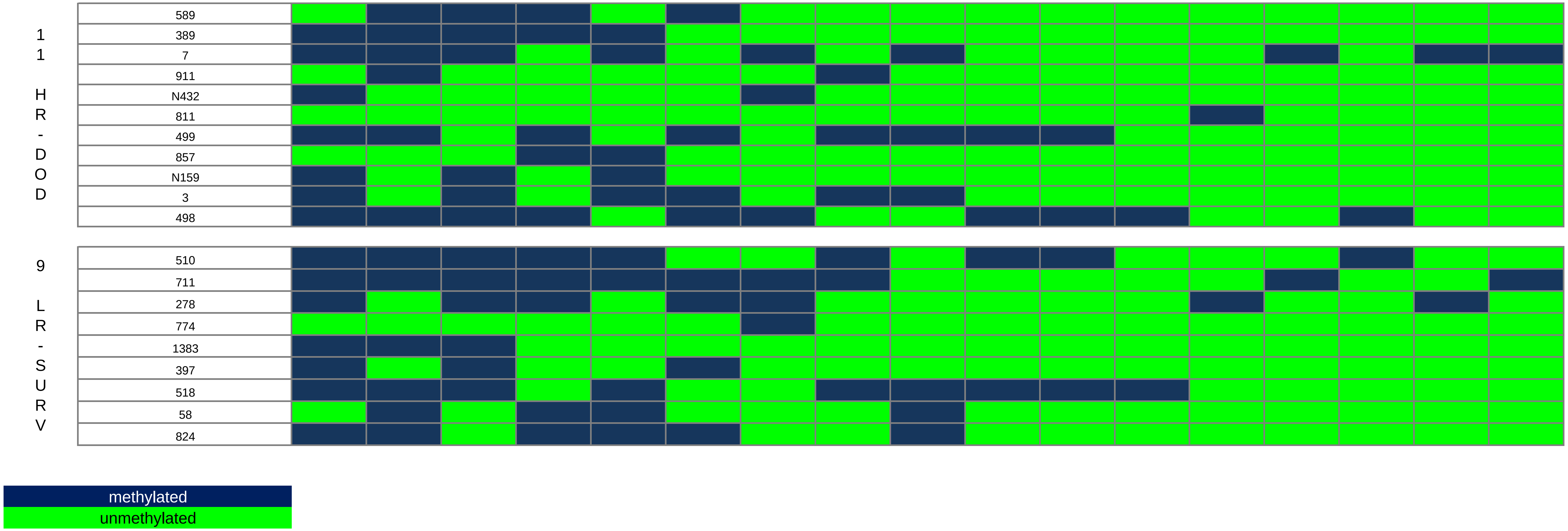

BioTrove results

Rank	188	189	190	191	192	193	194	195	196	197	198	199	200	201	202	203	204
Assay	DUSP6_18077	CTGF_31220	DPP4_74913	TNFRSF10B_2	PCDH10_83191	DLC1_83387	FABP3_83912	FBN2_18151	SLC44A3_83942	NPTX2_57779	ESR2_55656	PRDX2_83202	EPHB2_62234	EDNRB_1	DBC1_23879	CXCR4_70043	GREM1_83135
sens HR-DOD	9%	9%	9%	9%	73%	64%	55%	55%	27%	18%	18%	9%	9%	9%	27%	0%	0%
spec LR-SURV	50%	50%	50%	50%	47%	47%	46%	46%	43%	40%	33%	33%	33%	33%	30%	0%	

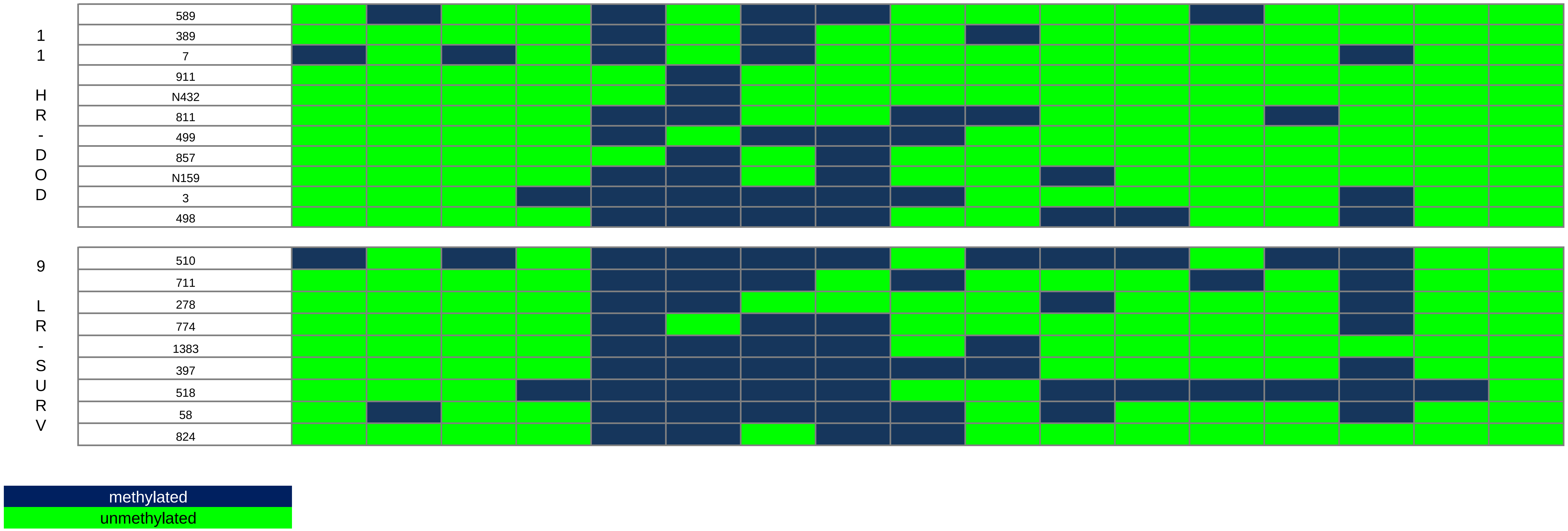

BioTrove results

Rank	205	206	207	208	209	210	211	212
Assay	TRIM59_82865	S100A6_83934	HTATIP2_82369	BCL2_17881	CRBP_2	PENK_18533	BCL2_17882	KRAS_24235
sens HR-DOD	0%	0%	0%	0%	0%	0%	0%	0%
spec LR-SURV								

Group	Sample	205	206	207	208	209	210	211	212
11 HR-DOD	589								
	389								
	7								
	911								
	N432								
	811								
	499								
	857								
	N159								
	3								
	498								
9 LR-SURV	510								
	711								
	278								
	774								
	1383								
	397								
	518								
	58								
	824								

methylated
unmethylated

BioTrove results
