# Supplementary material for: Genome-wide promoter methylation analysis in neuroblastoma identifies prognostic methylation biomarkers
Source: Genome Biol. 2012 Oct 3;13(10):R95. doi: 10.1186/gb-2012-13-10-r95 (PMC3491423; doi:10.1186/gb-2012-13-10-r95)
Supplement: Additional file 2 — Visualization of the protocadherin beta gene cluster and the HIST1H3C promoter region in the Integrative Genomic Viewer. Eight neuroblastoma cell lines (SK-N-AS, CLB-GA, SH-SY5Y, SJNB-1, CHP-902R, IMR-32, LAN-2 and N206) and the MBD-seq results are displayed. [file gb-2012-13-10-r95-S2.pdf]

Figure 1: Visualization of a part of the protocadherin β (*PCDHB*) family cluster in the Integrative Genomics Viewer (IGV). For each neuroblastoma cell line (SK-N-AS, CLB-GA, SH-SY5Y, SJNB-1, CHP-902R, IMR-32, LAN-2 and N206), the number of sequencing tags at each position is shown and the location of detected peaks is indicated with a red bar. The captured sequences clearly overlap with CpG islands in each individual member of this gene cluster.

Figure 2: Visualization of the promoter region of *HIST1H3C* in the Integrative Genomics Viewer (IGV). The four neuroblastoma cell lines at the bottom (CHP-902R, IMR-32, LAN-2 and N206) are *MYCN* amplified. Three of these cell lines clearly show sequence tags in the CpG island, while the four neuroblastoma cell lines on top (SK-N-AS, CLB-GA, SH-SY5Y and SJNB-1), which are *MYCN* single copy cell lines, do not show any signal.

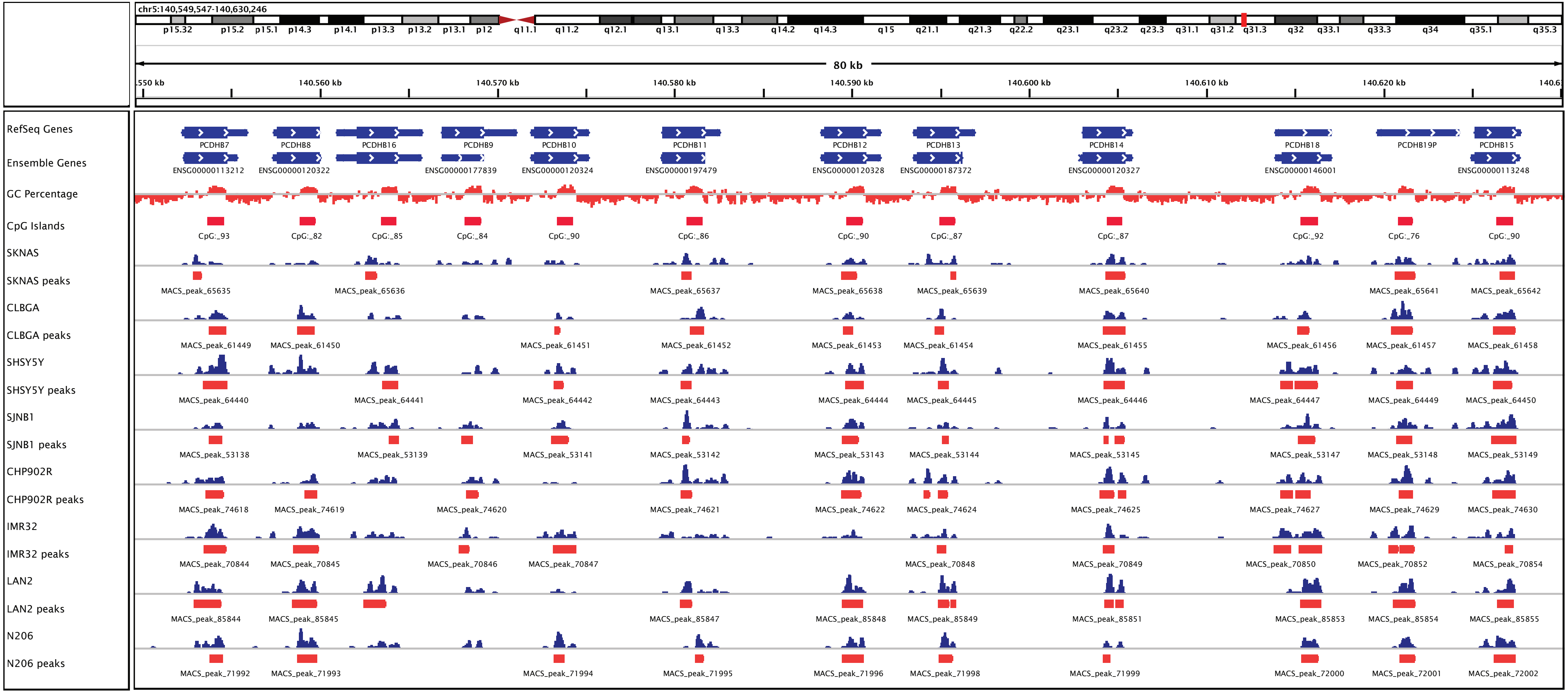
chr5:140,549,547-140,630,246
p15.32 p15.2 p15.1 p14.3 p14.1 p13.3 p13.2 p13.1 p12 q11.1 q11.2 q12.1 q13.1 q13.3 q14.2 q14.3 q15 q21.1 q21.3 q22.2 q23.1 q23.2 q23.3 q31.1 q31.2 q31.3 q32 q33.1 q33.3 q34 q35.1 q35.3
80 kb
550 kb 140.560 kb 140.570 kb 140.580 kb 140.590 kb 140.600 kb 140.610 kb 140.620 kb
RefSeq Genes
PCDHB7 PCDHB8 PCDHB16 PCDHB9 PCDHB10 PCDHB11 PCDHB12 PCDHB13 PCDHB14 PCDHB18 PCDHB19P PCDHB15
Ensemble Genes
ENSG00000113212 ENSG00000120322 ENSG00000177839 ENSG00000120324 ENSG00000197479 ENSG00000120328 ENSG00000187372 ENSG00000120327 ENSG00000146001 ENSG00000113248
GC Percentage
CpG Islands
CpG:_93 CpG:_82 CpG:_85 CpG:_84 CpG:_90 CpG:_86 CpG:_90 CpG:_87 CpG:_87 CpG:_92 CpG:_76 CpG:_90
SKNAS
SKNAS peaks
MACS_peak_65635 MACS_peak_65636 MACS_peak_65637 MACS_peak_65638 MACS_peak_65639 MACS_peak_65640 MACS_peak_65641 MACS_peak_65642
CLBGA
CLBGA peaks
MACS_peak_61449 MACS_peak_61450 MACS_peak_61451 MACS_peak_61452 MACS_peak_61453 MACS_peak_61454 MACS_peak_61455 MACS_peak_61456 MACS_peak_61457 MACS_peak_61458
SHSY5Y
SHSY5Y peaks
MACS_peak_64440 MACS_peak_64441 MACS_peak_64442 MACS_peak_64443 MACS_peak_64444 MACS_peak_64445 MACS_peak_64446 MACS_peak_64447 MACS_peak_64449 MACS_peak_64450
SJNB1
SJNB1 peaks
MACS_peak_53138 MACS_peak_53139 MACS_peak_53141 MACS_peak_53142 MACS_peak_53143 MACS_peak_53144 MACS_peak_53145 MACS_peak_53147 MACS_peak_53148 MACS_peak_53149
CHP902R
CHP902R peaks
MACS_peak_74618 MACS_peak_74619 MACS_peak_74620 MACS_peak_74621 MACS_peak_74622 MACS_peak_74624 MACS_peak_74625 MACS_peak_74627 MACS_peak_74629 MACS_peak_74630
IMR32
IMR32 peaks
MACS_peak_70844 MACS_peak_70845 MACS_peak_70846 MACS_peak_70847 MACS_peak_70848 MACS_peak_70849 MACS_peak_70850 MACS_peak_70852 MACS_peak_70854
LAN2
LAN2 peaks
MACS_peak_85844 MACS_peak_85845 MACS_peak_85847 MACS_peak_85848 MACS_peak_85849 MACS_peak_85851 MACS_peak_85853 MACS_peak_85854 MACS_peak_85855
N206
N206 peaks
MACS_peak_71992 MACS_peak_71993 MACS_peak_71994 MACS_peak_71995 MACS_peak_71996 MACS_peak_71998 MACS_peak_71999 MACS_peak_72000 MACS_peak_72001 MACS_peak_72002

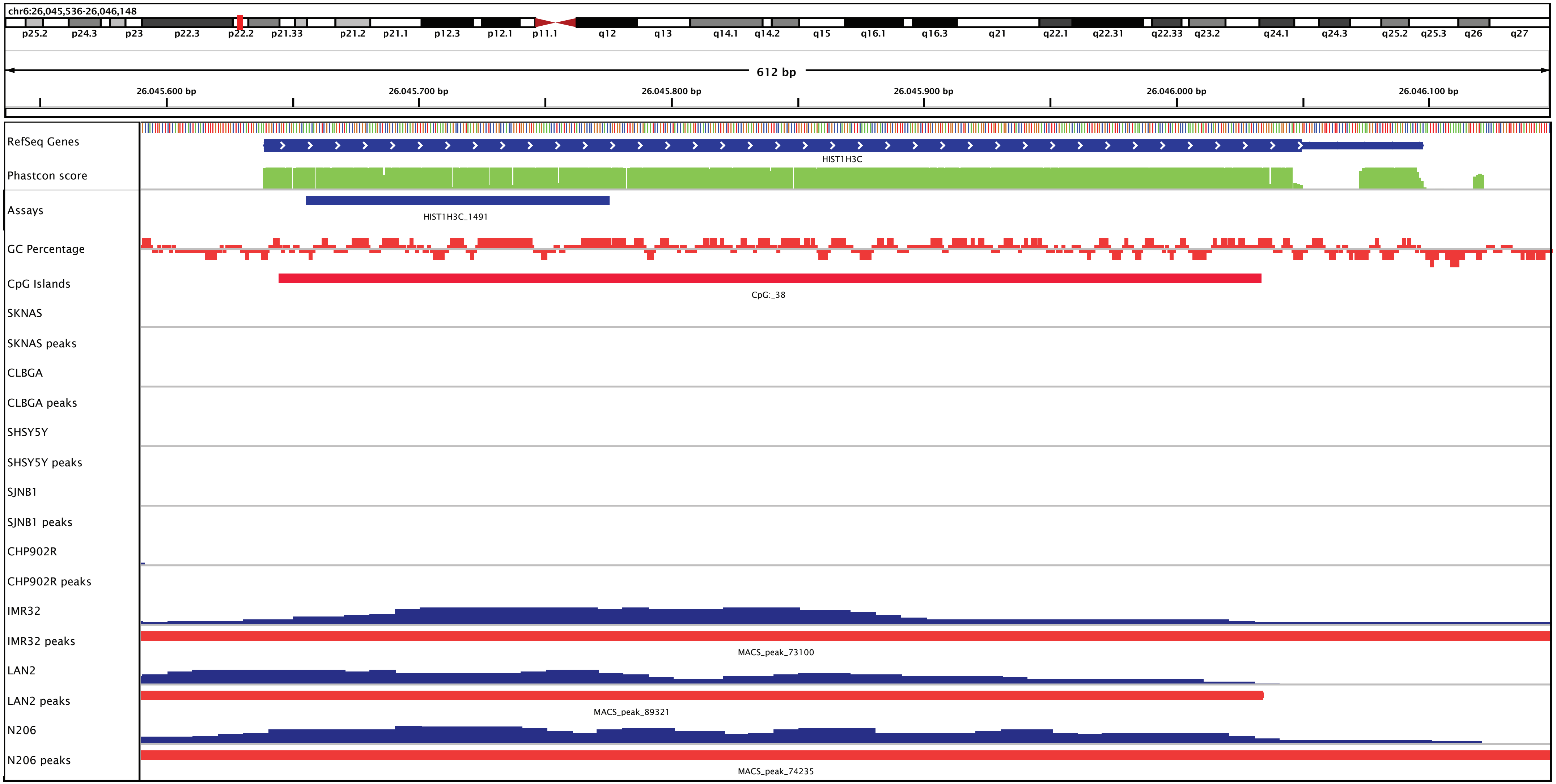
chr6:26,045,536-26,046,148
p25.2
p24.3
p23
p22.3
p22.2
p21.33
p21.2
p21.1
p12.3
p12.1
p11.1
q12
q13
q14.1
q14.2
q15
q16.1
q16.3
q21
q22.1
q22.31
q22.33
q23.2
q24.1
q24.3
q25.2
q25.3
q26
q27
612 bp
26.045.600 bp
26.045.700 bp
26.045.800 bp
26.045.900 bp
26.046.000 bp
26.046.100 bp
RefSeq Genes
HIST1H3C
Phastcon score
Assays
HIST1H3C_1491
GC Percentage
CpG Islands
CpG:_38
SKNAS
SKNAS peaks
CLBGA
CLBGA peaks
SHSY5Y
SHSY5Y peaks
SJNB1
SJNB1 peaks
CHP902R
CHP902R peaks
IMR32
IMR32 peaks
MACS_peak_73100
LAN2
LAN2 peaks
MACS_peak_89321
N206
N206 peaks
MACS_peak_74235
