# Supplementary material for: Genome-wide promoter methylation analysis in neuroblastoma identifies prognostic methylation biomarkers
Source: Genome Biol. 2012 Oct 3;13(10):R95. doi: 10.1186/gb-2012-13-10-r95 (PMC3491423; doi:10.1186/gb-2012-13-10-r95)
Supplement: Additional file 3 — Clinical patient annotation, summary of clinical characteristics, MSP assays, results and summarized results (per clinical parameter) on the LC480 platform for 89 NB patient samples. Assays differentially methylated between prognostic groups and between neuroblastoma risk factors are discussed in detail, as well as extended analyses on the MSP data (hierarchical clustering (heatmap) and survival analysis according to the number of methylation events (Kaplan-Meier plot)). [file gb-2012-13-10-r95-S3.pdf]

Part LC480 clinical annotation: Patient characteristics of the samples used for the high-throughput MSP screening on the Roche LC480. PatientID is a unique patient number, the group indicates the risk. Clinical characteristics given are the age at diagnosis in months, International Neuroblastoma Staging System (INSS) stage, *MYCN* status (0 is non-amplified/single copy and 1 is amplified), follow-up time (FU) in days after diagnosis, and overall survival (OS) and event-free survival (EFS) time in days after diagnosis. OS indicates whether the patient was alive (0) at the last known FU or died of disease (1). Similar for EFS, indicating events such as relapse or progression. Segmental aberrations for chromosome 1p, 11q and 17q are indicated with normal, partial loss (PL), whole loss (WL), partial gain (PG) or whole gain (WG). Empty cells represent missing values.

Part LC480 overview annotation: Summary of the clinical characteristics of the 89 primary neuroblastoma samples used in the high-throughput MSP screening. HR-DOD: high-risk deceased patients, HR-SURV: high-risk patients alive for at least 1000 days follow-up, LR-SURV: low-risk patients alive for at least 1000 days follow-up, and INSS: International Neuroblastoma Staging System.

Part LC480 assays: MSP assays used in the high-throughput MSP screening on the Roche LC480. For each tested gene, the assay name(s) and corresponding forward and reverse primer(s) (5’ to 3’) are indicated, as well as the genomic location of the amplicon on the hg19 reference genome.

Part LC480 results: Results of the high-throughput MSP screening on the Roche LC480. For each assay and for each sample, the methylation call (dark blue is methylated, green

unmethylated) is given. The patient samples are subdivided into three prognostic groups (LR-SURV, HR-DOD and HR-SURV). HR-DOD: high-risk deceased patients, HR-SURV: high-risk patients alive for at least 1000 days follow-up, LR-SURV: low-risk patients alive for at least 1000 days follow-up, CL: cell line, Neg: negative control (HCT-116 DKO cell line), and NTC: no template control.

Part LC480 overview results: Summary of the MSP results of the high-throughput study. The number of methylated samples for a particular MSP assay within each prognostic group and for the entire sample set is given (percentage of methylated samples between brackets). Assays are ranked descending on the number of overall methylated samples. Assays discussed in more detail in the results section are indicated in grey. HR-DOD: high-risk deceased patients, HR-SURV: high-risk patients alive for at least 1000 days follow-up, LR-SURV: low-risk patients alive for at least 1000 days follow-up.

Part Group associations: Detailed information on a selection of assays, differentially methylated between prognostic groups (per prognostic group and per combination of risk factors). The number (percentage) of methylated samples in each stratum is given. HR-DOD: high-risk deceased patients, HR-SURV: high-risk patients alive for at least 1000 days follow-up, LR-SURV: low-risk patients alive for at least 1000 days follow-up.

Part Risk factor associations: Detailed information on a selection of assays, differentially methylated between neuroblastoma risk factors (per (combination of) risk factors). The number (percentage) of methylated samples in each stratum is given. HR-DOD:

high-risk deceased patients, HR-SURV: high-risk patients alive for at least 1000 days follow-up, LR-SURV: low-risk patients alive for at least 1000 days follow-up.

Part Heatmap: Hierarchical clustering based on the MSP data of the 89 primary neuroblastoma tumor samples. This analysis reveals two clusters, indicated with group 1 and group 2, which predominantly distinguish high-risk and low-risk neuroblastoma patient samples. Each sample is assigned to a prognostic group (LR-SURV, HR-DOD and HR-SURV). HR-DOD: high-risk deceased patients, HR-SURV: high-risk patients alive for at least 1000 days follow-up and LR-SURV: low-risk patients alive for at least 1000 days follow-up.

Part KM number: Kaplan-Meier plot: overall survival in the samples of the high-throughput MSP screening according to the number of methylation events. The purple line indicates patients with 0 to 16 methylation events, the red line patients with 16 to 27 methylation events. This 16 methylation events cut-off was used, as the average number of methylation events in one sample is 16. The p-value is determined using a log-rank test (Mantel-Cox). Time is indicated in days, starting from diagnosis.

PatientID	Group	Age at diagnosis (months)	INSS Stage	*MYCN*	Chr1p	Chr17q	Chr11q	FU time (days)	OS time (days)	EFS time (days)	OS	EFS
5	HR-DOD	146	4	1	PL		WL	754	754	281	1	1
41	HR-DOD	51	4	0	normal		WL	569	569	397	1	1
384	HR-DOD	28	4	0	normal		WL	966	966	890	1	1
513	HR-DOD	30	4	1	PL		normal	167	167	142	1	1
610	HR-DOD	35	4	1	normal		WL	850	850	782	1	1
906	HR-DOD	19	3	1	PL	PG	normal	216	216	53	1	1
917	HR-DOD	92	4	1	PL	PG	normal	485	485	439	1	1
928	HR-DOD	12	4	1	PL	PG	normal	412	412	286	1	1
1015	HR-DOD	42	4	0	normal		WL	581	581	449	1	1
1378	HR-DOD	155	4	0	normal		WL	4910	4910	3793	1	1
1467	HR-DOD	0	4	1	normal	PG	normal	1	1	1	1	1
1473	HR-DOD	8	4	1	PL	PG	normal	239	239	116	1	1
1477	HR-DOD	79	4	1	PL	PG	normal	1246	1246	898	1	1
1507	HR-DOD	14	4	1				285	285	273	1	1
1517	HR-DOD	35	4	0	normal	PG	PL	547	547	433	1	1
1520	HR-DOD	40	4	1	normal	PG	PL	1279	1279	552	1	1
1522	HR-DOD	23	3	1	PL	PG	normal	728	728	594	1	1
1527	HR-DOD	108	4	1	PL	normal	normal	319	319		1	1
1648	HR-DOD	53	4	0	normal	PG	PL	1221	1221	341	1	1
1781	HR-DOD	41	4	1				679	679	651	1	1
2859	HR-DOD	63	3	1				4493	4493	3861	1	1
2865	HR-DOD	91	4	0				1353	1353	1353	1	1
2866	HR-DOD	80	4	0				1300	1300	786	1	1
2869	HR-DOD	72	4	0				1380	1380	1011	1	1
E061	HR-DOD	31	4	0	normal	PG	PL	1445	1445	497	1	1
E069	HR-DOD	59	4	0	normal	PG	PL	2836	2836	2016	1	1
E282	HR-DOD	17	4	1	PL	PG	normal	711	711	433	1	1
E290	HR-DOD	6	4	1	PL	normal	normal	539	539	531	1	1
526	HR-SURV	26	4	1	normal	PG	normal	2009	2009		0	0
915	HR-SURV	16	4	1	PL		normal	1985	1985		0	0
1017	HR-SURV	22	3	1	PL	PG	normal	1758	1758		0	0
1148	HR-SURV	27	3	1				1212	1212		0	0
1384	HR-SURV	16	3	1				1620	1620		0	0
1385	HR-SURV	31	3	1				1185	1185		0	0
1431	HR-SURV	23	4	1	PL	PG	normal	953	953		0	0
1501	HR-SURV	131	4	0				1558	1558	1558	0	1
1508	HR-SURV	6	2	1				1449	1449		0	0
1521	HR-SURV	38	4	1	normal	WG	WL	2163	2163		0	0
1524	HR-SURV	23	3	1	PL	PG	WL	2387	2387		0	0
1647	HR-SURV	10	2	1				2136	2136		0	0
1789	HR-SURV	25	3	1				5616	5616		0	0
1793	HR-SURV	33	3	1				1176	1176		0	0
1794	HR-SURV	33	3	1				5096	5096		0	0
1800	HR-SURV	71	4	1				1862	1862		0	0
2855	HR-SURV	32	3	1				1386	1386		0	0
2856	HR-SURV	41	3	1				1330	1330	834	0	1
2857	HR-SURV	29	3	1				1295	1295		0	0
2860	HR-SURV	66	3	1				1489	1489		0	0
2861	HR-SURV	19	3	1				1514	1514	445	0	1
2862	HR-SURV	43	2	1				1161	1161		0	0
2863	HR-SURV	11	4	1				1237	1237		0	0
2864	HR-SURV	26	4	0				1310	1310		0	0
2867	HR-SURV	46	4	0				1112	1112		0	0
E458	HR-SURV	7	4	0	normal	PG	PL	2284	2284	1399	0	1
E579	HR-SURV	13	4	0	normal	PG	PL	3534	3534		0	0
E598	HR-SURV	49	4	0	PL	PG	PL	3219	3219		0	0
E685	HR-SURV	14	4	0	normal	PG	PL	3011	3011		0	0
E700	HR-SURV	20	4	0	PL	normal	PL	1536	1536		0	0
410	LR-SURV	2	1	0	normal	WG	WL	2910	2910		0	0
414	LR-SURV	3	1	0				2951	2951		0	0
530	LR-SURV	2	1	0	normal	WG	normal	2216	2216		0	0
566	LR-SURV	0	2a	0	normal	WG	WL	1615	1615		0	0
744	LR-SURV	16	2b	0	normal	WG	normal	1850	1850		0	0
809	LR-SURV	0	1	0	normal	WG	normal	2904	2904		0	0
820	LR-SURV	11	2	0				4794	4794		0	0
822	LR-SURV	13	1	0	normal			1153	1153		0	0
823	LR-SURV	4	1	0	normal		normal	1090	1090		0	0
858	LR-SURV	1	1	0	normal		normal	1562	1562		0	0
912	LR-SURV	0	1	0				2904	2904		0	0
916	LR-SURV	10	1	0	normal	WG	WL	3830	3830		0	0
926	LR-SURV	1	1	0	normal	WG	WL	1861	1861		0	0
929	LR-SURV	3	1	0	normal	WG	WL	1076	1076		0	0
1028	LR-SURV	3	1	0	normal			2932	2932		0	0
1038	LR-SURV	11	2	0				1071	1071		0	0
1039	LR-SURV	8	3	0				1394	1394		0	0
1377	LR-SURV	3	2	0	normal		normal	2511	2511		0	0
1395	LR-SURV	1	1	0				2027	2027		0	0
1468	LR-SURV	7	2	0	WG	WG	WL	1808	1808		0	0
1476	LR-SURV	22	2	0				2075	2075		0	0
1478	LR-SURV	1	1	0				1066	1066		0	0
1479	LR-SURV	3	1	0	normal	WG	normal	1156	1156		0	0
1486	LR-SURV	12	1	0	normal	WG	normal	2328	2328		0	0
1487	LR-SURV	7	1	0				1463	1463		0	0
1488	LR-SURV	2	2	0				1827	1827		0	0
1492	LR-SURV	0	1	0				1316	1316		0	0
1528	LR-SURV	10	1	0				1622	1622		0	0
1531	LR-SURV	2	1	0	normal	WG	normal	1593	1593		0	0
1612	LR-SURV	6	1	0	normal	WG	normal	2271	2271		0	0
1650	LR-SURV	1	2	0	normal	WG	WL	1264	1264	187	0	1

LC480 clinical annotation

Characteristic	Classes	Count (percentage)
**Risk Classification**	HR-DOD	28/89 (31%)
	HR-SURV	30/89 (34%)
	LR-SURV	31/89 (35%)
**INSS Stage**	1	21/89 (24%)
	2	12/89 (13%)
	3	17/89 (19%)
	4	39/89 (44%)
MYCN	Not amplified	50/89 (56%)
	Amplified	39/89 (44%)
**Age**	Age at diagnosis > 12 months	53/89 (60%)
	Age at diagnosis < 12 months	36/89 (40%)

LC480 overview annotation

Name	Forward primer	Reverse primer	Genomic location
TGFBI_18014	ATTTTGGCGGGTTTCGTTA	ACTCGACTTACCCGTACTAAC	chr5:135364804-135364889
ACTB_1	TAGGGAGTATATAGGTTGGGGAAGTT	AACACACAATAACAAACACAAATTCAC	chr7:5571799-5571902
TNFRSF10D_23780	TCGGGGTATAAAAGCGGAG	ATCAAAAATCGTCCCCGTAA	chr8:23021486-23021578
KRT19_83159	GGTCGCGGGTATAAAAGGC	CATAACGAAACGAAACACGAAC	chr17:39684496-39684598
KRT19_61596	TTATAGTTATCGTTAGTCGTCGGT	ACCCCCGTAAATACTAAACGC	chr17:39684382-39684491
TNFRSF10A_65529	GGTATTTAGTAGGCGTTCGGTG	ACTCGCCCGATAATAACGAAA	chr8:23082862-23082978
RARRES1_57581	GCGAAATCGTAGGGGAAAC	ATAAAAACGCCTCCCCGAAA	chr3:158450497-158450595
CYGB_83388	TCGTGTGGGTAAGAGTTAATGTCG	CTCCGCCGTAACCTACGAA	chr17:74533911-74534004
FBN2_18150	TCGGAGTTTTATAGGGTAACGAA	CTCTTACTAACCGCACGCC	chr5:127873815-127873902
PDLIM4_4	GGCGTTTAGGTTAATTTTTCGT	CGATCCCATATCTAAAACCGA	chr5:131593549-131593644
CXCR4_70046	GCGTGGGTGTAGTGGGTAGTC	TAAAAACCTCCAAACGATAAACACG	chr2:136874383-136874463
RBP4_24106	GGTCGTTTCGTTGTTTTATAGC	GCGTTATACAAATACCCCCG	chr10:95361003-95361106
RBP1_83281	TGCGTTCGTTTTCGTTAAGC	CTAAATTCCCAACGCGCAC	chr3:139258517-139258597
NPTX2_57773	TTTTAGTTTGTGACGTTCGCGTT	TAAAACTCTCGAAAACCTCGACT	chr7:98246269-98246349
PPIC_69739	CGGTTGTTGTTATTTTTCGTGT	CTCTCGATCACCTTAACCGTC	chr5:122372192-122372305
NPY_18477	TTTTATTCGATGGCGTTGTC	AACAACTAACGATCTCCCTACGA	chr7:24324196-24324288
FAS_18143	TTTTTGATTATCGGGGTTTTTC	AACCAAATCACTCGTAAACCG	chr10:90750369-90750484
EDNRB_1	CGTTTTAGTTAAGCGTGCGTG	CGAAATATAAAAATTCCCCTACGA	chr13:78493099-78493204
DPP4_74913	TGGCGAAGAGGTTTTTATAGTTC	TACGCTCCTTCTCTAAACGCTC	chr2:162930431-162930534
FBN2_18151	TTGGAGATTTCGATAGAGCGT	AAACTACCGACTACACCTCCG	chr5:127873440-127873551
NTRK1_1259	TTTTACGGGATTAGATTTAAGTTTCGT	AACGAAAACGCGATAACCTT	chr1:156783919-156784031
NEBL_1678	TTTTGGGACGTAGGTTTTCG	AACCGTCTCTACTACGCG	chr10:21463545-21463628
PDE4DIP_1689	GCGTTTCGTCGGGTTTAT	AACTCCCGACTAACTCCG	chr1:145075684-145075799
PLXNC1_1397	CGAGTGCGGTAATTATCGTTATT	CCGTCTTAACCCGACTATAAAAC	chr12:94533764-94533877
PRPH_1409	GATTTGTTCGACGTTGTTAATCG	CTCGCACGTTAAACTCTAAATCTAA	chr12:49690936-49691035
CNR1_1699	AAATGAACGTTAAGGCGGTTATAA	AATCGCACGATTTCCACC	chr6:88877266-88877378
ADRB2_1431	TTTTATCGTTTTGGTTATCGTGTTTG	TAATAACCGTCTACAAACGCTCG	chr5:148206516-148206599
QPCT_1446	CGTAGTTGTATTTGAGCGGTT	ATAAAACGCCGATACCTAAACTAAAA	chr2:37572011-37572131
mir-663_1705	TTCGTCGTTTATTGTGGTCG	CAACCATCGCTCGCTATC	chr20:26189216-26189327
mir-671_1465	GAGTTAGAGCGACGGTATTTTG	CTTCGAAACCACGTCCATAAA	chr7:150935098-150935194
HIST1H3C_1491	AATAGTTCGTAAGTTTATCGGCG	ATACCCGAACGATAACGATAAAATTT	chr6:26045655-26045775
ACSS3_1504	TTTTAGTGGTTTCGGGTTCG	ACCGAAACTACGAAATAAATCTTATACTC	chr12:81472005-81472108
KCND2_1525	TTTTAGTGGTTTCGGGTTCG	ACCGAAACTACGAAATAAATCTTATACTC	chr12:81472005-81472108
GNAS_1532	TCGAATCGGAATTTGATTACGAG	CTCGATCTCGCTCTCGAT	chr20:57415399-57415509
COL6A3_1719	TGTATTTCGGGCGGTAGT	ACAAAACGAATCCCGAATAAATTTTAAA	chr2:238322242-238322326
TRIM36_1727	TAAACGTGGTGATTTCGAGTG	AATCGCACACTTCGAACC	chr5:114515219-114515317
SHOX2_1738	CGTTCGTTATCGGAGGTTG	GAAATCGCACGAACACAAAC	chr3:157821378-157821486
C3orf70_1749	GTTACGCGATGTATTCGGTT	CGAATAACACTAACCACCGAAAA	chr3:184871092-184871212
ANKRD43_1611	GGTTGTATTGTACGGTTACGAG	AAAACCGAACCGCAAATACTAATA	chr5:132150485-132150603
PCDHA6_16211	TTGGTGTCGCGTTTGTTTA	CGATCCGAAAATTCACAAACAAAATA	chr5:140207850-140207947
GRB10_1759	TTAGTTTACGATAGAGTACGGGG	ATAAACGAACTATTTAAAACGCACAAAA	chr7:50861529-50861646
APOE_1643	CGTTTGGACGAGGTGAAG	AACCTCGACCTACAAACGTA	chr19:45412288-45412375
PTGER4_1659	TGCGTCGTATTTCGTTGG	GCTAAAACGCGACAAAACTAAAA	chr5:40681747-40681851
RTN1_1765	TCGAGTTGGATAGCGAGTTTA	CGCTAAACTTCATCGAAAATAAATCC	chr14:60193921-60194023
mir-1225_1771	GTAGTTACGTTTCGTGCGTTA	CTAAACGTAAACTACCCCGAAC	chr16:2140686-2140801
mir-3177_1799	AGTGTAAACGTTAGGTAGTGTTTTC	AAAACAACGCCTAACACGTAT	chr16:1784907-1785023
TGFBI_1805	TCGTTTCGTTTTATGGCGT	TTAACGAAACCCGCCAAAA	chr5:135364732-135364824
TGFBI_1809	TTGTACGGGTCGGTTTAGT	ACGAACAAACGAACGACTC	chr5:135364587-135364707

LC480 assays

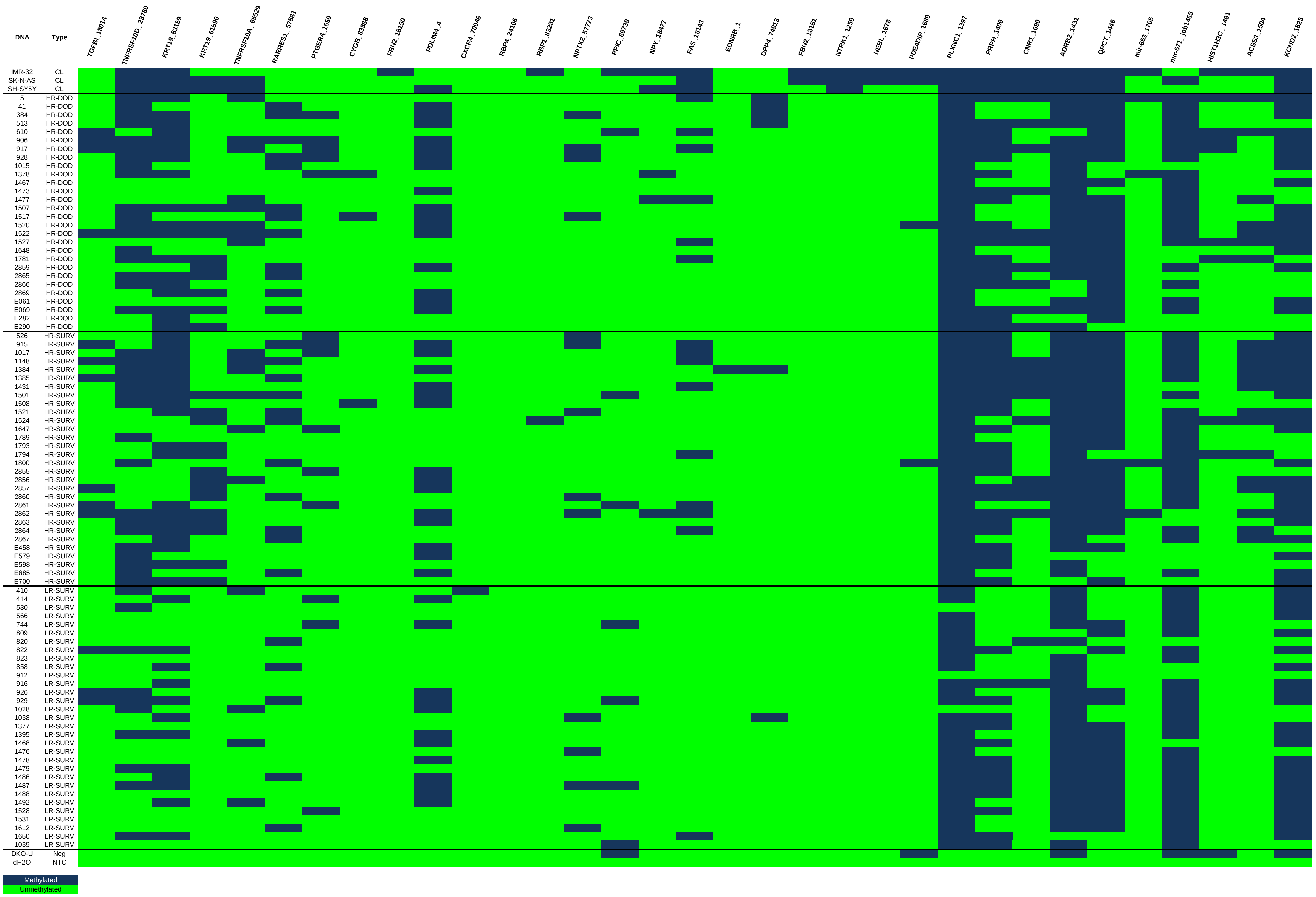

LC480 results

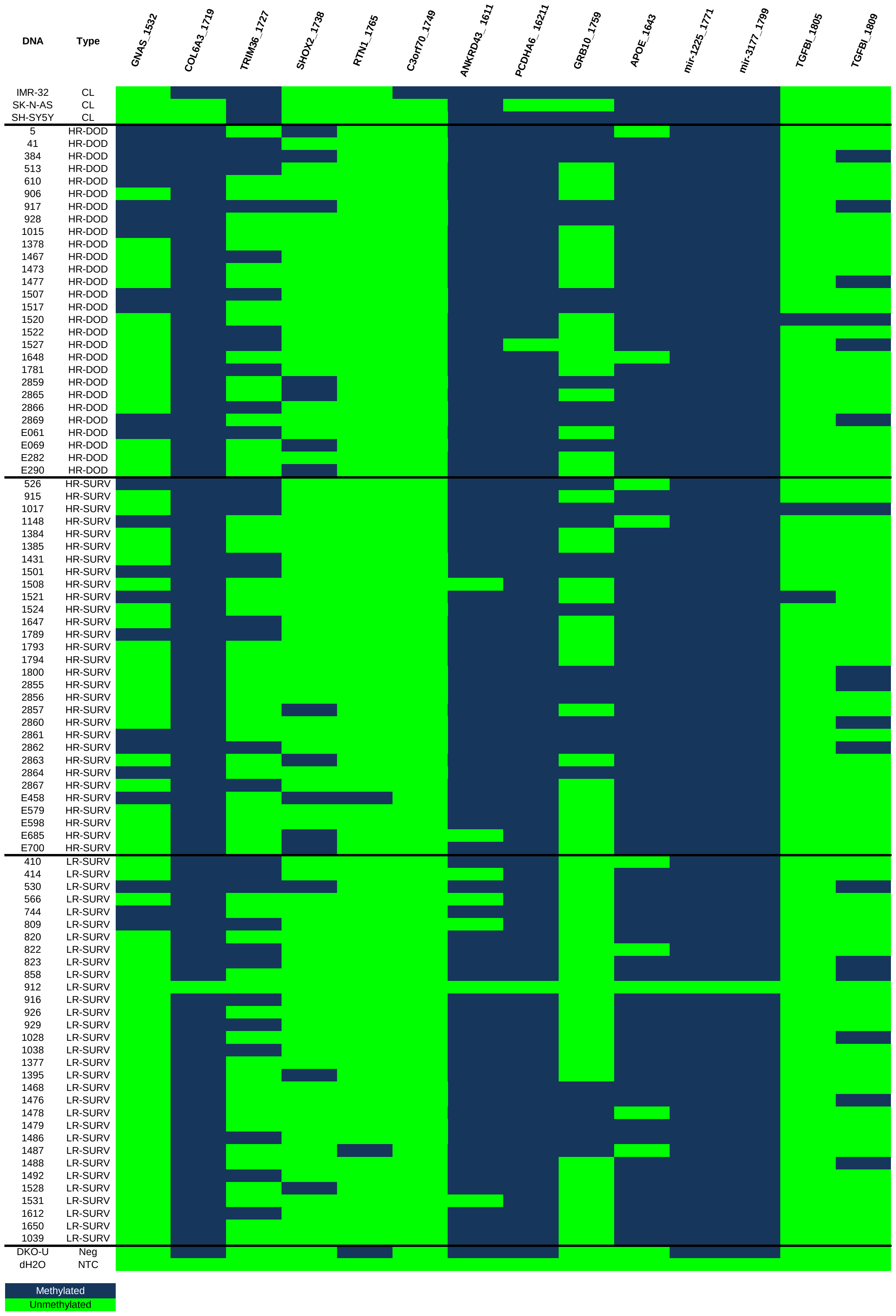

LC480 results

Gene	HR-DOD	HR-SURV	LR-SURV	Overall
COL6A3	28/28 (100%)	30/30 (100%)	30/31 (97%)	88/89 (99%)
miR-1225	28/28 (100%)	30/30 (100%)	30/31 (97%)	88/89 (99%)
miR-3177	28/28 (100%)	30/30 (100%)	30/31 (97%)	88/89 (99%)
PCDHA6	27/28 (96%)	30/30 (100%)	30/31 (97%)	87/89 (98%)
PLXNC1	28/28 (100%)	30/30 (100%)	28/31 (90%)	86/89 (97%)
ANKRD43	28/28 (100%)	28/30 (93%)	26/31 (84%)	82/89 (92%)
ADRB2	24/28 (86%)	28/30 (93%)	28/31 (90%)	80/89 (90%)
APOE	26/28 (93%)	28/30 (93%)	26/31 (84%)	80/89 (90%)
miR-671	21/28 (75%)	22/30 (73%)	27/31 (87%)	70/89 (79%)
QPCT	24/28 (86%)	25/30 (83%)	18/31 (58%)	67/89 (75%)
KCND2	18/28 (64%)	22/30 (73%)	23/31 (74%)	63/89 (71%)
PRPH	19/28 (68%)	24/30 (80%)	14/31 (45%)	57/89 (64%)
KRT19 (assay 83159)	18/28 (64%)	20/30 (67%)	12/31 (39%)	50/89 (56%)
TNFRSF10D	18/28 (64%)	17/30 (57%)	10/31 (32%)	45/89 (51%)
PDLIM4	16/28 (57%)	14/30 (47%)	12/31 (39%)	42/89 (47%)
TRIM36	11/28 (39%)	9/30 (30%)	12/31 (39%)	32/89 (36%)
GRB10	11/28 (39%)	13/30 (43%)	6/31 (19%)	30/89 (34%)
RARRES1	12/28 (43%)	11/30 (37%)	5/31 (16%)	28/89 (31%)
GNAS	12/28 (43%)	9/30 (30%)	3/31 (10%)	24/89 (27%)
KRT19 (assay 61596)	9/28 (32%)	14/30 (47%)	0/31 (0%)	23/89 (26%)
CNR1	10/28 (36%)	10/30 (33%)	2/31 (6%)	22/89 (25%)
ACSS3	7/28 (25%)	14/30 (47%)	0/31 (0%)	21/89 (24%)
TNFRSF10A	8/28 (29%)	6/30 (20%)	4/31 (13%)	18/89 (20%)
TGFBI (assay 1809)	6/28 (21%)	5/30 (17%)	6/31 (19%)	17/89 (19%)
FAS	6/28 (21%)	8/30 (27%)	1/31 (3%)	15/89 (17%)
SHOX2	7/28 (25%)	5/30 (17%)	3/31 (10%)	15/89 (17%)
PTGER4	5/28 (18%)	6/30 (20%)	3/31 (10%)	14/89 (16%)
TGFBI (assay 18014)	4/28 (14%)	6/30 (20%)	3/31 (10%)	13/89 (15%)
NPTX2	4/28 (14%)	5/30 (17%)	4/31 (13%)	13/89 (15%)
HIST1H3C	6/28 (21%)	2/30 (7%)	0/31 (0%)	8/89 (9%)
PPIC	1/28 (4%)	2/30 (7%)	4/31 (13%)	7/89 (8%)
DPP4	4/28 (14%)	1/30 (3%)	1/31 (3%)	6/89 (7%)
miR-663	2/28 (7%)	2/30 (7%)	0/31 (0%)	4/89 (4%)
CYGB	2/28 (7%)	1/30 (3%)	0/31 (0%)	3/89 (3%)
NPY	2/28 (7%)	1/30 (3%)	0/31 (0%)	3/89 (3%)
TGFBI (assay 1805)	1/28 (4%)	2/30 (7%)	0/31 (0%)	3/89 (3%)
PDE4DIP	1/28 (4%)	1/30 (3%)	0/31 (0%)	2/89 (2%)
RTN1	0/28 (0%)	1/30 (3%)	1/31 (3%)	2/89 (2%)
CXCR4	0/28 (0%)	0/30 (0%)	1/31 (3%)	1/89 (1%)
RBP1	0/28 (0%)	1/30 (3%)	0/31 (0%)	1/89 (1%)
EDNRB	0/28 (0%)	1/30 (3%)	0/31 (0%)	1/89 (1%)
FBN2 (assay 18150)	0/28 (0%)	0/30 (0%)	0/31 (0%)	0/89 (0%)
RBP4	0/28 (0%)	0/30 (0%)	0/31 (0%)	0/89 (0%)
FBN2 (assay 18151)	0/28 (0%)	0/30 (0%)	0/31 (0%)	0/89 (0%)
NTRK1	0/28 (0%)	0/30 (0%)	0/31 (0%)	0/89 (0%)
NEBL	0/28 (0%)	0/30 (0%)	0/31 (0%)	0/89 (0%)
C3orf70	0/28 (0%)	0/30 (0%)	0/31 (0%)	0/89 (0%)

Type	Stage	*MYCN*	Age at diagnosis	*TNFRSF10D*	*KRT19*	*FAS*	*PRPH*	*CNR1*	*QPCT*	*HIST1H3C*	*ACSS3*	*GNAS*
**HR-DOD**	3	amplified	> 12 months	2/3 (67%)	2/3 (67%)	0/3 (0%)	3/3 (100%)	2/3 (67%)	3/3 (100%)	1/3 (33%)	1/3 (33%)	0/3 (0%)
	4	single copy	> 12 months	9/11 (82%)	3/11 (27%)	0/11 (0%)	4/11 (36%)	2/11 (18%)	9/11 (82%)	0/11 (0%)	0/11 (0%)	6/11 (55%)
		amplified	> 12 months	6/10 (60%)	3/10 (30%)	6/10 (60%)	9/10 (90%)	4/10 (40%)	10/10 (100%)	5/10 (50%)	6/10 (60%)	5/10 (50%)
			< 12 months	1/4 (25%)	1/4 (25%)	0/4 (0%)	3/4 (75%)	2/4 (50%)	2/4 (50%)	0/4 (0%)	0/4 (0%)	1/4 (25%)
Total HR-DOD				*18/28 (64%)*	*9/28 (32%)*	*6/28 (21%)*	*19/28 (68%)*	*10/28 (36%)*	*24/28 (86%)*	*6/28 (21%)*	*7/28 (25%)*	*12/28 (43%)*
**HR-SURV**	2	amplified	> 12 months	1/1 (100%)	1/1 (100%)	1/1 (100%)	1/1 (100%)	1/1 (100%)	1/1 (100%)	0/1 (0%)	1/1 (100%)	1/1 (100%)
			< 12 months	1/2 (50%)	0/2 (0%)	0/2 (0%)	2/2 (100%)	0/2 (0%)	2/2 (100%)	0/2 (0%)	0/2 (0%)	0/2 (0%)
	3	amplified	> 12 months	5/13 (39%)	7/13 (54%)	4/13 (31%)	9/13 (69%)	7/13 (54%)	12/13 (92%)	2/13 (15%)	8/13 (62%)	3/13 (23%)
	4	single copy	> 12 months	6/7 (86%)	4/7 (57%)	1/7 (14%)	5/7 (71%)	1/7 (14%)	3/7 (43%)	0/7 (0%)	2/7 (29%)	2/7 (29%)
			< 12 months	1/1 (100%)	0/1 (0%)	0/1 (0%)	1/1 (100%)	0/1 (100%)	1/1 (100%)	0/1 (0%)	0/1 (0%)	1/1 (100%)
		amplified	> 12 months	2/5 (40%)	1/5 (20%)	2/5 (40%)	5/5 (100%)	1/5 (20%)	5/5 (100%)	0/5 (0%)	3/5 (60%)	2/5 (40%)
			< 12 months	1/1 (100%)	1/1 (100%)	0/1 (0%)	1/1 (100%)	0/1 (0%)	1/1 (100%)	0/1 (0%)	0/1 (0%)	0/1 (0%)
Total HR-SURV				*17/30 (57%)*	*14/30 (48%)*	*8/30 (27%)*	*24/30 (80%)*	*10/30 (33%)*	*25/30 (83%)*	*2/30 (7%)*	*14/30 (47%)*	*9/30 (30%)*
**LR-SURV**	1	single copy	> 12 months	1/1 (100%)	0/1 (0%)	0/1 (0%)	1/1 (100%)	0/1 (0%)	1/1 (100%)	0/1 (0%)	0/1 (0%)	0/1 (0%)
			< 12 months	8/20 (40%)	0/20 (0%)	0/20 (0%)	7/20 (35%)	1/20 (5%)	12/20 (60%)	0/20 (0%)	0/20 (0%)	2/20 (10%)
	2	single copy	> 12 months	0/2 (0%)	0/2 (0%)	0/2 (0%)	0/2 (0%)	0/2 (0%)	2/2 (100%)	0/2 (0%)	0/2 (0%)	1/2 (50%)
			< 12 months	1/7 (14%)	0/7 (0%)	1/7 (14%)	5/7 (71%)	1/7 (14%)	3/7 (43%)	0/7 (0%)	0/7 (0%)	0/7 (0%)
	3	single copy	< 12 months	0/1 (0%)	0/1 (0%)	0/1 (0%)	1/1 (100%)	0/1 (0%)	0/1 (0%)	0/1 (0%)	0/1 (0%)	0/1 (0%)
Total LR-SURV				*10/31 (32%)*	*0/31 (0%)*	*1/31 (3%)*	*14/31 (45%)*	*2/31 (6%)*	*18/31 (58%)*	*0/31 (0%)*	*0/31 (0%)*	*3/31 (10%)*
Overall total				***45/89 (51%)***	***23/89 (26%)***	***15/89 (17%)***	***57/89 (64%)***	***22/89 (25%)***	***67/89 (75%)***	***8/89 (9%)***	***21/89 (24%)***	***24/89 (27%)***

Group associations

Type	Stage	*MYCN*	Age at diagnosis	*TGFBI*	*TNFRSF10D*	*KRT19*	*TNFRSF10A*	*RARRES1*	*FAS*	*PRPH*	*CNR1*	*QPCT*	*HIST1H3C*	*ACSS3*	*GNAS*	*GRB10*
Stage	1	Single copy	> 12 months	1/1 (100%)	1/1 (100%)	0/1 (0%)	0/1 (0%)	0/1 (0%)	0/1 (0%)	1/1 (100%)	0/1 (0%)	1/1 (100%)	0/1 (100%)	0/1 (100%)	0/1 (0%)	0/1 (0%)
			< 12 months	2/20 (10%)	8/20 (40%)	0/20 (0%)	3/20 (15%)	4/20 (20%)	0/20 (0%)	7/20 (35%)	1/20 (5%)	12/20 (60%)	0/20 (0%)	0/20 (0%)	2/20 (10%)	4/20 (20%)
		Stage 1		*3/21 (14%)*	*9/21 (43%)*	*0/21 (0%)*	*3/21 (14%)*	*4/21 (19%)*	*0/21 (0%)*	*8/21 (38%)*	*1/21 (5%)*	*13/21 (62%)*	*0/21 (0%)*	*0/21 (0%)*	*2/21 (10%)*	*4/21 (19%)*
	2	Single copy	> 12 months	0/2 (0%)	0/2 (0%)	0/2 (0%)	0/2 (0%)	0/2 (0%)	0/2 (0%)	0/2 (0%)	0/2 (0%)	2/2 (100%)	0/2 (0%)	0/2 (0%)	1/2 (50%)	1/2 (50%)
			< 12 months	0/7 (100%)	1/7 (14%)	0/7 (0%)	1/7 (14%)	1/7 (14%)	1/7 (14%)	5/7 (71%)	1/7 (14%)	3/7 (43%)	0/7 (0%)	0/7 (0%)	0/7 (0%)	1/7 (14%)
		Amplified	> 12 months	1/1 (100%)	1/1 (100%)	1/1 (100%)	0/1 (0%)	0/1 (0%)	1/1 (100%)	1/1 (100%)	1/1 (100%)	1/1 (100%)	0/1 (0%)	1/1 (100%)	1/1 (100%)	1/1 (100%)
			< 12 months	0/2 (0%)	1/2 (0%)	0/2 (0%)	1/2 (50%)	0/2 (0%)	0/2 (0%)	2/2 (100%)	0/2 (0%)	2/2 (100%)	0/2 (0%)	0/2 (0%)	0/2 (0%)	0/2 (0%)
		Stage 2		*1/12 (8%)*	*3/12 (25%)*	*1/12 (8%)*	*2/12 (17%)*	*1/12 (8%)*	*2/12 (17%)*	*8/12 (67%)*	*2/12 (17%)*	*8/12 (67%)*	*0/12 (0%)*	*1/12 (8%)*	*2/12 (17%)*	*3/12 (25%)*
	3	Single copy	< 12 months	0/1 (0%)	0/1 (0%)	0/1 (0%)	0/1 (0%)	0/1 (0%)	0/1 (0%)	1/1 (100%)	0/1 (0%)	0/1 (0%)	0/1 (0%)	0/1 (0%)	0/1 (0%)	0/1 (0%)
		Amplified	> 12 months	6/16 (38%)	7/16 (44%)	9/16 (56%)	6/16 (38%)	7/16 (44%)	4/16 (25%)	12/16 (75%)	9/16 (56%)	15/16 (94%)	3/16 (19%)	9/16 (56%)	3/16 (19%)	8/16 (50%)
		Stage 3		*6/17 (35%)*	*7/17 (41%)*	*9/17 (53%)*	*6/17 (35%)*	*7/17 (41%)*	*4/17 (24%)*	*13/17 (77%)*	*9/17 (53%)*	*15/17 (88%)*	*3/17 (18%)*	*9/17 (58%)*	*3/17 (18%)*	*8/17 (47%)*
	4	Single copy	> 12 months	0/18 (0%)	15/18 (83%)	7/18 (39%)	1/18 (6%)	11/18 (61%)	1/18 (6%)	9/18 (50%)	3/18 (17%)	12/18 (67%)	0/18 (0%)	2/18 (11%)	8/18 (44%)	8/18 (44%)
			< 12 months	0/1 (0%)	1/1 (100%)	0/1 (0%)	0/1 (0%)	0/1 (0%)	0/1 (0%)	1/1 (100%)	0/1 (0%)	1/1 (100%)	0/1 (0%)	0/1 (0%)	1/1 (100%)	0/1 (0%)
		Amplified	> 12 months	3/15 (20%)	8/15 (53%)	4/15 (27%)	6/15 (40%)	4/15 (27%)	8/15 (53%)	14/15 (93%)	5/15 (33%)	15/15 (100%)	5/15 (33%)	9/15 (60%)	7/15 (47%)	6/15 (40%)
			< 12 months	0/5 (0%)	2/5 (40%)	2/5 (40%)	0/5 (0%)	1/5 (20%)	0/6 (0%)	4/5 (80%)	2/5 (40%)	3/5 (60%)	0/5 (0%)	0/5 (0%)	1/5 (20%)	1/5 (20%)
		Stage 4		*3/39 (7%)*	*26/39 (67%)*	*13/39 (33%)*	*7/39 (18%)*	*16/39 (41%)*	*9/39 (15%)*	*28/39 (72%)*	*10/39 (26%)*	*31/39 (80%)*	*5/39 (18%)*	*11/39 (28%)*	*17/39 (44%)*	*15/39 (39%)*
MYCN		*MYCN single copy*		*3/50 (6%)*	*26/50 (52%)*	*7/50 (14%)*	*5/50 (10%)*	*16/50 (32%)*	*2/50 (4%)*	*24/50 (48%)*	*5/50 (10%)*	*31/50 (62%)*	*0/50 (0%)*	*2/50 (4%)*	*12/50 (24%)*	*14/50 (28%)*
		MYCN amplified		*10/39 (26%)*	*19/39 (49%)*	*16/39 (41%)*	*13/39 (33%)*	*12/39 (31%)*	*13/39 (33%)*	*33/39 (85%)*	*17/39 (44%)*	*36/39 (92%)*	*8/39 (21%)*	*19/39 (49%)*	*12/39 (31%)*	*16/39 (41%)*
**Age**		*Age at diagnosis > 12 months*		*11/53 (21%)*	*32/53 (60%)*	*21/53 (40%)*	*13/53 (25%)*	*22/53 (42%)*	*14/53 (26%)*	*37/53 (69%)*	*18/53 (34%)*	*46/53 (87%)*	*8/53 (15%)*	*21/53 (40%)*	*20/53 (38%)*	*24/53 (45%)*
		Age at diagnosis < 12 months		*2/36 (6%)*	*13/36 (36%)*	*2/36 (6%)*	*5/36 (14%)*	*6/36 (17%)*	*1/36 (3%)*	*20/396(56%)*	*4/36 (11%)*	*21/36 (58%)*	*0/36 (0%)*	*0/36 (0%)*	*4/36 (11%)*	*6/36 (17%)*
		Age at diagnosis > 18 months		*9/45 (20%)*	*27/45 (60%)*	*20/45 (44%)*	*11/45 (24%)*	*19/45 (42%)*	*13/45 (29%)*	*33/45 (73%)*	*17/45 (38%)*	*40/45 (89%)*	*8/45 (18%)*	*19/45 (49%)*	*18/45 (40%)*	*23/45 (51%)*
		Age at diagnosis < 18 months		*4/44 (9%)*	*18/44 (41%)*	*3/44 (7%)*	*7/44 (16%)*	*9/44 (20%)*	*2/44 (5%)*	*24/44 (55%)*	*5/44 (11%)*	*27/44 (61%)*	*0/44 (0%)*	*2/44 (5%)*	*6/44 (14%)*	*7/44 (16%)*
		Overall total		***13/89 (15%)***	***45/89 (51%)***	***23/89 (26%)***	***18/89 (20%)***	***28/89 (32%)***	***15/89 (17%)***	***57/89 (64%)***	***22/89 (25%)***	***67/89 (75%)***	***8/89 (9%)***	***21/89 (24%)***	***24/89 (27%)***	***30/89 (34%)***

Risk factor associations

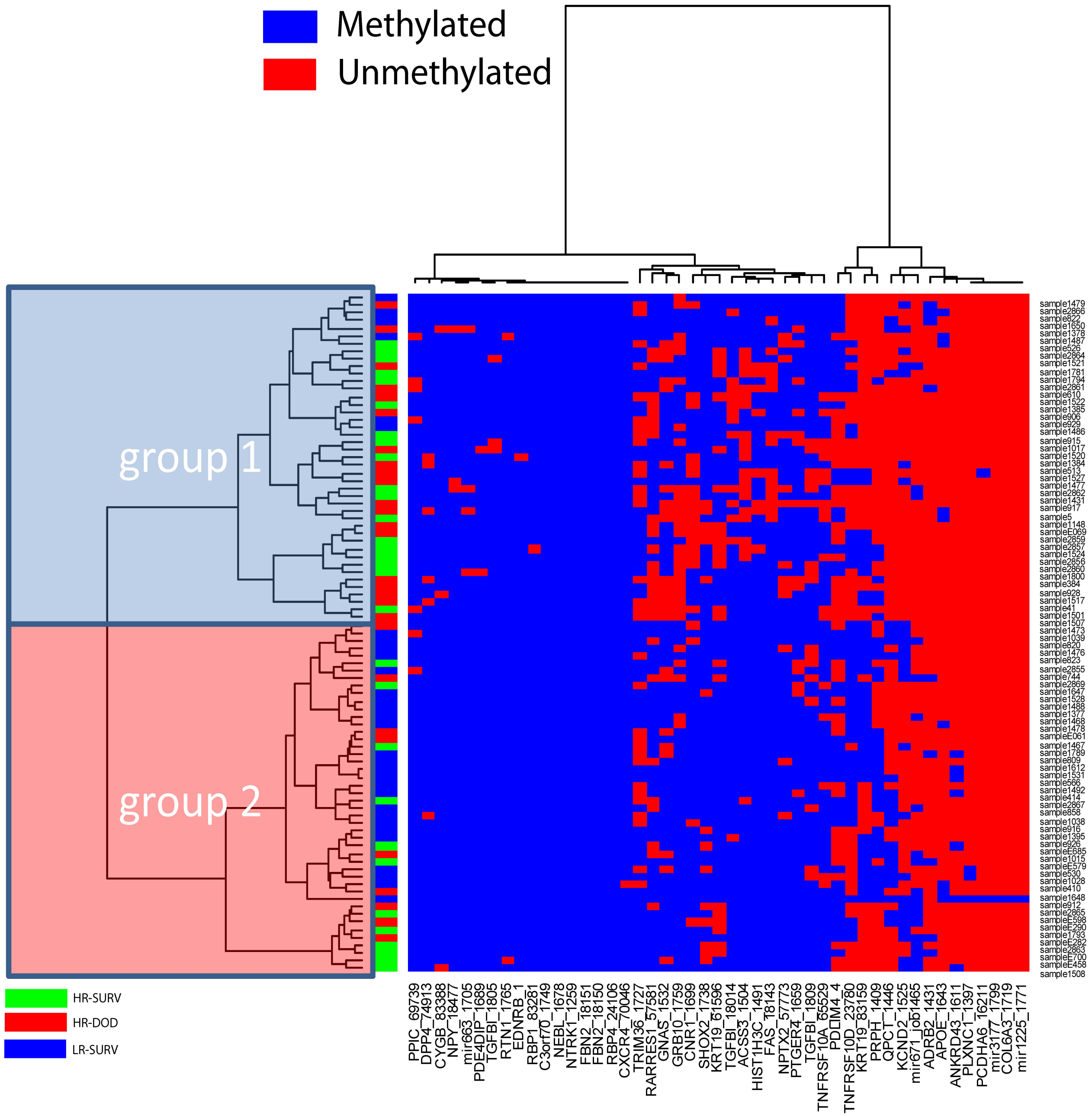
Methylated
Unmethylated
group 1
group 2
HR-SURV
HR-DOD
LR-SURV

Heatmap

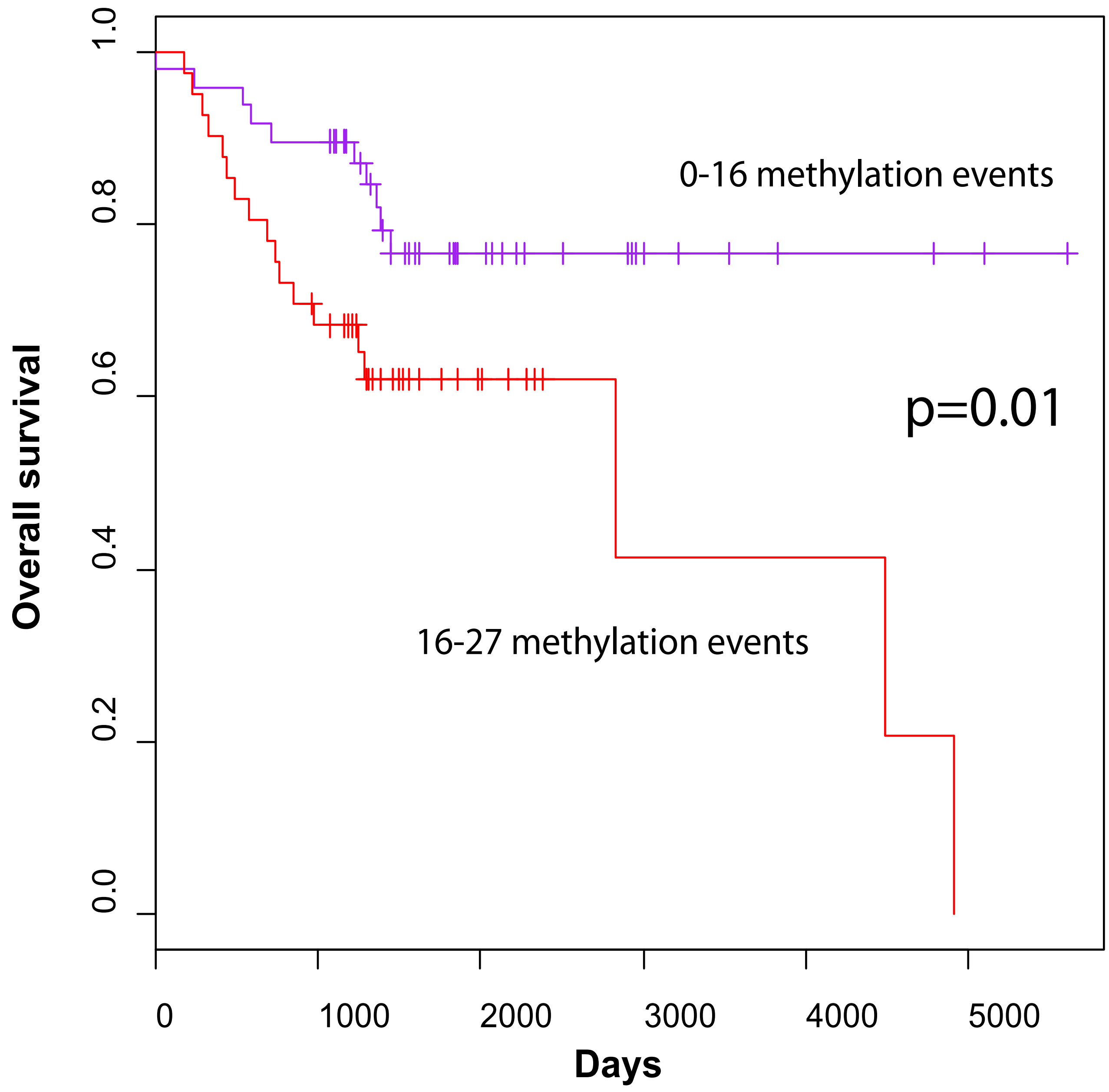
Overall survival
1.0
0.8
0.6
0.4
0.2
0.0
0
1000
2000
3000
4000
5000
Days
0-16 methylation events
16-27 methylation events
p=0.01

KM number
