# Supplementary material for: Genome-wide promoter methylation analysis in neuroblastoma identifies prognostic methylation biomarkers
Source: Genome Biol. 2012 Oct 3;13(10):R95. doi: 10.1186/gb-2012-13-10-r95 (PMC3491423; doi:10.1186/gb-2012-13-10-r95)
Supplement: Additional file 4 — Quantitative PCR and MSP assays for HIST1H3C and ACSS3 and matched results (expression levels - methylation call) for a panel of 31 NB cell lines. [file gb-2012-13-10-r95-S4.pdf]

**Part SIOPEN clinical annotation**: Patient characteristics of the samples of the neuroblastoma SIOPEN/GPOH cDNA library. PatientID is a unique patient number, the group indicates the risk. Clinical characteristics given are the age at diagnosis in months, International Neuroblastoma Staging System (INSS) stage, *MYCN* status (0 is non-amplified/single copy and 1 is amplified), overall survival (OS) and event-free survival (EFS) time in days and months after diagnosis, respectively. OS indicates whether the patient was alive (0) at the last known follow-up or died of disease (1). Similar for EFS, indicating events, such as relapse or progression. Empty cells represent missing values.

**Part SIOPEN overview annotation**: Summary of the clinical characteristics of the 366 primary neuroblastoma samples of the neuroblastoma SIOPEN/GPOH cDNA library. HR-DOD: high-risk deceased patients, HR-SURV: high-risk patients alive for at least 1000 days follow-up, LR-SURV: low-risk patients alive for at least 1000 days follow-up, and INSS: International Neuroblastoma Staging System.

**Part SIOPEN assays**: qPCR mRNA assays used in the mRNA expression profiling of the DNA-methylation biomarkers *CNR1*, *GRB10*, *KRT19*, *PRPH* and *QPCT*. For each tested gene, the assay name and corresponding forward and reverse primer (5' to 3') are indicated, as well as the genomic location of the amplicon on the hg19 reference genome.

**Part SIOPEN results**: Results of the mRNA expression measurement of the DNA-methylation biomarkers *CNR1, GRB10, KRT19, PRPH* and *QPCT*. The Cq values were converted to relative quantities and log2 values. Relative gene expression levels were then normalized using the geometric mean of five reference sequences (*HPRT1*, *SDHA*, *UBC*,

*HMBS* and *AluSq*). These logged and normalized qPCR data are given. HR-DOD: high-risk deceased patients, HR-SURV: high-risk patients alive for at least 1000 days follow-up and LR-SURV: low-risk patients alive for at least 1000 days follow-up.

**Part Expression**: Relative mRNA expression distribution of *CNR1*, *GRB10*, *KRT19*, *PRPH* and *QPCT* in each of the five different neuroblastoma tumor stages (stage 1, 2, 3, 4 and 4S). In the box plots, the right and left hinge of the boxes represents the 75th percentile and 25th percentile, respectively. Whiskers, lines that extend from the box to the highest and lowest values, indicate the data range. Significant p-values according to the Kruskal-Wallis test (corrected for multiple testing using Benjamini-Hochberg) are indicated.

**Part Survival**: Kaplan-Meier plots: overall survival in the neuroblastoma SIOPEN/GPOH samples according to the relative mRNA expression levels of *CNR1*, *GRB10*, *KRT19*, *PRPH* and *QPCT*. Survival curves indicated with ‘High’ and ‘Low’ are the samples assigned to the high and low mRNA expression group, respectively, using the median relative mRNA expression value as a cut-off to create the groups. Significant p-values according to the log-rank test (Mantel-Cox; corrected for multiple testing using Benjamini-Hochberg) are indicated. Time is indicated in days, starting from diagnosis and censored to 4000 days (censored samples are indicated with vertical lines crossing the overall survival curves).

| PatientID | Group | Age at diagnosis (months) | INSS Stage | *MYCN* | OS time (days) | EFS time (months) | OS | EFS |
|---|---|---|---|---|---|---|---|---|
| 194 | HR-DOD | 31.496 | 4 | 0 | 357 | 8.942 | 1 | 1 |
| 1500 | HR-DOD | 43.660 | 4 | 0 | 1260 | 23.605 | 1 | 1 |
| 1501 | HR-DOD | 46.685 | 4 | 0 | 689 | 11.803 | 1 | 1 |
| 1503 | HR-DOD | 113.129 | 4 | 0 | 1517 | 28.110 | 1 | 1 |
| 1716 | HR-DOD | 69.534 | 4 | 0 | 877 | 23.934 | 1 | 1 |
| 1852 | HR-DOD | 53.162 | 4 | 0 | 1221 | 11.211 | 1 | 1 |
| 2164 | HR-DOD | 124.537 | 4 | 0 | 1201 | 34.882 | 1 | 1 |
| 2710 | HR-DOD | 42.838 | 4 | 0 | 1235 | 27.156 | 1 | 1 |
| 2711 | HR-DOD | 85.578 | 4 | 0 | 890 | 19.101 | 1 | 1 |
| 2712 | HR-DOD | 110.334 | 4 | 0 | 100 | 3.288 | 1 | 0 |
| 2714 | HR-DOD | 56.647 | 4 | 0 | 1263 | 22.619 | 1 | 1 |
| 2719 | HR-DOD | 23.704 | 4 | 0 | 319 | 3.222 | 1 | 1 |
| 2752 | HR-DOD | 54.707 | 4 | 0 | 1785 | 23.704 | 1 | 1 |
| 2771 | HR-DOD | 70.356 | 4 | 0 | 1333 | 33.271 | 1 | 1 |
| 2772 | HR-DOD | 142.488 | 4 | 0 | 861 | 16.077 | 1 | 1 |
| 2775 | HR-DOD | 75.584 | 4 | 0 | 1446 | 36.033 | 1 | 1 |
| 2780 | HR-DOD | 45.238 | 4 | 0 | 1149 | 20.121 | 1 | 1 |
| 2788 | HR-DOD | 37.348 | 4 | 0 | 670 | 18.608 | 1 | 1 |
| 1536 | HR-DOD | 19.759 | 2 | 1 | 316 | 9.370 | 1 | 1 |
| 1822 | HR-DOD | 22.619 | 3 | 1 | 728 | 19.529 | 1 | 1 |
| 1908 | HR-DOD | 11.868 | 3 | 1 | 292 | 6.641 | 1 | 1 |
| 2090 | HR-DOD | 36.855 | 3 | 1 | 274 | 8.416 | 1 | 1 |
| 111 | HR-DOD | 46.455 | 4 | 1 | 268 | 8.351 | 1 | 1 |
| 127 | HR-DOD | 11.737 | 4 | 1 | 412 | 9.403 | 1 | 1 |
| 772 | HR-DOD | 91.595 | 4 | 1 | 485 | 14.433 | 1 | 1 |
| 939 | HR-DOD | 19.299 | 4 | 1 | 516 | 16.208 | 1 | 1 |
| 1231 | HR-DOD | 35.803 | 4 | 1 | 544 | 15.616 | 1 | 1 |
| 1496 | HR-DOD | 16.142 | 4 | 1 | 386 | 11.605 | 1 | 1 |
| 1498 | HR-DOD | 39.288 | 4 | 1 | 309 | 7.332 | 1 | 1 |
| 1499 | HR-DOD | 73.381 | 4 | 1 | 172 | 5.326 | 1 | 1 |
| 1515 | HR-DOD | 33.797 | 4 | 1 | 549 | 10.126 | 1 | 1 |
| 1758 | HR-DOD | 21.764 | 4 | 1 | 264 | 5.129 | 1 | 1 |
| 1776 | HR-DOD | 0.000 | 4 | 1 | 1 | 0.033 | 1 | 1 |
| 1784 | HR-DOD | 7.529 | 4 | 1 | 239 | 3.814 | 1 | 1 |
| 1788 | HR-DOD | 78.707 | 4 | 1 | 1246 | 29.523 | 1 | 1 |
| 1820 | HR-DOD | 39.748 | 4 | 1 | 1279 | 18.148 | 1 | 1 |
| 1827 | HR-DOD | 107.934 | 4 | 1 | 319 | | 1 | 1 |
| 1924 | HR-DOD | 27.551 | 4 | 1 | 340 | 10.784 | 1 | 1 |
| 1936 | HR-DOD | 54.871 | 4 | 1 | 679 | 21.534 | 1 | 1 |
| 2015 | HR-DOD | 41.392 | 4 | 1 | 663 | 19.660 | 1 | 1 |
| 2659 | HR-DOD | 23.605 | 4 | 1 | 201 | 6.378 | 1 | 1 |
| 2709 | HR-DOD | 32.121 | 4 | 1 | 290 | 8.416 | 1 | 1 |
| 2768 | HR-DOD | 13.315 | 4 | 1 | 294 | 9.600 | 1 | 1 |
| 2784 | HR-DOD | 13.381 | 4 | 1 | 226 | 4.340 | 1 | 1 |
| 2769 | HR-DOD | 41.293 | 4 | | 499 | 12.395 | 1 | 1 |
| 2777 | HR-DOD | 58.849 | 4 | | 1350 | 26.959 | 1 | 1 |
| 1497 | HR-SURV | 14.992 | 4 | 0 | 1628 | 53.523 | 0 | 0 |
| 1517 | HR-SURV | 49.644 | 4 | 0 | 1067 | 24.658 | 0 | 1 |
| 1677 | HR-SURV | 70.093 | 4 | 0 | 1114 | 36.625 | 0 | 0 |
| 1829 | HR-SURV | 34.192 | 4 | 0 | 2132 | 24.986 | 0 | 1 |
| 2016 | HR-SURV | 54.904 | 4 | 0 | 1569 | 32.153 | 0 | 1 |
| 2017 | HR-SURV | 54.904 | 4 | 0 | 1569 | 32.153 | 0 | 1 |
| 2092 | HR-SURV | 106.323 | 4 | 0 | 1267 | 35.244 | 0 | 1 |
| 2093 | HR-SURV | 28.438 | 4 | 0 | 1091 | 35.868 | 0 | 0 |
| 2171 | HR-SURV | 40.701 | 4 | 0 | 1218 | 40.044 | 0 | 0 |
| 2172 | HR-SURV | 23.079 | 4 | 0 | 1317 | 43.299 | 0 | 0 |
| 2718 | HR-SURV | 15.222 | 4 | 0 | 1913 | 62.893 | 0 | 0 |
| 2758 | HR-SURV | 38.203 | 4 | 0 | 1691 | 55.595 | 0 | 0 |
| 2762 | HR-SURV | 15.353 | 4 | 0 | 1641 | 53.951 | 0 | 0 |
| 2776 | HR-SURV | 80.614 | 4 | 0 | 1071 | 35.211 | 0 | 0 |
| 2781 | HR-SURV | 35.079 | 4 | 0 | 1161 | 29.195 | 0 | 1 |
| 1540 | HR-SURV | 18.707 | 2 | 1 | 2175 | 71.507 | 0 | 0 |
| 1766 | HR-SURV | 9.008 | 2 | 1 | 3153 | 103.660 | 0 | 0 |
| 1815 | HR-SURV | 5.655 | 2 | 1 | 1449 | 47.638 | 0 | 0 |

| PatientID | Group | Age at diagnosis (months) | INSS Stage | *MYCN* | OS time (days) | EFS time (months) | OS | EFS |
|---|---|---|---|---|---|---|---|---|
| 2162 | HR-SURV | 2.104 | 2 | 1 | 1837 | 60.395 | 0 | 0 |
| 1686 | HR-SURV | 16.373 | 3 | 1 | 1046 | 34.389 | 0 | 0 |
| 1824 | HR-SURV | 23.310 | 3 | 1 | 2387 | 78.477 | 0 | 0 |
| 2770 | HR-SURV | 26.170 | 3 | 1 | 2137 | 70.258 | 0 | 0 |
| 1759 | HR-SURV | 25.874 | 4 | 1 | 1953 | 64.208 | 0 | 0 |
| 1821 | HR-SURV | 38.038 | 4 | 1 | 2163 | 71.112 | 0 | 0 |
| 1823 | HR-SURV | 15.945 | 4 | 1 | 1985 | 65.260 | 0 | 0 |
| 1853 | HR-SURV | 8.745 | 4 | 1 | 2174 | 71.474 | 0 | 0 |
| 2091 | HR-SURV | 19.660 | 4 | 1 | 1320 | 43.397 | 0 | 0 |
| 2530 | HR-SURV | 26.433 | 4 | 1 | 2009 | 66.049 | 0 | 0 |
| 1542 | HR-SURV | 42.970 | 3 | 1 | 1807 | 59.408 | 0 | 0 |
| 116 | LR-SURV | 5.688 | 1 | 0 | 2575 | 84.658 | 0 | 0 |
| 126 | LR-SURV | 3.551 | 1 | 0 | 2693 | 88.537 | 0 | 0 |
| 130 | LR-SURV | 14.532 | 1 | 0 | 3404 | 111.912 | 0 | 0 |
| 266 | LR-SURV | 1.545 | 1 | 0 | 2216 | 72.855 | 0 | 0 |
| 315 | LR-SURV | 1.677 | 1 | 0 | 2910 | 95.671 | 0 | 0 |
| 320 | LR-SURV | 2.959 | 1 | 0 | 2951 | 97.019 | 0 | 0 |
| 321 | LR-SURV | 5.227 | 1 | 0 | 2938 | 96.592 | 0 | 0 |
| 338 | LR-SURV | 0.164 | 1 | 0 | 2904 | 95.474 | 0 | 0 |
| 346 | LR-SURV | 7.989 | 1 | 0 | 2302 | 75.682 | 0 | 0 |
| 769 | LR-SURV | 1.249 | 1 | 0 | 1562 | 51.353 | 0 | 0 |
| 824 | LR-SURV | 0.921 | 1 | 0 | 2735 | 89.918 | 0 | 0 |
| 827 | LR-SURV | 2.959 | 1 | 0 | 2732 | 89.819 | 0 | 0 |
| 1538 | LR-SURV | 15.485 | 1 | 0 | 2172 | 71.408 | 0 | 0 |
| 1539 | LR-SURV | 0.000 | 1 | 0 | 3922 | 128.942 | 0 | 0 |
| 1541 | LR-SURV | 14.827 | 1 | 0 | 1797 | 59.079 | 0 | 0 |
| 1693 | LR-SURV | 14.000 | 1 | 0 | 2372 | 79.067 | 0 | 0 |
| 1694 | LR-SURV | 15.600 | 1 | 0 | 2908 | 96.933 | 0 | 0 |
| 1698 | LR-SURV | 16.467 | 1 | 0 | 3303 | 110.100 | 0 | 0 |
| 1699 | LR-SURV | 3.367 | 1 | 0 | 2367 | 5.600 | 0 | 1 |
| 1700 | LR-SURV | 8.733 | 1 | 0 | 2416 | 1.100 | 0 | 1 |
| 1701 | LR-SURV | 25.267 | 1 | 0 | 1063 | 12.100 | 0 | 1 |
| 1720 | LR-SURV | 1.052 | 1 | 0 | 2027 | 66.641 | 0 | 0 |
| 1752 | LR-SURV | 33.008 | 1 | 0 | 1482 | 48.723 | 0 | 0 |
| 1767 | LR-SURV | 13.841 | 1 | 0 | 3930 | 129.205 | 0 | 0 |
| 1789 | LR-SURV | 1.479 | 1 | 0 | 1066 | 35.047 | 0 | 0 |
| 1790 | LR-SURV | 3.222 | 1 | 0 | 1156 | 38.005 | 0 | 0 |
| 1798 | LR-SURV | 11.507 | 1 | 0 | 2328 | 76.537 | 0 | 0 |
| 1799 | LR-SURV | 7.101 | 1 | 0 | 1463 | 48.099 | 0 | 0 |
| 1804 | LR-SURV | 0.296 | 1 | 0 | 1316 | 43.266 | 0 | 0 |
| 1828 | LR-SURV | 9.567 | 1 | 0 | 1622 | 53.326 | 0 | 0 |
| 1831 | LR-SURV | 2.137 | 1 | 0 | 1593 | 52.373 | 0 | 0 |
| 1841 | LR-SURV | 6.312 | 1 | 0 | 2271 | 74.663 | 0 | 0 |
| 1857 | LR-SURV | 7.397 | 1 | 0 | 1744 | 57.337 | 0 | 0 |
| 1879 | LR-SURV | 75.682 | 1 | 0 | 3050 | 100.274 | 0 | 0 |
| 1882 | LR-SURV | 14.827 | 1 | 0 | 4532 | 66.542 | 0 | 1 |
| 1883 | LR-SURV | 2.071 | 1 | 0 | 3436 | 112.964 | 0 | 0 |
| 1886 | LR-SURV | 4.142 | 1 | 0 | 2774 | 91.200 | 0 | 0 |
| 1891 | LR-SURV | 24.263 | 1 | 0 | 2593 | 85.249 | 0 | 0 |
| 1892 | LR-SURV | 60.690 | 1 | 0 | 3703 | 121.742 | 0 | 0 |
| 1893 | LR-SURV | 18.542 | 1 | 0 | 3173 | 104.318 | 0 | 0 |
| 1896 | LR-SURV | 32.910 | 1 | 0 | 3398 | 111.715 | 0 | 0 |
| 1898 | LR-SURV | 53.622 | 1 | 0 | 2478 | 81.468 | 0 | 0 |
| 1899 | LR-SURV | 2.005 | 1 | 0 | 2126 | 69.896 | 0 | 0 |
| 1901 | LR-SURV | 4.603 | 1 | 0 | 2931 | 96.362 | 0 | 0 |
| 1905 | LR-SURV | 1.184 | 1 | 0 | 3196 | 105.074 | 0 | 0 |
| 1906 | LR-SURV | 15.748 | 1 | 0 | 2022 | 66.477 | 0 | 0 |
| 1907 | LR-SURV | 25.545 | 1 | 0 | 1992 | 65.490 | 0 | 0 |
| 1909 | LR-SURV | 0.000 | 1 | 0 | 2320 | 76.274 | 0 | 0 |
| 1912 | LR-SURV | 2.170 | 1 | 0 | 2543 | 83.605 | 0 | 0 |
| 1914 | LR-SURV | 67.923 | 1 | 0 | 2481 | 81.567 | 0 | 0 |
| 1915 | LR-SURV | 25.611 | 1 | 0 | 2966 | 18.411 | 0 | 1 |
| 1916 | LR-SURV | 1.118 | 1 | 0 | 1675 | 55.068 | 0 | 0 |
| 1917 | LR-SURV | 4.471 | 1 | 0 | 2762 | 90.805 | 0 | 0 |

| PatientID | Group | Age at diagnosis (months) | INSS Stage | *MYCN* | OS time (days) | EFS time (months) | OS | EFS |
|---|---|---|---|---|---|---|---|---|
| 1918 | LR-SURV | 78.115 | 1 | 0 | 2432 | 79.956 | 0 | 0 |
| 1919 | LR-SURV | 60.164 | 1 | 0 | 1618 | 53.195 | 0 | 0 |
| 1920 | LR-SURV | 45.863 | 1 | 0 | 2721 | 89.458 | 0 | 0 |
| 1929 | LR-SURV | 4.603 | 1 | 0 | 1100 | 36.164 | 0 | 0 |
| 1939 | LR-SURV | 16.567 | 1 | 0 | 2194 | 73.133 | 0 | 0 |
| 1940 | LR-SURV | 16.533 | 1 | 0 | 2101 | 70.033 | 0 | 0 |
| 1941 | LR-SURV | 18.200 | 1 | 0 | 2639 | 87.967 | 0 | 0 |
| 1942 | LR-SURV | 15.600 | 1 | 0 | 2908 | 96.933 | 0 | 0 |
| 1943 | LR-SURV | 15.433 | 1 | 0 | 1372 | 45.733 | 0 | 0 |
| 1950 | LR-SURV | 13.367 | 1 | 0 | 3802 | 126.733 | 0 | 0 |
| 1959 | LR-SURV | 33.867 | 1 | 0 | 5019 | 167.300 | 0 | 0 |
| 1961 | LR-SURV | 4.400 | 1 | 0 | 4401 | 3.500 | 0 | 1 |
| 1964 | LR-SURV | 15.500 | 1 | 0 | 2317 | 77.233 | 0 | 0 |
| 1965 | LR-SURV | 14.667 | 1 | 0 | 3031 | 101.033 | 0 | 0 |
| 1968 | LR-SURV | 1.167 | 1 | 0 | 1685 | 56.167 | 0 | 0 |
| 2025 | LR-SURV | 13.414 | 1 | 0 | 2413 | 79.332 | 0 | 0 |
| 2071 | LR-SURV | 10.784 | 1 | 0 | 2566 | 84.362 | 0 | 0 |
| 2088 | LR-SURV | 1.085 | 1 | 0 | 1341 | 44.088 | 0 | 0 |
| 2099 | LR-SURV | 1.644 | 1 | 0 | 3926 | 129.074 | 0 | 0 |
| 2100 | LR-SURV | 162.740 | 1 | 0 | 2501 | 82.225 | 0 | 0 |
| 2101 | LR-SURV | 81.205 | 1 | 0 | 3590 | 118.027 | 0 | 0 |
| 2105 | LR-SURV | 39.649 | 1 | 0 | 2872 | 94.422 | 0 | 0 |
| 2106 | LR-SURV | 12.822 | 1 | 0 | 2806 | 92.252 | 0 | 0 |
| 2107 | LR-SURV | 1.841 | 1 | 0 | 3070 | 100.932 | 0 | 0 |
| 2108 | LR-SURV | 4.044 | 1 | 0 | 1803 | 59.277 | 0 | 0 |
| 2110 | LR-SURV | 6.970 | 1 | 0 | 2630 | 86.466 | 0 | 0 |
| 2119 | LR-SURV | 0.099 | 1 | 0 | 2270 | 74.630 | 0 | 0 |
| 2122 | LR-SURV | 2.038 | 1 | 0 | 1826 | 60.033 | 0 | 0 |
| 2161 | LR-SURV | 3.222 | 1 | 0 | 1826 | 60.033 | 0 | 0 |
| 2163 | LR-SURV | 3.781 | 1 | 0 | 1674 | 13.841 | 0 | 1 |
| 2165 | LR-SURV | 1.151 | 1 | 0 | 1567 | 51.518 | 0 | 0 |
| 2167 | LR-SURV | 2.137 | 1 | 0 | 1481 | 48.690 | 0 | 0 |
| 2169 | LR-SURV | 0.559 | 1 | 0 | 1461 | 48.033 | 0 | 0 |
| 2170 | LR-SURV | 1.742 | 1 | 0 | 1461 | 48.033 | 0 | 0 |
| 2253 | LR-SURV | 53.589 | 1 | 0 | 3860 | 126.904 | 0 | 0 |
| 2257 | LR-SURV | 0.427 | 1 | 0 | 1930 | 63.452 | 0 | 0 |
| 2753 | LR-SURV | 1.052 | 1 | 0 | 4483 | 9.732 | 0 | 1 |
| 2756 | LR-SURV | 0.296 | 1 | 0 | 3493 | 3.419 | 0 | 1 |
| 2794 | LR-SURV | 1.479 | 1 | 0 | 2780 | 3.123 | 0 | 1 |
| 2795 | LR-SURV | 0.921 | 1 | 0 | 2231 | 2.696 | 0 | 1 |
| 192 | LR-SURV | 1.447 | 2 | 0 | 1053 | 34.619 | 0 | 0 |
| 332 | LR-SURV | 3.156 | 2 | 0 | 2942 | 96.723 | 0 | 0 |
| 1535 | LR-SURV | 3.814 | 2 | 0 | 2613 | 85.907 | 0 | 0 |
| 1672 | LR-SURV | 2.532 | 2 | 0 | 2511 | 82.553 | 0 | 0 |
| 1695 | LR-SURV | 13.800 | 2 | 0 | 2094 | 69.800 | 0 | 0 |
| 1696 | LR-SURV | 32.500 | 2 | 0 | 2780 | 92.667 | 0 | 0 |
| 1697 | LR-SURV | 13.900 | 2 | 0 | 3344 | 111.467 | 0 | 0 |
| 1753 | LR-SURV | 19.200 | 2 | 0 | 3479 | 114.378 | 0 | 0 |
| 1754 | LR-SURV | 7.364 | 2 | 0 | 3117 | 102.477 | 0 | 0 |
| 1755 | LR-SURV | 2.827 | 2 | 0 | 1915 | 62.959 | 0 | 0 |
| 1756 | LR-SURV | 10.619 | 2 | 0 | 1532 | 50.367 | 0 | 0 |
| 1757 | LR-SURV | 33.929 | 2 | 0 | 2557 | 84.066 | 0 | 0 |
| 1762 | LR-SURV | 54.773 | 2 | 0 | 2107 | 69.271 | 0 | 0 |
| 1763 | LR-SURV | 37.479 | 2 | 0 | 1325 | 43.562 | 0 | 0 |
| 1764 | LR-SURV | 0.789 | 2 | 0 | 1470 | 48.329 | 0 | 0 |
| 1769 | LR-SURV | 6.148 | 2 | 0 | 2163 | 71.112 | 0 | 0 |
| 1770 | LR-SURV | 5.392 | 2 | 0 | 1737 | 57.107 | 0 | 0 |
| 1779 | LR-SURV | 6.773 | 2 | 0 | 1808 | 59.441 | 0 | 0 |
| 1787 | LR-SURV | 22.225 | 2 | 0 | 2075 | 68.219 | 0 | 0 |
| 1800 | LR-SURV | 2.433 | 2 | 0 | 1827 | 60.066 | 0 | 0 |
| 1855 | LR-SURV | 0.855 | 2 | 0 | 1264 | 6.148 | 0 | 1 |
| 1885 | LR-SURV | 12.460 | 2 | 0 | 2813 | 92.482 | 0 | 0 |
| 1894 | LR-SURV | 9.140 | 2 | 0 | 1955 | 64.274 | 0 | 0 |
| 1895 | LR-SURV | 5.688 | 2 | 0 | 2209 | 72.625 | 0 | 0 |

| PatientID | Group | Age at diagnosis (months) | INSS Stage | *MYCN* | OS time (days) | EFS time (months) | OS | EFS |
|---|---|---|---|---|---|---|---|---|
| 1897 | LR-SURV | 92.844 | 2 | 0 | 2106 | 69.238 | 0 | 0 |
| 1902 | LR-SURV | 0.592 | 2 | 0 | 3313 | 108.921 | 0 | 0 |
| 1910 | LR-SURV | 36.066 | 2 | 0 | 1800 | 59.178 | 0 | 0 |
| 1944 | LR-SURV | 15.733 | 2 | 0 | 2883 | 16.167 | 0 | 1 |
| 1945 | LR-SURV | 15.267 | 2 | 0 | 1589 | 52.967 | 0 | 0 |
| 1946 | LR-SURV | 17.600 | 2 | 0 | 1794 | 59.800 | 0 | 0 |
| 1947 | LR-SURV | 3.033 | 2 | 0 | 1996 | 66.533 | 0 | 0 |
| 1948 | LR-SURV | 15.100 | 2 | 0 | 2230 | 21.700 | 0 | 1 |
| 1951 | LR-SURV | 12.700 | 2 | 0 | 2329 | 77.633 | 0 | 0 |
| 1952 | LR-SURV | 13.600 | 2 | 0 | 3898 | 129.933 | 0 | 0 |
| 1953 | LR-SURV | 23.833 | 2 | 0 | 1924 | 64.133 | 0 | 0 |
| 1954 | LR-SURV | 16.900 | 2 | 0 | 2041 | 68.033 | 0 | 0 |
| 1958 | LR-SURV | 21.267 | 2 | 0 | 2173 | 72.433 | 0 | 0 |
| 1962 | LR-SURV | 23.500 | 2 | 0 | 3388 | 112.933 | 0 | 0 |
| 1963 | LR-SURV | 8.000 | 2 | 0 | 1307 | 1.800 | 0 | 1 |
| 1966 | LR-SURV | 0.200 | 2 | 0 | 1508 | 6.400 | 0 | 1 |
| 1967 | LR-SURV | 12.467 | 2 | 0 | 2083 | 4.467 | 0 | 1 |
| 2084 | LR-SURV | 13.118 | 2 | 0 | 1279 | 42.049 | 0 | 0 |
| 2102 | LR-SURV | 92.844 | 2 | 0 | 2106 | 69.238 | 0 | 0 |
| 2103 | LR-SURV | 6.148 | 2 | 0 | 2793 | 91.825 | 0 | 0 |
| 2104 | LR-SURV | 0.592 | 2 | 0 | 3313 | 108.921 | 0 | 0 |
| 2118 | LR-SURV | 9.205 | 2 | 0 | 2191 | 72.033 | 0 | 0 |
| 2160 | LR-SURV | 0.592 | 2 | 0 | 1848 | 60.756 | 0 | 0 |
| 2660 | LR-SURV | 46.126 | 2 | 0 | 1243 | 23.934 | 0 | 1 |
| 2738 | LR-SURV | 4.175 | 2 | 0 | 4134 | 135.912 | 0 | 0 |
| 2743 | LR-SURV | 20.121 | 2 | 0 | 1417 | 46.586 | 0 | 0 |
| 2760 | LR-SURV | 13.808 | 2 | 0 | 1441 | 47.375 | 0 | 0 |
| 2765 | LR-SURV | 12.230 | 2 | 0 | 1269 | 41.721 | 0 | 0 |
| 2796 | LR-SURV | 8.252 | 2 | 0 | 1023 | 33.633 | 0 | 0 |
| 193 | LR-SURV | 7.627 | 3 | 0 | 2678 | 32.844 | 0 | 1 |
| 267 | LR-SURV | 1.249 | 3 | 0 | 2264 | 74.433 | 0 | 0 |
| 1178 | LR-SURV | 7.956 | 3 | 0 | 1727 | 56.778 | 0 | 0 |
| 1507 | LR-SURV | 6.279 | 3 | 0 | 2466 | 81.074 | 0 | 0 |
| 1513 | LR-SURV | 0.921 | 3 | 0 | 1265 | 41.589 | 0 | 0 |
| 1722 | LR-SURV | 7.759 | 3 | 0 | 1674 | 55.036 | 0 | 0 |
| 1761 | LR-SURV | 0.000 | 3 | 0 | 2462 | 80.942 | 0 | 0 |
| 1774 | LR-SURV | 0.658 | 3 | 0 | 1840 | 60.493 | 0 | 0 |
| 1777 | LR-SURV | 8.712 | 3 | 0 | 1941 | 18.345 | 0 | 1 |
| 1819 | LR-SURV | 6.279 | 3 | 0 | 1921 | 5.852 | 0 | 1 |
| 1832 | LR-SURV | 9.074 | 3 | 0 | 1805 | 59.342 | 0 | 0 |
| 1839 | LR-SURV | 9.436 | 3 | 0 | 1094 | 35.967 | 0 | 0 |
| 1846 | LR-SURV | 8.088 | 3 | 0 | 2904 | 34.389 | 0 | 1 |
| 1850 | LR-SURV | 8.712 | 3 | 0 | 1651 | 54.279 | 0 | 0 |
| 1861 | LR-SURV | 6.148 | 3 | 0 | 1907 | 62.696 | 0 | 0 |
| 1880 | LR-SURV | 0.822 | 3 | 0 | 1257 | 41.326 | 0 | 0 |
| 1881 | LR-SURV | 5.885 | 3 | 0 | 3011 | 98.992 | 0 | 0 |
| 1888 | LR-SURV | 4.077 | 3 | 0 | 2616 | 86.005 | 0 | 0 |
| 1889 | LR-SURV | 7.825 | 3 | 0 | 2766 | 90.937 | 0 | 0 |
| 1890 | LR-SURV | 7.003 | 3 | 0 | 1598 | 52.537 | 0 | 0 |
| 1956 | LR-SURV | 10.133 | 3 | 0 | 3072 | 3.267 | 0 | 1 |
| 1960 | LR-SURV | 7.6 | 3 | 0 | 2707 | 90.233 | 0 | 0 |
| 2114 | LR-SURV | 6.279 | 3 | 0 | 2466 | 81.074 | 0 | 0 |
| 2661 | LR-SURV | 1.512 | 3 | 0 | 1860 | 3.847 | 0 | 1 |
| 318 | | 3.123 | 4 | 0 | 2059 | 67.693 | 0 | 0 |
| 1179 | | 6.082 | 4 | 0 | 1340 | 44.055 | 0 | 0 |
| 1773 | | 3.616 | 4 | 0 | 2103 | 2.301 | 0 | 1 |
| 1792 | | 3.058 | 4 | 0 | 1814 | 1.742 | 0 | 1 |
| 1797 | | 2.860 | 4 | 0 | 1952 | 64.175 | 0 | 0 |
| 2067 | | 10.389 | 4 | 0 | 1378 | 45.304 | 0 | 0 |
| 2068 | | 10.389 | 4 | 0 | 1378 | 45.304 | 0 | 0 |
| 2085 | | 7.792 | 4 | 0 | 1988 | 1.874 | 0 | 1 |
| 2121 | | 11.573 | 4 | 0 | 2191 | 72.033 | 0 | 0 |
| 1780 | | 1.447 | 1 | 0 | 957 | 31.463 | 0 | 0 |
| 1785 | | 6.575 | 1 | 0 | 10 | 0.329 | 0 | 0 |

| PatientID | Group | Age at diagnosis (months) | INSS Stage | *MYCN* | OS time (days) | EFS time (months) | OS | EFS |
|---|---|---|---|---|---|---|---|---|
| 1791 | | 6.608 | 1 | 0 | 336 | 11.047 | 0 | 0 |
| 1794 | | 0.690 | 1 | 0 | 927 | 30.477 | 0 | 0 |
| 1796 | | 1.348 | 1 | 0 | 721 | 23.704 | 0 | 0 |
| 1810 | | 1.775 | 1 | 0 | 177 | 5.819 | 0 | 0 |
| 1811 | | 11.868 | 1 | 0 | 1 | 0.033 | 0 | 0 |
| 1812 | | 5.162 | 1 | 0 | 539 | 17.721 | 0 | 0 |
| 1825 | | 10.685 | 1 | 0 | 838 | 27.551 | 0 | 0 |
| 1826 | | 6.740 | 1 | 0 | 734 | 24.132 | 0 | 0 |
| 1835 | | 1.019 | 1 | 0 | 440 | 14.466 | 0 | 0 |
| 1836 | | 8.811 | 1 | 0 | 14 | 0.460 | 0 | 0 |
| 1840 | | 5.589 | 1 | 0 | 928 | 30.510 | 0 | 0 |
| 1854 | | 2.038 | 1 | 0 | 35 | 1.151 | 0 | 0 |
| 1858 | | 1.940 | 1 | 0 | 166 | 5.458 | 0 | 0 |
| 1860 | | 3.090 | 1 | 0 | 105 | 3.452 | 0 | 0 |
| 1884 | | 29.063 | 1 | 0 | 996 | 32.745 | 0 | 0 |
| 1934 | | 3.847 | 1 | 0 | 608 | 19.989 | 0 | 0 |
| 2089 | | 1.052 | 1 | 0 | 855 | 28.110 | 0 | 0 |
| 1702 | | 6.900 | 2 | 0 | 1820 | 22.467 | 1 | 1 |
| 1793 | | 9.962 | 2 | 0 | 189 | 6.214 | 0 | 0 |
| 1795 | | 1.874 | 2 | 0 | 317 | 0.756 | 1 | 1 |
| 1813 | | 4.340 | 2 | 0 | 154 | 5.063 | 0 | 0 |
| 1848 | | 4.110 | 2 | 0 | 775 | 25.479 | 0 | 0 |
| 1935 | | 6.378 | 2 | 0 | 698 | 22.948 | 0 | 0 |
| 1937 | | 10.521 | 2 | 0 | 0 | 0.000 | 0 | 0 |
| 1957 | | 152.067 | 2 | 0 | 1773 | 25.300 | 1 | 1 |
| 520 | | 156.986 | 3 | 0 | 1499 | 36.658 | 0 | 1 |
| 1516 | | 43.923 | 3 | 0 | 298 | 9.797 | 0 | 0 |
| 1760 | | 27.649 | 3 | 0 | 1806 | 59.375 | 0 | 0 |
| 1772 | | 26.104 | 3 | 0 | 1547 | 50.860 | 0 | 0 |
| 1775 | | 13.710 | 3 | 0 | 1064 | 34.981 | 0 | 0 |
| 1781 | | 6.575 | 3 | 0 | 421 | 13.841 | 0 | 0 |
| 1802 | | 8.351 | 3 | 0 | 366 | 12.033 | 0 | 0 |
| 1805 | | 23.178 | 3 | 0 | 922 | 30.312 | 0 | 0 |
| 1816 | | 1.019 | 3 | 0 | 637 | 20.942 | 0 | 0 |
| 1818 | | 9.468 | 3 | 0 | 339 | 11.145 | 0 | 0 |
| 1859 | | 4.767 | 3 | 0 | 760 | 24.986 | 0 | 0 |
| 1887 | | 27.715 | 3 | 0 | 4184 | 137.556 | 0 | 0 |
| 1949 | | 37.533 | 3 | 0 | 1998 | 41.533 | 0 | 1 |
| 1955 | | 94.367 | 3 | 0 | 2286 | 22.833 | 0 | 1 |
| 2069 | | 13.184 | 3 | 0 | 2069 | 37.644 | 0 | 1 |
| 2070 | | 21.337 | 3 | 0 | 2444 | 80.351 | 0 | 0 |
| 2098 | | 18.641 | 3 | 0 | 3196 | 105.074 | 0 | 0 |
| 2666 | | 22.356 | 3 | 0 | 1296 | 42.608 | 0 | 0 |
| 2704 | | 47.605 | 3 | 0 | 1758 | 57.797 | 0 | 0 |
| 2705 | | 44.548 | 3 | 0 | 1798 | 59.112 | 0 | 0 |
| 2706 | | 19.200 | 3 | 0 | 1286 | 42.279 | 0 | 0 |
| 2742 | | 55.233 | 3 | 0 | 1398 | 45.962 | 0 | 0 |
| 2745 | | 55.496 | 3 | 0 | 1442 | 47.408 | 0 | 0 |
| 2746 | | 38.860 | 3 | 0 | 1063 | 34.948 | 0 | 0 |
| 2748 | | 16.603 | 3 | 0 | 1060 | 34.849 | 0 | 0 |
| 2754 | | 28.274 | 3 | 0 | | | | 0 |
| 2755 | | 110.301 | 3 | 0 | 1198 | 30.444 | | 1 |
| 2759 | | 57.995 | 3 | 0 | 1247 | 40.997 | 0 | 0 |
| 2761 | | 23.507 | 3 | 0 | 709 | 23.310 | 0 | 0 |
| 2764 | | 19.463 | 3 | 0 | 1100 | 36.164 | 0 | 0 |
| 2793 | | 1.677 | 3 | 0 | 142 | 2.762 | | 1 |
| 1803 | | 52.932 | 4 | 0 | 259 | 8.515 | 0 | 0 |
| 2778 | | 36.953 | 4 | 0 | 264 | | 1 | 1 |
| 2782 | | 32.318 | 4 | 0 | 344 | 11.310 | 0 | 0 |
| 764 | | 26.038 | 3 | 0 | 1006 | 33.074 | 0 | 0 |
| 323 | | 8.482 | 4s | 0 | 1567 | 51.518 | 0 | 0 |
| 330 | | 3.682 | 4s | 0 | 2207 | 72.559 | 0 | 0 |
| 1495 | | 1.019 | 4s | 0 | 5494 | 180.625 | 0 | 0 |
| 1508 | | 1.019 | 4s | 0 | 36 | 1.052 | 1 | 1 |

SIOPEN clinical annotation

| PatientID | Group | Age at diagnosis (months) | INSS Stage | *MYCN* | OS time (days) | EFS time (months) | OS | EFS |
|---|---|---|---|---|---|---|---|---|
| 1510 | | 0.789 | 4s | 0 | 1461 | 1.940 | 0 | 1 |
| 1511 | | 6.345 | 4s | 0 | 1461 | 48.033 | 0 | 0 |
| 1512 | | 1.644 | 4s | 0 | 1461 | 48.033 | 0 | 0 |
| 1514 | | 7.036 | 4s | 0 | 1091 | 35.868 | 0 | 0 |
| 1717 | | 3.485 | 4s | 0 | 1911 | 62.827 | 0 | 0 |
| 1765 | | 1.874 | 4s | 0 | 3777 | 124.175 | 0 | 0 |
| 1771 | | 9.995 | 4s | 0 | 3454 | 48.986 | 0 | 1 |
| 1801 | | 0.855 | 4s | 0 | 394 | 12.953 | 0 | 0 |
| 1806 | | 1.085 | 4s | 0 | 1590 | 52.274 | 0 | 0 |
| 1830 | | 0.099 | 4s | 0 | 2039 | 67.036 | 0 | 0 |
| 1842 | | 0.460 | 4s | 0 | 1503 | 49.414 | 0 | 0 |
| 1844 | | 0.296 | 4s | 0 | 1562 | 51.353 | 0 | 0 |
| 1921 | | 2.532 | 4s | 0 | 2192 | 72.066 | 0 | 0 |
| 1922 | | 8.811 | 4s | 0 | 2009 | 66.049 | 0 | 0 |
| 1928 | | 4.405 | 4s | 0 | 1461 | 2.860 | 0 | 1 |
| 2021 | | 7.101 | 4s | 0 | 1092 | 35.901 | 0 | 0 |
| 2022 | | 7.101 | 4s | 0 | 1092 | 35.901 | 0 | 0 |
| 2086 | | 6.181 | 4s | 0 | 2518 | 82.784 | 0 | 0 |
| 2087 | | 3.649 | 4s | 0 | 2497 | 82.093 | 0 | 0 |
| 2112 | | 5.162 | 4s | 0 | 2575 | 84.658 | 0 | 0 |
| 2115 | | 8.351 | 4s | 0 | 2539 | 83.474 | 0 | 0 |
| 2116 | | 0.888 | 4s | 0 | 2192 | 72.066 | 0 | 0 |
| 2159 | | 2.893 | 4s | 0 | 2178 | 71.605 | 0 | 0 |
| 2166 | | 4.405 | 4s | 0 | 1461 | 2.860 | 0 | 1 |
| 2168 | | 11.605 | 4s | 0 | 1469 | 48.296 | 0 | 0 |
| 2713 | | 11.375 | 4s | 0 | 2182 | 10.751 | 0 | 1 |
| 2715 | | 5.852 | 4s | 0 | 1770 | 3.912 | 0 | 1 |
| 2757 | | 0.263 | 4s | 0 | 1909 | 7.036 | 0 | 1 |
| 2763 | | 7.397 | 4s | 0 | 1940 | 3.551 | 0 | 1 |
| 334 | | 6.247 | 1 | 1 | 2190 | 72.000 | 0 | 0 |
| 1751 | | 26.071 | 1 | 1 | 760 | 22.521 | 1 | 1 |
| 1938 | | 24.526 | 2 | 1 | 641 | 21.074 | 0 | 0 |
| 1136 | | 22.323 | 3 | 1 | 862 | 28.340 | 0 | 0 |
| 1687 | | 31.496 | 3 | 1 | 822 | 27.025 | 0 | 0 |
| 1834 | | 23.638 | 3 | 1 | 205 | 6.740 | | 0 |
| 1786 | | 17.918 | 4 | 1 | 125 | 4.110 | | 0 |
| 2785 | | 18.838 | 4 | 1 | 524 | 17.227 | 0 | 0 |
| 2786 | | 21.830 | 4 | 1 | 576 | 18.937 | 0 | 0 |
| 1837 | | 0.559 | 4s | 1 | 2178 | 71.605 | 0 | 0 |
| 1933 | | 10.455 | 1 | | 500 | 16.438 | 0 | 0 |
| 1849 | | 10.060 | 2 | | 2136 | 43.068 | 0 | 0 |
| 2774 | | 7.463 | 2 | | 1461 | 14.104 | 0 | 1 |
| 1856 | | 10.652 | 4 | | 295 | 8.679 | | 1 |
| 1931 | | 48.822 | 4 | | 1049 | 34.488 | 0 | 0 |
| 1932 | | 23.408 | 4 | | 1137 | 30.970 | 0 | 1 |
| 2783 | | 26.137 | 4 | | 484 | 15.912 | 0 | 0 |

SIOPEN clinical annotation

| Characteristic | Classes | Count (percentage) |
|---|---|---|
| **Risk Classification** | HR-DOD | 46/245 (19%) |
| | HR-SURV | 29/245 (12%) |
| | LR-SURV | 170/245 (69%) |
| **INSS Stage** | 1 | 115/366 (31%) |
| | 2 | 69/366 (20%) |
| | 3 | 66/366 (18%) |
| | 4 | 82/366 (22%) |
| | 4S | 34/366 (9%) |
| ***MYCN*** | Not amplified | 307/357 (86%) |
| | Amplified | 50/357 (14%) |
| **Age** | Age at diagnosis > 12 months | 191/366 (52%) |
| | Age at diagnosis < 12 months | 175/366 (48%) |

SIOPEN overview annotation

| Name | Forward primer | Reverse primer | cDNA location | Genomic location |
|---|---|---|---|---|
| *CNR1* | ACCAGGGTTTAGTTTGCATT | GAGCTCTGAAACATGTGGAT | NM_001160226:4107-4189 | chr6: 88851256- 88851338 |
| *GRB10* | TCCCAGAACAGATGGTTACT | AGGACAACTACTGGAGTTCA | NM_001001550:895-984 | chr7:50684018-50685829 |
| *KRT19* | CGACTACAGCCACTACTACA | AACTTGGTTCGGAAGTCATC | NM_002276:516-648 | chr17:39681440-39684125 |
| *PRPH* | CATCCCTTTCCTGGCTTATG | GCACATAGGTCACATCAAGT | NM_006262:1601-1689 | chr12:49692250-49692337 |
| *QPCT* | GGAACTTGCTCGTGCCTTAG | TCTGGCTTGGAGTCTGAAACA | NM_012413:625-691 | chr2:37586959-37594397 |

SIOPEN assays

| PatientID | Type | Reference *AluSq* | Reference *HMBS* | Reference *HPRT1* | Reference *SDHA* | Reference *UBC* | *CNR1* | *GRB10* | *KRT19* | *PRPH* | *QPCT* |
|---|---|---|---|---|---|---|---|---|---|---|---|
| 194 | HR-DOD | 0.854245248 | 0.65747771 | 1.312160725 | 1.834608491 | 0.739615427 | 0.116862899 | 0.246732035 | NaN | 0.000428003 | 0.27763199 |
| 1500 | HR-DOD | 1.466850888 | 0.568413959 | 2.253151691 | 1.010782241 | 0.526624747 | 1.650483698 | 1.54865625 | 6.968822101 | 0.888786674 | 0.940334343 |
| 1501 | HR-DOD | 0.445878402 | 0.653841969 | 1.858249789 | 3.357697408 | 0.549749854 | 0.340302319 | 0.612599863 | 1.83135925 | 0.161758014 | 0.339914949 |
| 1503 | HR-DOD | 1.1588728 | 0.84382392 | 2.00269739 | 1.759874099 | 0.290145251 | 0.83098186 | 2.772172339 | 1.415110613 | 0.655155417 | 0.248487591 |
| 1716 | HR-DOD | 0.992213315 | 1.15750136 | 0.502760929 | 1.75500145 | 0.986812039 | 0.275264199 | 0.149375656 | 0.068721952 | 0.006688549 | 0.106376082 |
| 1852 | HR-DOD | 0.690983048 | 1.273689737 | 1.873770737 | 0.985871979 | 0.615080533 | 0.408070094 | 0.396396725 | 35.38129201 | 3.565017333 | 31.23833629 |
| 2164 | HR-DOD | 1.885206058 | 0.269249186 | 1.950635806 | 1.57731655 | 0.64031212 | 0.131659989 | 2.519289557 | 0.09827136 | 4.999934138 | 1.378639938 |
| 2710 | HR-DOD | 0.54439368 | 1.18510418 | 4.118001754 | 0.184983978 | 2.034743899 | 0.017860161 | 0.134066152 | 45.91567427 | 3.506201941 | 0.230245636 |
| 2711 | HR-DOD | 0.491315692 | 1.271925252 | 1.369780231 | 0.165794979 | 7.046208778 | NaN | 0.020376032 | 0.038819217 | 1.560392632 | 2.383771109 |
| 2712 | HR-DOD | 0.886825539 | 0.817343197 | 10.89764462 | 0.181680089 | 0.696814824 | 0.077852592 | 1.305875129 | 6.083555843 | 9.087661456 | 2.819121611 |
| 2714 | HR-DOD | 0.86377186 | 0.727497345 | 1.092735659 | 0.208985133 | 6.968495552 | 0.913127922 | 1.523106633 | 1.22851445 | 0.815586099 | 0.405351592 |
| 2719 | HR-DOD | 0.330042158 | 0.08857319 | 0.441334503 | 10.0107088 | 7.742756233 | 0.000740552 | 0.062891853 | 0.992345787 | 3.50134468 | 0.432042671 |
| 2752 | HR-DOD | 5.818802269 | 0.302082369 | 1.365987654 | 1.052246144 | 0.395801016 | 11.63894905 | 1.483512362 | 12.37121101 | 4.34064396 | 0.186758487 |
| 2771 | HR-DOD | | | no Cq value for at least 1 reference gene | | | NaN | NaN | NaN | NaN | NaN |
| 2772 | HR-DOD | | | no Cq value for at least 1 reference gene | | | NaN | NaN | NaN | NaN | NaN |
| 2775 | HR-DOD | 0.966420004 | 1.250941123 | 1.172789786 | 0.643259259 | 1.096455471 | 0.200390898 | 1.71596122 | 7.775388849 | 0.561714555 | 0.524584001 |
| 2780 | HR-DOD | 1.569953928 | 0.227354252 | 0.611296598 | 0.890984489 | 5.143845185 | 1.206550992 | 2.279973331 | 15.5507777 | 1.441838014 | 0.389378657 |
| 2788 | HR-DOD | 12.93064398 | 0.003947114 | 5.433735891 | 0.886057496 | 4.069487797 | 0.961186898 | 0.895656192 | NaN | 7.727003711 | NaN |
| 1536 | HR-DOD | 0.844823706 | 1.048999426 | 1.161464005 | 1.623910613 | 0.598261124 | 0.788339457 | 0.739702445 | 0.213886129 | 0.138111857 | 0.234107931 |
| 1822 | HR-DOD | 0.768822372 | 5.180221865 | 1.109526745 | 0.315010587 | 0.718394008 | 0.318836541 | 0.152725932 | NaN | 0.05863513 | 0.363303134 |
| 1908 | HR-DOD | 0.594901294 | 1.708835933 | 0.687744425 | 1.096930577 | 1.303912647 | 0.620235879 | 0.71154009 | 2.156833525 | 0.049649021 | 0.243036637 |
| 2090 | HR-DOD | 0.525121155 | 1.627902042 | 1.693425413 | 1.844809979 | 0.374450578 | 0.607471609 | 1.890826447 | 0.013974252 | 0.1301195 | 0.149606599 |
| 111 | HR-DOD | 0.538389516 | 1.36510686 | 1.185868833 | 1.109163635 | 1.034438031 | 1.011775275 | 1.563757658 | NaN | 0.88509799 | 1.489931201 |
| 127 | HR-DOD | 0.730381547 | 1.890822436 | 1.025227757 | 1.64657956 | 0.428939839 | 0.036068641 | 0.436791579 | 0.002810251 | 0.255945597 | 0.203802791 |
| 772 | HR-DOD | 0.373381466 | 3.657895573 | 1.272745923 | 1.207038942 | 0.476598799 | 0.060324543 | 0.488698625 | 0.112415652 | 0.122930013 | 0.290628021 |
| 939 | HR-DOD | 0.48927661 | 0.877221994 | 0.85142092 | 1.18219844 | 2.314735296 | 1.600906321 | 2.153998058 | 0.022986248 | 0.538086155 | 0.280728164 |
| 1231 | HR-DOD | 0.686210079 | 0.800522517 | 0.338199034 | 0.414508107 | 12.98565807 | 0.083440342 | 0.375014153 | NaN | 0.14199471 | 0.159900429 |
| 1496 | HR-DOD | 2.29218299 | 2.905958075 | 2.152397363 | 0.706848149 | 0.098676286 | 0.13368309 | 0.729518743 | 0.149158162 | 0.55244743 | 0.259759013 |
| 1498 | HR-DOD | 2.132760878 | 1.652916549 | 2.812678087 | 1.459497988 | 0.069100848 | 9.599031748 | 0.466845457 | 4.661889557 | 0.002197241 | 2.464415841 |
| 1499 | HR-DOD | 3.318939249 | 0.775400671 | 1.670111525 | 1.832066946 | 0.126995319 | 0.392533827 | 0.069300854 | 0.01627628 | 0.244500461 | 0.044787679 |
| 1515 | HR-DOD | 0.616734827 | 1.531572742 | 1.39662475 | 1.459497988 | 0.519374548 | 0.291767054 | 1.240581872 | 0.071342957 | 0.309907396 | 0.559126146 |
| 1758 | HR-DOD | 2.34684445 | 2.193164291 | 0.888808454 | 0.791945043 | 0.27602048 | 0.22701962 | 0.026260113 | 0.000719317 | 0.066426794 | 0.176684295 |
| 1776 | HR-DOD | 1.120950229 | 1.211684325 | 2.047614949 | 0.224918186 | 1.59864236 | 0.572318001 | 1.66211125 | NaN | 1.036635777 | 0.323815896 |
| 1784 | HR-DOD | 1.53977785 | 3.543104186 | 1.386977558 | 0.338088353 | 0.390893425 | 1.436829513 | 0.763667721 | 0.74273261 | 0.171931893 | 0.146731011 |
| 1788 | HR-DOD | 0.613324387 | 1.713580406 | 0.744293966 | 0.822149249 | 1.5549274 | 0.015355389 | 0.052302255 | 0.059660291 | 0.507651061 | 0.240356165 |
| 1820 | HR-DOD | 0.541383274 | 1.41128943 | 0.552460968 | 1.062506909 | 2.229696306 | 0.29831096 | 0.339861053 | NaN | 0.679200342 | 0.274189592 |
| 1827 | HR-DOD | 0.624477814 | 3.370619767 | 1.064329196 | 1.225586425 | 0.364211207 | 0.230188695 | 0.438004303 | 0.000424754 | 0.618956142 | 0.055523631 |
| 1924 | HR-DOD | 0.257871139 | 2.491498817 | 0.814476198 | 2.024368909 | 0.943992447 | NaN | 2.681456685 | NaN | NaN | NaN |
| 1936 | HR-DOD | 4.283298503 | 1.240579241 | 0.569569755 | 1.933845076 | 0.170855337 | 0.636789693 | 1.203317163 | 0.033653837 | 1.847928697 | 2.633982416 |
| 2015 | HR-DOD | 0.594901294 | 1.662107724 | 0.687744425 | 4.037527881 | 0.364211207 | 0.022890438 | 1.984832068 | NaN | 0.083499368 | 0.161459666 |
| 2659 | HR-DOD | 0.524393687 | 0.628948541 | 1.392757847 | 2.252414311 | 0.966503741 | 0.011116827 | 0.658391196 | NaN | 0.046905766 | 0.081178738 |
| 2709 | HR-DOD | | | no Cq value for at least 1 reference gene | | | NaN | NaN | NaN | NaN | NaN |
| 2768 | HR-DOD | 2.845578979 | 0.406411714 | 0.118088833 | 3.583755521 | 2.043223782 | 0.857904455 | 0.386092102 | 0.030330536 | 0.012568089 | 0.050668949 |
| 2784 | HR-DOD | 3.219244655 | 0.623738791 | 0.342920117 | 0.795245508 | 1.826203099 | 1.450840465 | 0.257923825 | NaN | 0.282027762 | 0.019987554 |
| 2769 | HR-DOD | 1.383883236 | 0.940183254 | 0.90622704 | 0.551515074 | 1.537777976 | 5.09431754 | 0.127362896 | 0.71544602 | 0.653341456 | 3.046696337 |
| 2777 | HR-DOD | 1.645723791 | 1.050454657 | 0.85142092 | 0.486824008 | 1.39556297 | 0.222039598 | 2.615373423 | 1.701626996 | 0.364985003 | 0.082884475 |
| 1497 | HR-SURV | 0.538389516 | 0.550576099 | 2.840105348 | 0.864221018 | 1.374443138 | 1.666758056 | 0.84382571 | 0.530312561 | 2.119771989 | 1.031858907 |
| 1517 | HR-SURV | 0.581045168 | 0.392024001 | 1.71231063 | 1.384602849 | 1.851695945 | 3.882238872 | 2.106746822 | 4.773061173 | 1.9778172 | 2.540734656 |
| 1677 | HR-SURV | 0.697721029 | 0.738676143 | 2.496564958 | 0.634403318 | 1.225055595 | 0.050097725 | 2.806976429 | NaN | 0.026791313 | 0.985717206 |
| 1829 | HR-SURV | 0.954437228 | 0.750026715 | 1.767786987 | 0.265258745 | 2.979039437 | 9.868897263 | 1.302259489 | 3.140327071 | 5.578623126 | 4.963114417 |
| 2016 | HR-SURV | 0.843653343 | 0.07573027 | 1.488587931 | 6.265684915 | 1.678121556 | 1.105645497 | 0.909418936 | 0.014407011 | 0.90244386 | 2.270870937 |
| 2017 | HR-SURV | 1.287633684 | 0.315784856 | 1.541082871 | 1.026314023 | 1.5549274 | 0.560539867 | 3.898764327 | 0.001155646 | 1.38694357 | 3.0382608 |
| 2092 | HR-SURV | 0.878261351 | 0.34508266 | 1.10339125 | 1.597119456 | 1.872346208 | 0.743749598 | 0.829904335 | 3.118635248 | 1.214118376 | 0.030846395 |
| 2093 | HR-SURV | 0.799251927 | 1.71595758 | 0.880225117 | 0.456748603 | 1.813588593 | 0.662911823 | 1.47940489 | 10.88987 | 3.372711908 | 0.849829727 |
| 2171 | HR-SURV | 1.191453149 | 0.885776046 | 1.638010318 | 3.261356661 | 0.177371635 | 0.571525149 | 1.05776639 | 6.280654143 | 1.91840735 | 0.368885686 |
| 2172 | HR-SURV | 2.195762813 | 1.15429653 | 2.701844124 | 0.345671178 | 0.422448454 | 22.39164926 | 6.466642922 | 0.171100375 | 4.263118302 | 1.05208095 |
| 2718 | HR-SURV | 0.479207443 | 0.251915865 | 0.399964662 | 12.91944529 | 1.603080888 | 3.407913905 | 0.211249078 | 0.113984916 | 1.279791767 | 0.422565156 |
| 2758 | HR-SURV | 2.470361583 | 0.265542353 | 0.451858963 | 0.696151022 | 4.846177417 | 0.235677982 | 0.474019051 | 0.069971674 | 0.908720853 | 0.415593707 |
| 2762 | HR-SURV | 1.900952156 | 8.050140087 | 0.319955782 | 0.747151246 | 0.273354915 | 1.914395471 | 2.453798782 | 0.304584583 | 4.038750687 | 0.209532467 |
| 2776 | HR-SURV | 1.082766549 | 1.811285403 | 0.666161759 | 0.950970321 | 0.804880898 | 4.213119652 | 1.61666073 | 2.893698929 | 1.182556481 | 0.079287965 |
| 2781 | HR-SURV | 1.0782728 | 0.768977819 | 0.360468337 | 1.933845076 | 1.730090159 | 0.53547417 | 1.749590084 | 0.11163914 | 1.08365768 | 0.468865397 |
| 1540 | HR-SURV | 1.393508905 | 0.980108851 | 1.147062852 | 0.774572519 | 0.824074813 | 0.160304729 | 0.237667567 | 0.017786357 | 0.053508396 | 0.399770997 |
| 1766 | HR-SURV | 0.937390593 | 1.34631303 | 2.152397363 | 1.222193084 | 0.30121118 | 1.146223904 | 1.017491646 | 0.156573811 | 0.497893502 | 1.252877806 |
| 1815 | HR-SURV | 1.076779032 | 1.204983915 | 1.161464005 | 0.741990295 | 0.894310192 | 8.379643622 | 1.017491646 | 5.080303991 | 4.704068573 | 1.177107201 |
| 2162 | HR-SURV | 3.028749418 | 0.829902575 | 1.085187582 | 0.865419913 | 0.423621355 | 2.376583167 | 7.819177992 | 0.92460869 | 0.203896525 | 104.491785 |
| 1686 | HR-SURV | 0.363675142 | 1.75687785 | 1.006916775 | 2.318950817 | 0.670285311 | 0.23340201 | 0.223603852 | 0.002798588 | 0.18658554 | 0.233136325 |
| 1824 | HR-SURV | 1.072310133 | 5.437765317 | 1.148654122 | 0.191242122 | 0.780703814 | 0.178115983 | 0.159211555 | NaN | 0.001286716 | 0.07326387 |
| 2770 | HR-SURV | | | no Cq value for at least 1 reference gene | | | NaN | NaN | NaN | NaN | NaN |
| 1759 | HR-SURV | 1.211439386 | 2.217622636 | 0.766280471 | 0.641478235 | 0.757252963 | 0.428357986 | 0.070561104 | 0.005429048 | 0.160863519 | 0.347538741 |
| 1821 | HR-SURV | 1.725150495 | 5.422709512 | 0.671725845 | 0.489531032 | 0.32507561 | 1.040220149 | 0.250870884 | 0.060577037 | 0.054936511 | 0.488099004 |
| 1823 | HR-SURV | 1.311049559 | 0.831053862 | 0.450607879 | 0.415658962 | 4.900222379 | NaN | 0.125783706 | NaN | 0.01695564 | 2.53018998 |
| 1853 | HR-SURV | | | no Cq value for at least 1 reference gene | | | NaN | NaN | NaN | NaN | NaN |
| 2091 | HR-SURV | 0.548940703 | 0.710552862 | 0.560173035 | 3.93802487 | 1.162191028 | 0.621957924 | 0.55594611 | 0.051222264 | 0.230351531 | 0.263020238 |
| 2530 | HR-SURV | 0.780636174 | 1.885587224 | 0.785642262 | 0.1298999 | 6.656884893 | 0.536960879 | 0.183139761 | NaN | 0.015842119 | 0.016302598 |
| 1542 | HR-SURV | | | no Cq value for at least 1 reference gene | | | NaN | NaN | NaN | NaN | NaN |
| 116 | LR-SURV | 0.574636772 | 1.277226055 | 1.189161322 | 1.03775956 | 1.104081921 | 0.993704559 | 1.139986665 | 0.080153805 | 0.019342379 | 1.944293092 |
| 126 | LR-SURV | 1.218175692 | 0.622874704 | 0.854969254 | 1.502611681 | 1.025869503 | 0.781809429 | 0.501046689 | 1.539983429 | 0.074941522 | 1.870268905 |
| 130 | LR-SURV | 1.1588728 | 1.113432337 | 1.496865344 | 0.867822696 | 0.596604689 | 1.446823453 | 1.007665599 | 2.714929672 | 3.010303799 | 1.275661639 |
| 266 | LR-SURV | 0.474579676 | 1.632421808 | 1.059911967 | 1.373133811 | 0.886902379 | 2.257754919 | 1.10421182 | 3.688194722 | 2.398128709 | 0.708699391 |
| 315 | LR-SURV | 0.517174204 | 1.102679779 | 0.794403808 | 2.190821923 | 1.007547058 | 3.602226478 | 0.509451583 | 12.87869785 | 5.373675079 | 6.009503136 |
| 320 | LR-SURV | 0.405766656 | 2.850102666 | 1.155041305 | 1.311731299 | 0.570716913 | 0.919479228 | 0.419579748 | 1.203231988 | 0.468431205 | 0.495599183 |
| 321 | LR-SURV | 0.421826671 | 1.353799312 | 1.067284238 | 1.941904459 | 0.844896907 | 3.041723235 | 1.304066056 | 3.537952862 | 0.220967958 | 0.464337504 |
| 338 | LR-SURV | 4.224329067 | 0.742783611 | 0.85142092 | 0.966922538 | 0.387118517 | 4.819518064 | 0.977397249 | 1.643663334 | 0.505544183 | 1.107453186 |
| 346 | LR-SURV | 0.818311608 | 0.72850657 | 1.274511547 | 1.721268574 | 0.764637162 | 0.660160575 | 2.054827019 | 6.100446446 | 2.110974411 | 2.264583467 |
| 769 | LR-SURV | 0.510761689 | 0.981468512 | 1.365987654 | 1.498451327 | 0.97457642 | 6.280541226 | 3.552948924 | 1.680528287 | 3.006133524 | 1.557514629 |
| 824 | LR-SURV | 1.001888682 | 1.038869104 | 0.967239082 | 2.035625575 | 0.487964203 | 0.59167994 | 0.532560358 | 1.007594273 | 0.731996721 | 1.5705237 |
| 827 | LR-SURV | 1.710860609 | 0.070658841 | 2.997883758 | 1.702284559 | 1.620958575 | 2.211290995 | 2.03216416 | 3.122961592 | 1.637970178 | 16.60154626 |
| 1538 | LR-SURV | 0.627950273 | 0.790596728 | 1.187513936 | 3.141540594 | 0.5399311 | 1.256045641 | 1.114979318 | 0.549775616 | 1.244795794 | 1.666990306 |
| 1539 | LR-SURV | 1.001888682 | 0.613448394 | 1.435889216 | 1.061034982 | 1.067952309 | 2.383181598 | 1.395727161 | 1.506201541 | 1.547467479 | 2.715552423 |
| 1541 | LR-SURV | 2.596777172 | 0.586016783 | 1.185868833 | 1.230694109 | 0.450265305 | 1.401419513 | 0.424847491 | 0.125426563 | 0.797694973 | 1.270367328 |
| 1693 | LR-SURV | 1.587462181 | 0.692081521 | 1.748289916 | 0.882380138 | 0.590024686 | 0.649269295 | 1.925211504 | 1.808651973 | 1.200727763 | 1.193539005 |
| 1694 | LR-SURV | 1.567779018 | 0.664809954 | 1.026650011 | 2.044109133 | 0.457184128 | 6.863235642 | 1.122734612 | 0.810517267 | 0.760968738 | 2.440166662 |
| 1698 | LR-SURV | 1.412961565 | 0.628948541 | 1.155041305 | 1.435419298 | 0.678700613 | 1.23875329 | 1.63242527 | 1.237059443 | 0.534369322 | 0.90578824 |
| 1699 | LR-SURV | 1.589664397 | 0.607524245 | 1.229391525 | 1.465580509 | 0.574686563 | 4.496769357 | 1.040312488 | 1.888073367 | 2.064666965 | 1.055001986 |
| 1700 | LR-SURV | 1.434673275 | 1.04464582 | 1.972389454 | 0.98860919 | 0.342184715 | 0.993704559 | 1.163940209 | 0.358219264 | 1.825015973 | 0.888378088 |
| 1701 | LR-SURV | 0.720326169 | 0.869955728 | 3.448441384 | 0.574139103 | 0.805997474 | 1.737350561 | 2.257955336 | 1.324010741 | 0.744275727 | 3.042475645 |
| 1720 | LR-SURV | 0.464812967 | 1.162325297 | 1.519866328 | 2.169664875 | 0.561301267 | 1.618759757 | 3.25581099 | 6.075128087 | 2.793181052 | 2.255184886 |
| 1752 | LR-SURV | 0.495419384 | 1.355677378 | 1.553954696 | 0.90718722 | 1.056173773 | 0.042893085 | 0.680665055 | 1.796158701 | 2.956538555 | 0.680772995 |
| 1767 | LR-SURV | 1.531263138 | 0.224846743 | 0.8088502 | 1.382684712 | 2.597002175 | 0.891859829 | 0.73357529 | 0.157444452 | 1.406304581 | 1.167356905 |
| 1789 | LR-SURV | 0.609087846 | 1.033124334 | 1.128138694 | 1.099976134 | 1.280624239 | 2.006788936 | 1.627905495 | 3.188581654 | 0.616387321 | 3.394609714 |
| 1790 | LR-SURV | 0.646499976 | 1.378418423 | 0.742233203 | 1.260042349 | 1.199844315 | NaN | 0.36324553 | NaN | 2.02498201 | 0.414443033 |
| 1798 | LR-SURV | 0.449602581 | 2.327875479 | 1.324955994 | 0.275375521 | 2.618693518 | 2.90168093 | 2.321435101 | 1.278910075 | 0.982077709 | 0.879798907 |
| 1799 | LR-SURV | 2.487544307 | 0.498966163 | 1.6981271 | 0.649531871 | 0.730444833 | 5.010271867 | 15.38035555 | 0.915679625 | 0.470383412 | 1.949691297 |
| 1804 | LR-SURV | 1.620818136 | 1.998633427 | 0.359968968 | 1.006587245 | 0.851953871 | 9.625682844 | 0.592553347 | 1.209922656 | 6.207055694 | 0.690276232 |
| 1828 | LR-SURV | 0.65552478 | 8.299439391 | 0.602880698 | 0.733806822 | 0.41547893 | 1.742174203 | 0.668508101 | 1.578894556 | 0.672641218 | 0.800651163 |
| 1831 | LR-SURV | 0.851880058 | 1.311314638 | 1.577832921 | 0.518160958 | 1.094936514 | 2.787339798 | 0.931105759 | 1.786226247 | 3.879603167 | 1.019064745 |
| 1841 | LR-SURV | 1.060483535 | 2.707598624 | 1.184226009 | 0.269334738 | 1.09190491 | 3.351690394 | 2.95471084 | 8.356592734 | 3.186359596 | 3.211496478 |
| 1857 | LR-SURV | 0.509347519 | 0.482638159 | 0.868106798 | 3.203107511 | 1.462916293 | 2.406420711 | 1.389934549 | 7.971851448 | 2.81651097 | 1.699659453 |
| 1879 | LR-SURV | 0.38869817 | 1.048999426 | 1.153441187 | 1.814374441 | 1.171898176 | 0.336082864 | 3.375302838 | 0.01567832 | 0.107611867 | 0.885918392 |
| 1882 | LR-SURV | 1.426739774 | 0.724478052 | 0.486983381 | 2.780746483 | 0.71442141 | NaN | 1.028838795 | 1.05038253 | 1.75310097 | 0.973495162 |
| 1883 | LR-SURV | 0.790436911 | 1.313133767 | 0.768408 | 1.225586425 | 1.023029128 | 1.415085171 | 2.409972943 | NaN | 0.811076045 | 1.300661651 |
| 1886 | LR-SURV | 2.557478696 | 0.352822357 | 0.718940581 | 0.573343729 | 2.688585259 | 13.13085441 | 2.795326784 | 6.46618519 | 6.284984083 | 1.3316784 |
| 1891 | LR-SURV | 1.867000381 | 0.748987678 | 0.888808454 | 1.089353524 | 0.738590813 | 0.847267179 | 4.025078581 | 4.496850203 | 1.012491005 | 1.669302847 |
| 1892 | LR-SURV | 1.368620272 | 1.160715089 | 0.473666795 | 0.704891064 | 1.885369405 | 0.764659245 | 1.791312385 | 0.001091793 | 1.028049044 | 0.3256165 |
| 1893 | LR-SURV | 4.859211481 | 0.559811834 | 0.517612529 | 1.398104524 | 0.507981275 | 0.203188251 | 1.739915146 | NaN | 1.33598989 | NaN |
| 1896 | LR-SURV | 0.688115298 | 1.446948816 | 0.650645872 | 1.832066946 | 0.8425576 | 1.730140127 | 0.913208979 | 4.254279521 | 4.251314799 | 1.013429489 |
| 1898 | LR-SURV | 2.07444042 | 0.484649574 | 0.293604186 | 1.664942348 | 2.034743899 | 0.306272744 | 26.92765978 | 1.173578891 | 0.026276357 | 2.910457023 |
| 1899 | LR-SURV | 0.880699787 | 1.525216337 | 0.705121827 | 0.49981712 | 2.112347547 | 1.798618096 | 0.83799698 | 4.155191093 | 4.63930627 | 1.09069348 |
| 1901 | LR-SURV | 1.541913915 | 1.512582559 | 2.956611007 | 1.077338953 | 0.134609042 | 1.73494375 | 1.233721664 | 1.67355368 | 5.703743446 | 1.963252473 |
| 1905 | LR-SURV | 0.802582844 | 1.429007897 | 0.87172467 | 1.993733007 | 0.501682745 | 4.604003368 | 1.581196809 | 13.36988199 | 2.867729785 | 5.984562214 |
| 1906 | LR-SURV | 0.835506074 | 0.608367036 | 5.617563177 | 1.909867063 | 0.183372248 | 0.503788079 | 2.885891278 | 1.070967594 | 1.040956001 | 2.142426505 |
| 1907 | LR-SURV | 0.849521416 | 0.662969263 | 1.332323519 | 1.901940643 | 0.700689519 | 0.138205687 | 0.890703365 | 2.103679358 | 3.789241502 | 2.519689067 |
| 1909 | LR-SURV | 2.040216456 | 0.779712349 | 1.513558507 | 4.536162182 | 0.091559108 | NaN | 0.481970566 | NaN | NaN | NaN |
| 1912 | LR-SURV | 1.781042845 | 3.470188156 | 0.819005159 | 3.64387185 | 0.054215426 | 2.795078669 | 3.03357073 | 5.281379554 | 0.801019401 | 0.895798226 |
| 1914 | LR-SURV | 2.115094646 | 0.127009991 | 1.526200437 | 1.437410592 | 1.696836103 | 0.136871017 | 8.45038428 | 1.213281929 | 0.073705142 | 1.365326235 |
| 1915 | LR-SURV | 1.04154287 | 1.682975167 | 0.85142092 | 2.792335364 | 0.239956903 | 0.767845996 | 0.957282705 | 0.331461871 | 1.106427631 | 3.427711564 |
| 1916 | LR-SURV | 1.254160704 | 1.612181183 | 0.681102789 | 8.279090116 | 0.087707694 | 1.401419513 | 2.420016609 | 1.66429921 | 0.501356625 | 0.771236756 |
| 1917 | LR-SURV | 0.696754453 | 0.503831731 | 0.845539727 | 5.507784917 | 0.611679242 | 2.054644631 | 0.440439861 | 0.720422341 | 1.470094698 | 0.478717255 |
| 1918 | LR-SURV | 1.401257624 | 1.752013497 | 0.776977348 | 5.694117336 | 0.09206823 | 1.773855989 | 1.808780005 | 0.107686912 | 0.596216865 | 1.542473511 |
| 1919 | LR-SURV | 1.393508905 | 0.371391806 | 0.440723107 | 4.442809315 | 0.986812039 | 0.046035445 | 1.382248445 | 0.350361177 | 0.516166741 | 1.67858514 |
| 1920 | LR-SURV | 0.736482026 | 1.149505914 | 0.74636045 | 4.29147092 | 0.368783809 | 1.155797688 | 1.811289245 | 0.459110975 | 0.997168414 | 1.982396732 |
| 1929 | LR-SURV | 1.252423273 | 0.601657305 | 0.379966582 | 5.725779933 | 0.609985656 | 1.209900903 | 1.295058213 | 1.806146386 | 4.96539707 | 0.339914949 |
| 1939 | LR-SURV | 1.675651607 | 0.605842163 | 0.797714521 | 1.019224754 | 1.211544354 | 0.621957924 | 3.144906116 | 2.239093582 | 1.881533649 | 1.016243211 |
| 1940 | LR-SURV | 0.895473238 | 0.54450375 | 0.972617493 | 1.059565094 | 1.990108685 | 4.289736075 | 3.861113461 | 0.013781864 | 0.881424615 | 1.172221915 |
| 1941 | LR-SURV | 1.877381987 | 0.528880573 | 0.654263841 | 0.853505421 | 1.803559754 | 0.244666569 | 1.533700678 | 2.651694966 | 1.760407094 | 0.691233822 |
| 1942 | LR-SURV | 1.171796643 | 2.071985474 | 0.48228052 | 0.902170628 | 0.946613382 | 2.202113591 | 0.258281631 | 0.440408686 | 0.465195514 | 0.710667049 |

SIOPEN results

| PatientID | Type | Reference *AluSq* | Reference *HMBS* | Reference *HPRT1* | Reference *SDHA* | Reference *UBC* | ***CNR1*** | ***GRB10*** | ***KRT19*** | ***PRPH*** | ***QPCT*** |
|---|---|---|---|---|---|---|---|---|---|---|---|
| 1943 | LR-SURV | 1.56560712 | 5.165879133 | 0.936885368 | 0.408801451 | 0.322830149 | 1.689842618 | 0.739702445 | 2.575611196 | 2.936116252 | 0.424914863 |
| 1950 | LR-SURV | 1.1588728 | 2.181036462 | 1.09576957 | 0.30895577 | 1.168653485 | 0.941405958 | 3.075917799 | 0.227023701 | 1.827547737 | 0.607621875 |
| 1959 | LR-SURV | 1.438656553 | 0.605842163 | 0.955246151 | 0.740962391 | 1.620958575 | 0.263319978 | 2.536812602 | 0.099643177 | 0.124300938 | 2.046623483 |
| 1961 | LR-SURV | 2.484098227 | 0.276435325 | 1.684061056 | 0.592740369 | 1.458865846 | 2.501665484 | 0.295456358 | 0.660172444 | 1.0068921 | 1.232207921 |
| 1964 | LR-SURV | 1.19807832 | 0.319306514 | 0.817870563 | 1.540578527 | 2.074620164 | 4.726882126 | 1.02741351 | 0.074786145 | 2.155330471 | 0.677947619 |
| 1965 | LR-SURV | 1.708490496 | 0.42484659 | 0.674525292 | 1.161083898 | 1.759111856 | 0.775333491 | 1.444947373 | 0.796041101 | 1.635701043 | 1.485805956 |
| 1968 | LR-SURV | 2.1268558 | 0.895654293 | 0.593756968 | 1.816891442 | 0.486613153 | 3.337780051 | 0.880879723 | 6.574653334 | 0.787804242 | 0.449765752 |
| 2025 | LR-SURV | 0.476557507 | 1.295055466 | 0.835055762 | 0.878718043 | 2.208163843 | 3.532988098 | 0.645736261 | 8.333455429 | 5.090858939 | 5.1667093 |
| 2071 | LR-SURV | 1.124062475 | 2.236145229 | 1.599858648 | 0.219375181 | 1.133549366 | 4.404227182 | 1.069559653 | 4.982655489 | 12.95923895 | 2.245825312 |
| 2088 | LR-SURV | 0.53171395 | 0.774326482 | 0.951281644 | 0.871439385 | 2.929891465 | 6.49301656 | 3.067401356 | 3.991455222 | 2.322871087 | 3.475560634 |
| 2099 | LR-SURV | 0.879479724 | 0.585204955 | 0.390648897 | 4.915956747 | 1.011746054 | 1.296739681 | 0.652033028 | 2.208267319 | 5.547774282 | 2.502284303 |
| 2100 | LR-SURV | 1.072310133 | 1.388006081 | 1.569107771 | 0.298018175 | 1.436788016 | 1.329504711 | 1.049001651 | 0.668460782 | 0.778036148 | 1.473498625 |
| 2101 | LR-SURV | 1.266390398 | 0.415526931 | 0.939486571 | 0.515295616 | 3.925420541 | 2.23595149 | 0.882101728 | 0.137634574 | 1.100309273 | 0.394814181 |
| 2105 | LR-SURV | 1.45873945 | 0.434375274 | 4.481384284 | 0.214265611 | 1.643586314 | 3.770847716 | 7.906378098 | 3.537952862 | 16.13262268 | 5.074429781 |
| 2106 | LR-SURV | 1.506000472 | 0.448448397 | 1.999922985 | 1.341151505 | 0.552040961 | 5.066146824 | 0.23309936 | 0.281053138 | 5.532413889 | 0.659409106 |
| 2107 | LR-SURV | 0.622748792 | 0.558261855 | 0.7249455 | 1.546998955 | 2.564801585 | 0.735546722 | 0.439829705 | NaN | 0.837353721 | 0.234107931 |
| 2108 | LR-SURV | 0.880699787 | 0.919558899 | 1.177677439 | 0.554581818 | 1.890604013 | 3.498869579 | 0.635082952 | NaN | 0.068578925 | 2.838730153 |
| 2110 | LR-SURV | 0.2695682 | 1.118072615 | 0.597886874 | 2.221404657 | 2.498127692 | 0.778564727 | 0.821889843 | 0.214182843 | 0.838515345 | 0.822020179 |
| 2119 | LR-SURV | 1.959821449 | 1.685309883 | 0.35599884 | 1.183838452 | 0.718394008 | 0.555126661 | 0.46297845 | 0.316201217 | 0.469081039 | 0.757462155 |
| 2122 | LR-SURV | 0.636716132 | 0.933688934 | 1.171165081 | 0.779960097 | 1.841456378 | 0.56994274 | 0.793893258 | 3.264619443 | 0.223122683 | 0.783087657 |
| 2161 | LR-SURV | 1.744389513 | 0.832206746 | 1.142302258 | 0.917304246 | 0.657401759 | 2.017947847 | 2.299016705 | 4.893663245 | 5.578623126 | 1.779220991 |
| 2163 | LR-SURV | 0.902952638 | 1.031693111 | 0.579124102 | 1.121533118 | 1.652725608 | 5.475113563 | 2.175002334 | 16.80612048 | 0.965875464 | 4.003455345 |
| 2165 | LR-SURV | 1.859251869 | 0.284601247 | 1.128138694 | 1.203696955 | 1.391699007 | 3.985854064 | 2.208423639 | 6.075128087 | 9.012385909 | 2.975734297 |
| 2167 | LR-SURV | 0.614175225 | 3.528399429 | 2.243800555 | 0.368432497 | 0.558197366 | 3.426863884 | 1.010463318 | 3.931050307 | 10.80711166 | 0.976198009 |
| 2169 | LR-SURV | | | no Cq value for at least 1 reference gene | | | NaN | NaN | NaN | NaN | NaN |
| 2170 | LR-SURV | 1.000500732 | 0.470743764 | 0.678276044 | 2.318950817 | 1.349895027 | 1.38596314 | 3.050439144 | 4.080977698 | 2.94426819 | 0.513788219 |
| 2253 | LR-SURV | 1.785987796 | 1.020314482 | 0.572736894 | 0.13768776 | 6.958841859 | 0.810502696 | 1.136830329 | 0.936216974 | 5.594111816 | 1.596868722 |
| 2257 | LR-SURV | 2.473788606 | 0.417258658 | 0.205319769 | 0.486149594 | 9.705800608 | 3.067129091 | 4.941732605 | 0.274506997 | 2.538381815 | 0.494912613 |
| 2753 | LR-SURV | 4.391525626 | 0.347964948 | 0.379966582 | 0.422631505 | 4.075133217 | 0.449031602 | 5.629539638 | 1.044574092 | 5.03471143 | 9.146664375 |
| 2756 | LR-SURV | 1.541913915 | 1.122732231 | 0.803263064 | 0.327024861 | 2.198999418 | 0.418961299 | 0.56762772 | 1.990201943 | 0.877766485 | 0.948188443 |
| 2794 | LR-SURV | 3.34666054 | 0.42191196 | 0.960557874 | 0.113084431 | 6.519888183 | 0.567577337 | 0.356263589 | 4.996489512 | 3.836814699 | 1.465350426 |
| 2795 | LR-SURV | 1.932840008 | 0.909417007 | 0.299358148 | 0.451711085 | 4.207161565 | 1.592053579 | 0.731544205 | 0.932331442 | 2.709279229 | 0.558351569 |
| 192 | LR-SURV | 1.100929604 | 1.257897082 | 1.308527681 | 0.883604225 | 0.624531923 | 0.386593394 | 0.383425169 | 0.605800564 | 0.159973971 | 0.201275812 |
| 332 | LR-SURV | 0.3985195 | 1.144735181 | 0.896232187 | 1.74771776 | 1.399437661 | 1.436829513 | 1.079989248 | 2.791256734 | 0.993029919 | 0.812954049 |
| 1535 | LR-SURV | 1.0386591 | 1.122732231 | 0.88511968 | 1.069897229 | 0.905538084 | 13.87955 | 0.878440788 | 3.4174372 | 1.182556481 | 3.740537811 |
| 1672 | LR-SURV | 0.489955362 | 1.605490233 | 1.918454361 | 1.529936974 | 0.433122559 | 2.480943515 | 2.393326044 | 3.13597667 | 0.482268702 | 0.894557246 |
| 1695 | LR-SURV | 1.838746024 | 0.510864959 | 1.085187582 | 0.95361063 | 1.028717764 | 2.673795057 | 1.254416942 | 0.446556562 | 1.511424685 | 0.977552245 |
| 1696 | LR-SURV | 1.30560838 | 1.162325297 | 1.807435851 | 0.783210614 | 0.465498112 | 0.458466711 | 1.540092442 | 0.014812047 | 1.142274266 | 6.204202237 |
| 1697 | LR-SURV | 1.841296835 | 0.675025491 | 0.870517041 | 2.417426828 | 0.382318581 | 1.537822389 | 1.049001651 | 2.311636935 | 3.382076027 | 0.900779384 |
| 1753 | LR-SURV | 0.589156256 | 1.02741133 | 2.197623807 | 0.269708374 | 2.787259298 | 0.622820739 | 0.471397806 | NaN | 0.177256335 | 2.761104928 |
| 1754 | LR-SURV | 0.342629725 | 0.676899656 | 0.754683922 | 1.955411472 | 2.921779332 | 13.87955 | 2.089296071 | 0.344580944 | 8.350769193 | 3.819134462 |
| 1755 | LR-SURV | 0.942603029 | 5.684422897 | 1.871174939 | 0.462482994 | 0.215662497 | 0.276411374 | 0.854419779 | 1.594290827 | 1.50723993 | 0.797328263 |
| 1756 | LR-SURV | 0.289316844 | 2.484600483 | 0.80103903 | 2.894817147 | 0.599922158 | 0.100195449 | 3.602546243 | 0.338896073 | 0.095253049 | 1.071213253 |
| 1757 | LR-SURV | 0.390859558 | 2.633559197 | 0.639025073 | 2.902854421 | 0.523712601 | 0.140134967 | 0.433173517 | 0.786170877 | 1.043846149 | 0.404229275 |
| 1762 | LR-SURV | 1.216488112 | 1.105741298 | 0.973966762 | 0.745082572 | 1.024448331 | 3.233034469 | 13.65177795 | 0.779658812 | 5.097921262 | 1.057931131 |
| 1763 | LR-SURV | 0.483210002 | 0.797200151 | 0.823559303 | 2.502677201 | 1.259496595 | 3.228555637 | 1.337016203 | 0.00415457 | 0.166998736 | 0.894557246 |
| 1764 | LR-SURV | 1.333041987 | 1.280772191 | 1.023807475 | 0.857062442 | 0.667503461 | 15.94341625 | 2.681456685 | 1.303973749 | 5.396070102 | 1.709110546 |
| 1769 | LR-SURV | 0.505128456 | 2.009746989 | 0.975317903 | 0.816470249 | 1.237001476 | 5.045121064 | 2.519289557 | 3.188581654 | 2.087692298 | 5.992864332 |
| 1770 | LR-SURV | 0.985359597 | 0.586829739 | 1.12345664 | 1.527817501 | 1.007547058 | 24.06540652 | 1.968391169 | 1.076922795 | 3.151216969 | 1.033290359 |
| 1779 | LR-SURV | 0.239271791 | 0.428989395 | 0.976670918 | 8.165109436 | 1.221663724 | 2.276612715 | 1.898706548 | 1.607607069 | 0.487647002 | 1.687919048 |
| 1787 | LR-SURV | 1.499750198 | 1.325938525 | 1.209109114 | 0.269334738 | 1.544186732 | 0.20234497 | 1.990342827 | 3.233092595 | 1.560392632 | 1.508637 |
| 1800 | LR-SURV | 0.884370142 | 1.419137018 | 0.616402426 | 0.430914103 | 2.999760295 | 0.947953954 | 1.730293709 | 11.47891857 | 1.214118376 | 0.655762685 |
| 1855 | LR-SURV | 2.389522285 | 0.517278771 | 2.182443711 | 0.175491398 | 2.112347547 | 1.335045479 | 1.28789676 | 3.282772622 | 1.269190907 | 4.675905309 |
| 1885 | LR-SURV | 1.318339756 | 1.495900159 | 1.092735659 | 0.660427556 | 0.702634939 | 3.114258906 | 1.689992623 | 6.574653334 | 3.195206319 | 0.725599675 |
| 1894 | LR-SURV | 0.977197598 | 1.247477581 | 1.076198682 | 0.888517577 | 0.857879678 | 0.170387203 | 1.834029633 | NaN | 0.57590753 | 1.938909833 |
| 1895 | LR-SURV | 0.641144843 | 1.485567222 | 0.345784341 | 5.623515186 | 0.5399311 | 8.508406072 | 0.662970669 | 1.421008148 | 1.608715368 | 0.610154165 |
| 1897 | LR-SURV | 0.312672062 | 2.211482597 | 0.908743122 | 0.478129134 | 3.328442446 | 0.511529337 | 2.145058434 | 0.030414747 | 0.334460813 | 2.623050738 |
| 1902 | LR-SURV | 0.929625934 | 2.229953905 | 0.497905703 | 4.618653198 | 0.209765194 | 5.55924471 | 0.323764553 | 2.223627033 | 2.642510293 | 1.551050559 |
| 1910 | LR-SURV | 0.53171395 | 1.110349523 | 0.839699157 | 3.36701984 | 0.599091065 | 0.558213488 | 2.423373791 | NaN | 2.613365826 | 0.499046348 |
| 1944 | LR-SURV | 3.498465397 | 0.741754608 | 0.635491373 | 2.21832727 | 0.273354915 | 3.963812963 | 4.01393415 | 1.722990595 | 1.640242462 | 1.45926886 |
| 1945 | LR-SURV | 1.262884082 | 0.628077237 | 0.399410577 | 2.027177226 | 1.557084482 | 1.643633783 | 0.337981679 | 0.19011112 | 0.245519427 | 1.489931201 |
| 1946 | LR-SURV | 3.355952328 | 0.888235347 | 0.681102789 | 0.752348094 | 0.65467338 | 0.534732359 | 2.799204617 | 0.729467105 | 1.832621806 | 0.760618911 |
| 1947 | LR-SURV | 2.10923848 | 0.331944485 | 1.011113144 | 1.568594256 | 0.900530611 | 2.962651415 | 1.747166318 | 1.305782694 | 0.382600083 | 0.439899454 |
| 1948 | LR-SURV | 2.327404905 | 0.688254427 | 0.839699157 | 0.859442021 | 0.86504508 | 0.510113041 | 2.440229689 | 0.290561372 | 0.856134335 | 0.527500993 |
| 1951 | LR-SURV | 1.790946477 | 0.245367128 | 1.25696497 | 0.860634287 | 2.103580783 | 2.298812643 | 2.248584264 | 3.224140974 | 2.278223191 | 0.965431439 |
| 1952 | LR-SURV | 1.331195279 | 1.060697953 | 0.830438045 | 0.87385888 | 0.975928407 | 0.409770744 | 1.415210786 | 0.566801243 | 1.132812501 | 0.801761871 |
| 1953 | LR-SURV | 1.240328461 | 0.948036092 | 0.913796261 | 0.315010587 | 2.954363251 | 1.694534358 | 2.012539291 | 0.88203929 | 2.728123756 | 0.46305187 |
| 1954 | LR-SURV | 1.292999953 | 1.26840361 | 0.829287611 | 0.6256693 | 1.175151876 | 0.946640722 | 0.659304553 | 0.035131642 | 1.513521418 | 1.728170685 |
| 1958 | LR-SURV | 3.170526032 | 0.593374111 | 1.120346071 | 0.700993136 | 0.676821462 | 0.501697234 | 23.80209983 | 0.384995857 | 0.172170406 | 0.656672395 |
| 1962 | LR-SURV | 2.561026573 | 2.127285825 | 1.797441036 | 0.623936979 | 0.163668345 | 1.798618096 | 1.105743644 | 0.530312561 | 3.326278787 | 1.07567758 |
| 1963 | LR-SURV | 1.291208716 | 0.225471015 | 1.691079453 | 1.149871162 | 1.766443031 | 1.422953866 | 0.944103511 | NaN | 1.511424685 | 0.636064826 |
| 1966 | LR-SURV | 1.414921704 | 0.638613028 | 0.441334503 | 2.854963344 | 0.87833745 | 3.273624594 | 2.393326044 | 0.925891358 | 0.408358763 | 0.535606626 |
| 1967 | LR-SURV | 1.426739774 | 1.27016321 | 1.118794017 | 0.704891064 | 0.69971883 | 1.368778391 | 1.395727161 | 1.110273284 | 2.411463696 | 3.633212413 |
| 2084 | LR-SURV | 0.794832199 | 0.566840163 | 4.078233684 | 0.620486713 | 0.877120659 | 1.020226082 | 0.465552879 | 0.239303736 | 4.979183176 | 0.542331064 |
| 2102 | LR-SURV | 0.446496949 | 0.978751073 | 2.686903382 | 0.305548271 | 2.787259298 | 1.138306357 | 2.57579352 | NaN | 1.089683445 | 29.34912759 |
| 2103 | LR-SURV | 0.431287624 | 1.514680898 | 1.068764834 | 1.319025287 | 1.08586686 | 0.462296033 | 1.783877996 | 5.71563911 | 0.612978803 | 0.635183664 |
| 2104 | LR-SURV | 1.042987756 | 1.457013141 | 1.006916775 | 0.474168662 | 1.378259191 | 5.90071136 | 1.071043406 | 3.479583978 | 6.858576097 | 2.461001802 |
| 2118 | LR-SURV | 0.854245248 | 14.8770223 | 1.088200536 | 0.413360438 | 0.174929708 | 0.748922786 | 2.869932786 | 4.002537242 | 2.731908361 | 2.299381285 |
| 2160 | LR-SURV | 1.075287334 | 0.581162649 | 0.571943462 | 1.187125303 | 2.35682922 | 4.839603614 | 1.233721664 | 1.294966545 | 0.733012188 | 1.498216083 |
| 2660 | LR-SURV | 1.552638775 | 14.69256004 | 0.390648897 | 0.154692185 | 0.725399282 | 0.095053851 | 1.340728343 | 0.651986875 | 2.660890405 | 0.043683907 |
| 2738 | LR-SURV | 0.848344545 | 0.873581306 | 1.476257557 | 0.313268632 | 2.917731692 | 1.397539334 | 0.551341059 | 2.446831623 | 0.882647376 | 9.654795585 |
| 2743 | LR-SURV | 0.830885867 | 0.488020579 | 1.817486243 | 0.136358092 | 9.951039381 | 0.096247234 | 0.168522946 | 3.412702908 | 5.855973767 | 2.641295499 |
| 2760 | LR-SURV | 2.873327067 | 0.59255209 | 0.64704791 | 0.501900129 | 1.808567222 | 0.616806079 | 3.319617342 | 0.273746956 | 0.763081519 | 0.457310425 |
| 2765 | LR-SURV | 3.865680015 | 0.634201805 | 0.632853926 | 0.395972521 | 1.62771399 | 2.314802149 | 0.283028051 | 1.196578318 | 2.964747195 | 0.62817817 |
| 2796 | LR-SURV | 2.080199971 | 0.200130629 | 0.719937935 | 0.721700531 | 4.62305723 | 1.927711144 | 7.037275283 | 1.038797773 | 4.903830424 | 2.035306 |
| 193 | LR-SURV | 0.786065928 | 2.211482597 | 1.358433973 | 1.352353412 | 0.313132948 | 0.321499609 | 0.255433022 | 0.920771335 | 2.694297372 | 1.357776211 |
| 267 | LR-SURV | 1.359166522 | 0.605002869 | 1.817486243 | 0.714730977 | 0.936173104 | 4.008017726 | 0.595022839 | 1.465014381 | 1.463993442 | 1.240778604 |
| 1178 | LR-SURV | 0.803696231 | 1.372697646 | 1.369780231 | 1.566421229 | 0.422448454 | 6.041425036 | 0.934986182 | 1.55069486 | 1.294064109 | 2.350953028 |
| 1507 | LR-SURV | 0.475897316 | 3.033564295 | 1.12345664 | 0.790847934 | 0.779622278 | 0.346010779 | 1.08449015 | 9.043710601 | 0.452474719 | 0.239358628 |
| 1513 | LR-SURV | 0.780636174 | 1.121176872 | 1.684061056 | 1.479871821 | 0.45845347 | 4.604003368 | 5.355483945 | 4.086639058 | 2.989510112 | 1.329833581 |
| 1722 | LR-SURV | 1.108587175 | 1.357558049 | 0.925268797 | 1.386523648 | 0.517936531 | 0.271474555 | 0.676901092 | 0.662923741 | 0.443162924 | 0.197133616 |
| 1761 | LR-SURV | 0.324597146 | 1.093545985 | 2.182443711 | 2.444386168 | 0.528086887 | 0.249461466 | 1.723112563 | 1.152618297 | 0.612978803 | 0.143114893 |
| 1774 | LR-SURV | 0.856617005 | 0.784048002 | 1.381221259 | 0.747151246 | 1.442775892 | 4.307613735 | 0.635082952 | 1.859500101 | 0.564055525 | 3.149768317 |
| 1777 | LR-SURV | 0.874616349 | 3.834434668 | 1.194117203 | 0.164878161 | 1.514505925 | 1.069464718 | 2.76066714 | 3.82355553 | 2.005426499 | 2.858475083 |
| 1819 | LR-SURV | 1.206411614 | 3.92587405 | 0.790010889 | 0.131530768 | 2.031925099 | 0.389822397 | 0.033702909 | 0.085313321 | 0.593742418 | 2.142426505 |
| 1832 | LR-SURV | 0.510054114 | 2.772166459 | 0.756779254 | 0.490210137 | 1.906395197 | 0.036622876 | 0.274907267 | 21.80995407 | 3.721564115 | 0.320244504 |
| 1839 | LR-SURV | 1.070824626 | 1.257897082 | 0.645256397 | 0.429721011 | 2.677426966 | 31.75449428 | 0.388777585 | 0.635918959 | 4.613651689 | 2.293014876 |
| 1846 | LR-SURV | 2.220250137 | 0.314910527 | 0.570359894 | 0.522488874 | 4.799377261 | 0.079709135 | 0.322868131 | 0.757288448 | 0.309478071 | 0.456676898 |
| 1850 | LR-SURV | 0.569876901 | 3.033564295 | 0.971270093 | 2.641710224 | 0.225444969 | 0.084137274 | 0.405287321 | 0.672177812 | 1.772651694 | 10.98334593 |
| 1861 | LR-SURV | 0.413718743 | 3.504026994 | 1.177677439 | 0.355882333 | 1.645866388 | 5.266662054 | 0.666657171 | 7.971851448 | 3.467531746 | 1.031858907 |
| 1880 | LR-SURV | 2.150574668 | 1.7254993 | 0.694450825 | 1.123088971 | 0.34552146 | 0.613395245 | 0.73357529 | 2.50171046 | 2.534865309 | 1.936223796 |
| 1881 | LR-SURV | 0.357179757 | 3.964156416 | 1.84541392 | 0.869026588 | 0.440388049 | 0.029054167 | 0.067126068 | 3.166556512 | 4.146543108 | 0.390459742 |
| 1888 | LR-SURV | 0.723328159 | 1.144735181 | 0.890041459 | 0.653143645 | 2.077498193 | 5.5515433 | 1.754447709 | 0.611707906 | 6.319932242 | 1.694953523 |
| 1889 | LR-SURV | 0.768822372 | 5.10890422 | 0.742233203 | 0.504690985 | 0.679642145 | 4.826203968 | 1.355680254 | 2.987450537 | 3.429288049 | 9.981424428 |
| 1890 | LR-SURV | 0.841317479 | 2.626267534 | 1.197432593 | 0.961575619 | 0.393067019 | 1.366882176 | 1.943984814 | 1.124212551 | 0.061550274 | 0.578041795 |
| 1956 | LR-SURV | 2.214102823 | 3.114528748 | 0.495152368 | 1.222193084 | 0.239624483 | 0.756225755 | 1.759318821 | 8.723546827 | 3.12511459 | 0.776601131 |
| 1960 | LR-SURV | 1.311049559 | 1.359441329 | 0.747395843 | 1.251338601 | 0.599922158 | 0.332376178 | 0.687302422 | 2.232894095 | 1.983308483 | 0.34609637 |
| 2114 | LR-SURV | 0.573045748 | 1.601045035 | 0.385805187 | 1.180560701 | 2.393044496 | 0.345531439 | 0.385557236 | 4.773061173 | 0.315105991 | 0.10549494 |
| 2661 | LR-SURV | | | no Cq value for at least 1 reference gene | | | NaN | NaN | NaN | NaN | NaN |
| 318 | LR-SURV | 0.568299055 | 1.382245513 | 0.797714521 | 3.674307125 | 0.434325096 | NaN | 1.089009809 | NaN | 0.421004947 | 0.487422824 |
| 1179 | LR-SURV | 0.787156402 | 1.040310282 | 1.15985499 | 1.154663299 | 0.911836599 | 0.208033661 | 0.587645068 | 1.40728529 | 0.426881957 | 0.477391808 |
| 1773 | LR-SURV | 1.201404708 | 1.363215731 | 1.478305503 | 0.405415239 | 1.018783304 | 0.505186814 | 1.167171814 | 1.882845767 | 0.260961537 | 0.427277637 |
| 1792 | LR-SURV | 1.820989019 | 0.705644715 | 0.670795281 | 1.308099445 | 0.886902379 | 7.916643517 | 1.037432125 | NaN | 0.796589899 | 14.1550576 |
| 1797 | LR-SURV | 0.975843853 | 1.7254993 | 0.734047051 | 0.777800584 | 1.040190107 | 2.629682984 | 4.701160544 | 0.967889883 | 1.349017526 | 2.975734297 |
| 2067 | LR-SURV | 0.313540176 | 1.256154474 | 1.288725003 | 0.447348853 | 4.404099985 | 0.488655247 | 0.885777925 | 8.485000981 | 0.759914542 | 0.843959524 |
| 2068 | LR-SURV | 0.731394773 | 0.500351511 | 2.72819061 | 0.824431893 | 1.214908129 | 0.425989238 | 5.452874491 | 13.24076733 | 6.661786372 | 2.67075144 |
| 2085 | LR-SURV | 0.445878402 | 0.446587225 | 0.656990514 | 4.881999753 | 1.565742776 | 0.135361455 | 0.866347034 | 3.537952862 | 2.17634774 | 0.797328263 |
| 2121 | LR-SURV | 0.598209291 | 1.028836613 | 0.731000574 | 0.722701715 | 3.075555952 | 1.589848053 | 1.092033375 | 6.484138139 | 1.194087941 | 0.24269995 |
| 1780 | | 1.381966096 | 0.194658047 | 3.377473487 | 0.299260178 | 3.677810693 | 6.394754123 | 1.759318821 | 0.744794759 | 3.257824959 | 8.725547837 |
| 1785 | | 0.414867406 | 1.26840361 | 1.064329196 | 2.302932654 | 0.775311099 | 3.632313913 | 0.405849558 | 0.336555147 | 1.599819443 | 3.83505087 |
| 1791 | | 0.910494509 | 1.901336507 | 0.617257534 | 0.710778635 | 1.316627487 | 3.170892326 | 4.178592142 | 0.000721314 | 0.463907505 | 3.714700029 |
| 1794 | | 1.347908179 | 2.57221973 | 0.466498531 | 0.634403318 | 0.97457642 | 0.646574667 | 0.383957078 | 2.141935188 | 5.343959562 | 5.386117542 |
| 1796 | | 0.989466125 | 1.632421808 | 0.206461466 | 2.544658794 | 1.17841461 | 3.850081242 | 0.38502311 | 0.928462036 | 1.517723613 | 0.684558488 |
| 1810 | | 0.535412313 | 2.380086306 | 0.788916461 | 1.110702329 | 0.895550828 | 0.93231468 | 0.041723875 | 6.394869093 | 1.420017876 | 0.740846066 |
| 1811 | | 0.864970132 | 0.836834299 | 0.547125781 | 0.60018211 | 4.207161565 | 1.987409118 | 0.531822584 | 2.711168588 | 1.889375022 | 0.70673718 |
| 1812 | | 0.698688947 | 0.729517195 | 1.743449341 | 0.206680219 | 5.444679858 | 2.795078669 | 0.966617453 | 8.345016063 | 6.680282395 | 1.603523742 |
| 1825 | | 0.645604358 | 4.928039736 | 0.516895463 | 0.374093868 | 1.625459063 | 0.778564727 | 0.080048432 | 9.692809025 | 7.239595497 | 0.874933768 |
| 1826 | | 0.777396341 | 2.02653331 | 0.676398068 | 1.148278206 | 0.817248779 | 0.766782273 | 0.484650602 | 2.76047173 | 1.794906995 | 5.40856442 |
| 1835 | | 0.580240226 | 1.544365134 | 0.661560244 | 0.573343729 | 2.942101914 | 7.234477826 | 0.277973055 | 4.900452007 | 8.010593255 | 1.806560681 |
| 1836 | | 1.338597497 | 0.297923512 | 0.466498531 | 4.902345698 | 1.096455471 | 5.318018843 | 0.834519085 | 6.493133296 | 5.648661665 | 0.9070448 |
| 1840 | | 0.759289637 | 1.090518231 | 0.902465974 | 1.597119456 | 0.837898399 | 0.467451596 | 1.133682731 | 4.565949257 | 2.462133674 | 0.454151552 |
| 1854 | | 2.490995167 | 0.806090592 | 0.549405952 | 0.251646811 | 3.602122443 | 5.566956804 | 4.907597564 | 2.575611196 | 4.972285345 | 0.690276232 |
| 1858 | | 0.574636772 | 0.433172598 | 0.543346508 | 5.829904385 | 1.268257087 | 0.901805922 | 4.753588266 | 3.703565433 | 0.87533617 | 7.408829916 |
| 1860 | | 0.450226295 | 0.562924712 | 0.428671511 | 5.54609459 | 1.659613413 | 2.564875661 | 1.401543915 | 0.129131675 | 0.276606908 | 1.228796246 |
| 1884 | | 0.33791264 | 1.033124334 | 1.074707787 | 3.220918647 | 0.82750918 | 0.094397266 | 0.448449348 | 0.16413045 | 1.560392632 | 3.167282857 |
| 1934 | | 1.843851185 | 1.607717459 | 0.859723379 | 0.352445532 | 1.113303716 | 2.995691143 | 0.796097451 | 2.347157782 | 0.334460813 | 2.029670749 |

SIOPEN results

| PatientID | Type | Reference *AluSq* | Reference *HMBS* | Reference *HPRT1* | Reference *SDHA* | Reference *UBC* | *CNR1* | *GRB10* | *KRT19* | *PRPH* | *QPCT* |
|---|---|---|---|---|---|---|---|---|---|---|---|
| 2089 | | 1.125621837 | 2.16297035 | 1.125015162 | 0.448590888 | 0.813856995 | 5.429761723 | 4.623601145 | 3.627347159 | 4.370835527 | 0.965431439 |
| 1702 | | 1.620818136 | 0.191978132 | 1.665487406 | 1.716502814 | 1.124159872 | 0.957197735 | 2.436849156 | 1.687531961 | 2.352034286 | 0.680772995 |
| 1793 | | 1.204740331 | 3.58261717 | 1.204091013 | 0.813081696 | 0.236653345 | 4.528046861 | 0.844996312 | 2.881689364 | 0.618098681 | 1.862506828 |
| 1795 | | 1.434673275 | 1.882975056 | 0.888808454 | 0.59603635 | 0.698749486 | 3.708636549 | 1.493831007 | 7.829470955 | 2.310026002 | 1.944293092 |
| 1813 | | 0.728359305 | 1.121176872 | 0.493781416 | 0.78756572 | 3.148898428 | 1.259532972 | 3.708965759 | 3.839490363 | 0.420421715 | 1.339083305 |
| 1848 | | 0.53171395 | 1.293261382 | 1.204091013 | 0.980420274 | 1.231867615 | 1.957332548 | 0.635963975 | 5.052210767 | 3.85280479 | 0.6494299 |
| 1935 | | 1.189802589 | 0.813951021 | 3.530676023 | 0.279994855 | 1.044525144 | 5.429761723 | 1.221807456 | 4.46578819 | 0.978001844 | 1.590241323 |
| 1937 | | 1.385803036 | 0.621150121 | 1.423995359 | 7.227377356 | 0.11287886 | 1.231903193 | 0.275288634 | 3.996992391 | 2.883676027 | 0.972146546 |
| 1957 | | 1.0386591 | 1.325938525 | 0.377341961 | 2.440999877 | 0.788316689 | 0.661993469 | 0.759444767 | 1.073941067 | 0.017602316 | 0.490813118 |
| 520 | | 0.469345497 | 2.533292895 | 2.617055363 | 1.043530105 | 0.307966904 | 0.132025535 | 0.573162826 | 0.00940025 | 0.631086248 | 3.883199194 |
| 1516 | | 0.219261166 | 2.16297035 | 2.479319924 | 1.477821704 | 0.5754838 | 0.578700537 | 3.179978363 | 2.582762216 | 4.620052004 | 7.777172369 |
| 1760 | | 0.509347519 | 1.463085306 | 1.011113144 | 0.316323407 | 4.195512992 | 0.1957235 | 0.666657171 | 2.289313161 | 3.372711908 | 1.45926886 |
| 1772 | | 2.350100123 | 1.563754341 | 4.089556638 | 2.122069964 | 0.031355172 | 0.483936241 | 0.351358803 | NaN | 0.56640625 | 1.223696439 |
| 1775 | | 0.999114704 | 0.712525667 | 0.998576212 | 0.651335255 | 2.159724398 | NaN | 1.823887758 | NaN | 1.860782058 | 1.344663993 |
| 1781 | | 1.585263016 | 1.146323221 | 1.709938508 | 0.557665614 | 0.577081594 | 13.78367695 | 3.016795595 | 7.125122541 | 1.961434407 | 3.233834203 |
| 1802 | | 1.103986264 | 1.50005343 | 0.865703228 | 0.304702285 | 2.289205887 | 9.018508698 | 1.405435212 | 5.281379554 | 1.443838212 | 3.29720986 |
| 1805 | | 1.495597767 | 0.665732216 | 1.013920435 | 0.467640642 | 2.118212344 | 0.603275496 | 5.165890091 | 2.925969694 | 0.383662347 | 0.870095531 |
| 1816 | | 1.186508324 | 7.255192477 | 0.568780711 | 0.384078263 | 0.531760021 | 1.849184133 | 0.452195001 | 12.42276857 | 0.567978841 | 3.329361935 |
| 1818 | | 0.416019259 | 1.678315431 | 0.424531775 | 1.146687457 | 2.942101914 | 0.635026582 | 0.356757818 | 0.374468125 | 0.141798 | 0.616958654 |
| 1859 | | 1.008857369 | 4.422965193 | 2.00269739 | 0.245785587 | 0.455286701 | 0.180852932 | 0.65565871 | 2.771976115 | 0.00293975 | 2.551323278 |
| 1887 | | 1.50808968 | 1.210005738 | 1.029500438 | 0.560766559 | 0.949241593 | 0.981383478 | 0.669435493 | NaN | 2.32932039 | 1.506547034 |
| 1949 | | 2.603986957 | 0.878438925 | 1.013920435 | 1.977218379 | 0.218067582 | 0.444695247 | 1.020316646 | 1.623283324 | 0.998551742 | 0.157044543 |
| 1955 | | 2.522269131 | 0.220219464 | 1.286939689 | 1.401986271 | 0.997817047 | 1.209900903 | 1.756881577 | 15.59395343 | 0.660627573 | 2.334713809 |
| 2069 | | 0.65643416 | 0.43377352 | 0.470394939 | 1.979961287 | 3.770738811 | 0.086143687 | 2.413316192 | 0.071839187 | 0.506947794 | 0.220866377 |
| 2070 | | 1.450672867 | 0.965276318 | 1.564763306 | 0.182943774 | 2.494666951 | 0.905564237 | 2.666628682 | 4.361773414 | 0.070997355 | 24.50910189 |
| 2098 | | 0.498174203 | 1.737501097 | 0.604554557 | 0.991354 | 1.927655471 | 0.29831096 | 0.842656729 | 0.473985499 | 1.080657304 | 3.278976846 |
| 2666 | | 0.321908421 | 1.107275244 | 1.73380836 | 0.221821671 | 7.294692794 | 0.929733335 | 0.100343352 | 2.302043071 | 4.863210676 | 0.77552528 |
| 2704 | | 1.057547323 | 2.046294639 | 1.404390794 | 0.708810668 | 0.464209265 | 0.566791053 | 0.598331511 | 0.064208799 | 2.475824567 | 0.677947619 |
| 2705 | | 1.059014411 | 1.27016321 | 1.386977558 | 0.372541286 | 1.438781209 | 0.922032104 | 1.118074986 | 0.959872591 | 2.694297372 | 2.029670749 |
| 2706 | | 0.713369887 | 0.750026715 | 0.896232187 | 1.419587939 | 1.46901306 | 0.077637038 | 1.465118087 | 0.722422547 | 0.086563887 | 0.555263979 |
| 2742 | | 0.750915368 | 0.888235347 | 2.036291984 | 0.795245508 | 0.925847972 | NaN | 0.306724848 | 2.180886707 | 2.64617613 | 0.215423227 |
| 2745 | | 3.754763974 | 0.002183736 | 8.156467367 | 1.001020988 | 14.93729721 | NaN | 5.377803156 | 50.10611672 | 77.48843441 | 8.557834116 |
| 2746 | | | | no Cq value for at least 1 reference gene | | | NaN | NaN | NaN | NaN | NaN |
| 2748 | | 0.974491983 | 1.210005738 | 0.454371561 | 0.199639932 | 9.349227876 | 0.183377544 | 0.539994624 | 0.470059349 | 1.013895589 | 2.205713963 |
| 2754 | | 1.841296835 | 1.508394598 | 2.070450117 | 0.744050384 | 0.233719048 | 0.255410349 | 0.301246618 | 0.000982616 | 2.374969673 | 9.443002906 |
| 2755 | | 1.057547323 | 0.421327471 | 0.607074064 | 2.835242671 | 1.303912647 | 1.007576158 | 1.463088409 | 3.577408399 | 2.374969673 | 0.762730721 |
| 2759 | | 1.520685931 | 3.427160812 | 0.427484627 | 0.322522613 | 1.391699007 | 0.396361541 | 0.317101575 | NaN | 0.168627191 | 0.112130116 |
| 2761 | | 1.94358772 | 0.13039983 | 1.026650011 | 8.348240843 | 0.460364095 | 0.44716801 | 1.430993332 | 3.131632295 | 3.520814189 | 3.242812736 |
| 2764 | | | | no Cq value for at least 1 reference gene | | | NaN | NaN | NaN | NaN | NaN |
| 2793 | | 20.01101197 | 4.06995795 | 1.073218958 | 0.003162919 | 3.617134445 | 8.711305184 | 0.595022839 | 28.85830833 | 6.96396567 | 2.992281107 |
| 1803 | | | | no Cq value for at least 1 reference gene | | | NaN | NaN | NaN | NaN | NaN |
| 2778 | | 2.798632828 | 0.177638216 | 2.090639692 | 0.893458251 | 1.076872332 | 0.439790666 | 1.127413657 | 19.33194415 | 5.987314654 | 0.206361496 |
| 2782 | | 0.761397756 | 0.840321842 | 0.719937935 | 0.459925541 | 4.72019749 | 0.569153179 | 2.722660219 | 0.815024214 | 8.190259091 | 5.116813727 |
| 764 | | 1.353525645 | 0.716487723 | 2.152397363 | 0.87023215 | 0.550512498 | 0.478598868 | 4.515910673 | 8.784223894 | 0.9889086 | 2.57620199 |
| 323 | | 1.381966096 | 0.696895328 | 0.644362502 | 1.26529363 | 1.273542603 | 1.4209826 | 1.59661553 | 5.598012958 | 2.556037657 | 1.71148152 |
| 330 | | 1.546194941 | 1.133680327 | 1.545361589 | 1.134040546 | 0.325526573 | 2.360166908 | 1.481457202 | 3.523269484 | 0.886325847 | 2.357480298 |
| 1495 | | 2.29218299 | 0.706623625 | 1.262203426 | 1.658032427 | 0.295012343 | NaN | 1.098105715 | 1.038797773 | 1.059885994 | 1.872863447 |
| 1508 | | 0.900452589 | 0.986926047 | 1.055513071 | 1.103030147 | 0.966503741 | 2.279770959 | 0.561367306 | 1.862079703 | 0.280468196 | 0.856927891 |
| 1510 | | 0.738526821 | 0.94935126 | 0.738128778 | 2.541133592 | 0.760408847 | 2.467224316 | 1.516785365 | 0.683453375 | 2.099301079 | 1.506547034 |
| 1511 | | 0.867371667 | 0.534778692 | 1.15985499 | 1.86279927 | 0.997817047 | 0.719411381 | 0.454709468 | 1.83135925 | 0.45122193 | 0.227390788 |
| 1512 | | 0.648294942 | 1.054832472 | 1.120346071 | 1.599335069 | 0.816116617 | 1.419014065 | 1.756881577 | 5.707721042 | 4.089456424 | 0.544591252 |
| 1514 | | 0.496106657 | 2.889888617 | 1.679398314 | 0.80188776 | 0.517936531 | 1.101056795 | 0.425436863 | 2.543675836 | 1.429894881 | 0.918432573 |
| 1717 | | 0.641144843 | 0.986926047 | 1.889421322 | 1.626163392 | 0.514358882 | 2.946268452 | 4.880459378 | 10.0346252 | 5.263086445 | 5.304606424 |
| 1765 | | 0.715350513 | 0.434977864 | 0.685840233 | 4.315333989 | 1.08586686 | 0.428357986 | 0.286184402 | 0.085077109 | 0.219746043 | 0.484727458 |
| 1771 | | 0.612474728 | 0.803858729 | 0.813347877 | 2.107411756 | 1.184967279 | 2.133007279 | 1.766650858 | 7.520949587 | 5.314408367 | 43.14871571 |
| 1801 | | 3.662229549 | 0.32421301 | 0.559397009 | 0.446110256 | 3.374905772 | 78.9511781 | 1.516785365 | 0.590870889 | 4.027568402 | 0.683610146 |
| 1806 | | 0.533929891 | 4.914395233 | 2.739560462 | 0.085820863 | 1.620958575 | 0.58840805 | 0.069013239 | 8.128081266 | 5.104993382 | 1.529696788 |
| 1830 | | 1.995461193 | 2.426730483 | 1.980609473 | 0.327478528 | 0.318385651 | 5.45239049 | 4.362082177 | 6.34189867 | 2.047564773 | 2.007285341 |
| 1842 | | 0.934795194 | 2.136151381 | 1.111065943 | 1.185480738 | 0.380204423 | 6.377048656 | 1.742328853 | 17.51980517 | 10.41007885 | 1.614677131 |
| 1844 | | 0.78280356 | 0.668506683 | 0.745326492 | 1.891423232 | 1.355520773 | 1.254305599 | 7.917346266 | 6.988170589 | 0.480933421 | 0.83812987 |
| 1921 | | 1.895688898 | 1.296852039 | 1.707569672 | 2.35458405 | 0.101169563 | 1.368778391 | 2.015331197 | 1.671235249 | 0.852581166 | 0.279563073 |
| 1922 | | 0.609087846 | 0.686348823 | 0.935587469 | 16.53524162 | 0.154625185 | 0.512238959 | 0.872372955 | 0.359213836 | 0.065240384 | 0.36684581 |
| 1928 | | 1.21480287 | 0.272251875 | 0.812221119 | 0.398726728 | 9.336276074 | 2.889638238 | 1.02741351 | NaN | 1.787457687 | 9.64142047 |
| 2021 | | 0.402405582 | 0.711538581 | 0.804377395 | 1.049332738 | 4.137752125 | 1.725349804 | 1.489694964 | 0.001284048 | 2.287717785 | 1.847079182 |
| 2022 | | 0.447736619 | 1.359441329 | 0.590473592 | 1.00938197 | 2.756518382 | 1.376389593 | 1.601048431 | 4.052788326 | 2.067531186 | 1.944293092 |
| 2086 | | 0.394670946 | 1.592191526 | 0.531427372 | 0.818737126 | 3.65747302 | 2.54715877 | 0.751068787 | 7.710983209 | 1.511424685 | 2.509231735 |
| 2087 | | 0.314846868 | 1.39959934 | 0.738128778 | 1.925819141 | 1.596427706 | 3.765623832 | 1.31131742 | 2.771976115 | 3.204077604 | 0.946874886 |
| 2112 | | 1.075287334 | 0.816210904 | 0.424531775 | 5.607945086 | 0.478585045 | 0.498231758 | 0.295866231 | 0.878378609 | 2.952442762 | 0.544591252 |
| 2115 | | 1.544052944 | 0.794992904 | 0.880225117 | 1.116878478 | 0.828657147 | 0.686289358 | 0.290179388 | 3.193005032 | 3.515936686 | 1.653182176 |
| 2116 | | 0.857805352 | 2.363645849 | 0.228132587 | 0.624802539 | 3.460180319 | 1.645913923 | 0.793893258 | 0.073654364 | 0.613829161 | 0.313653967 |
| 2159 | | 0.343581014 | 3.206532187 | 0.805493272 | 0.813081696 | 1.385923112 | 0.668448764 | 0.691124223 | 1.811161036 | 0.053880576 | 1.09220655 |
| 2166 | | 1.591869668 | 0.675025491 | 1.958765165 | 0.99548551 | 0.477259964 | 10.48960821 | 2.987662014 | 4.853127715 | 1.850492247 | 1.930862879 |
| 2168 | | 1.264636025 | 0.877221994 | 1.691079453 | 0.853505421 | 0.624531923 | 0.81162707 | 4.307996116 | 1.421008148 | 2.940189396 | 0.90578824 |
| 2713 | | | | no Cq value for at least 1 reference gene | | | NaN | NaN | NaN | NaN | NaN |
| 2715 | | 1.401257624 | 0.279130928 | 2.093539944 | 0.207254053 | 5.892366723 | 3.749995564 | 1.652920055 | 14.46921579 | 10.08339172 | 2.420400377 |
| 2757 | | 1.316513415 | 0.840321842 | 0.380493693 | 0.992729261 | 2.393044496 | 1.491629142 | 1.641502502 | 0.494799116 | 0.030267411 | 0.5088265 |
| 2763 | | 1.970719193 | 0.722472154 | 1.345315402 | 0.660427556 | 0.7905054 | 0.851978476 | 0.868752391 | 3.474763592 | 1.018121042 | 0.33384393 |
| 334 | | 3.631894315 | 0.317100903 | 0.532164598 | 2.796209048 | 0.583517255 | 6.151289557 | 1.041755668 | 5.691917797 | 2.02498201 | 3.408756897 |
| 1751 | | 2.273196221 | 0.136503882 | 0.604554557 | 3.699863952 | 1.440777166 | 0.1025848 | 0.046488476 | 0.000834335 | 0.392811505 | 0.056065058 |
| 1938 | | 1.795918925 | 2.783719583 | 2.486203597 | 0.746116192 | 0.107831248 | 2.505135933 | 0.643056296 | NaN | 2.002648314 | 0.537093704 |
| 1136 | | 0.97179386 | 0.444733778 | 0.254889605 | 7.735380303 | 1.173523898 | 0.021476294 | 0.408106318 | 0.018541668 | 0.018918075 | 0.112441438 |
| 1687 | | 0.355204613 | 5.893047476 | 0.676398068 | 0.370995148 | 1.903754204 | 0.088811413 | 0.354785007 | NaN | 0.240466711 | 0.39372104 |
| 1834 | | 0.842484601 | 0.734591389 | 1.988863748 | 0.178682744 | 4.546786374 | 0.540695738 | 0.368827197 | 0.004337005 | 4.499950373 | 3.320143768 |
| 1786 | | 0.867371667 | 3.776406032 | 0.621550899 | 2.527081551 | 0.194365925 | 1.469055488 | 0.293822534 | 4.266091255 | 0.629338929 | 1.089182505 |
| 2785 | | 1.347908179 | 1.388006081 | 0.222202114 | 3.514872171 | 0.68436943 | 0.055202939 | 1.71596122 | 0.260420482 | 0.262049106 | 0.046046832 |
| 2786 | | 9.786019939 | 0.010919222 | 1.635741126 | 0.243749679 | 23.47164779 | 0.779644795 | 1.71596122 | NaN | 0.414059239 | 0.228338449 |
| 1837 | | 0.761397756 | 1.903974147 | 0.880225117 | 0.466345862 | 1.68044954 | 0.745814571 | 0.159875076 | 3.260096855 | 2.179366891 | 0.7449656 |
| 1933 | | 1.462789547 | 2.991800319 | 0.998576212 | 1.517264094 | 0.150814518 | 1.813640972 | 1.888207021 | 3.828859779 | 1.700441166 | 0.805103248 |
| 1849 | | 0.880699787 | 0.650226333 | 0.850241418 | 0.932691725 | 2.202049992 | 7.144776533 | 0.057712072 | 3.617303946 | 1.950588006 | 7.08734714 |
| 2774 | | 2.515285602 | 1.304063289 | 0.888808454 | 0.84878568 | 0.404117637 | 0.131295456 | 1.256157139 | 1.336921564 | 1.364061878 | 0.066029096 |
| 1856 | | 0.737503715 | 0.494147583 | 1.094251563 | 1.498451327 | 1.673475259 | 3.582306774 | 1.013268804 | 4.753251791 | 5.418558458 | 1.776756176 |
| 1931 | | 3.166133795 | 0.471396806 | 4.204529724 | 0.54922615 | 0.290145251 | NaN | 4.20182754 | NaN | 10.92763345 | 0.207508986 |
| 1932 | | 0.636716132 | 0.779712349 | 1.317629211 | 2.91091401 | 0.525166655 | 1.813640972 | 0.481970566 | 2.389841315 | 2.820418191 | 3.220412992 |
| 2783 | | 1.56560712 | 0.363244759 | 0.367532681 | 1.042084468 | 4.591123441 | 0.137441433 | 4.242800811 | 5.045211768 | 0.016908695 | 0.976198009 |

SIOPEN results

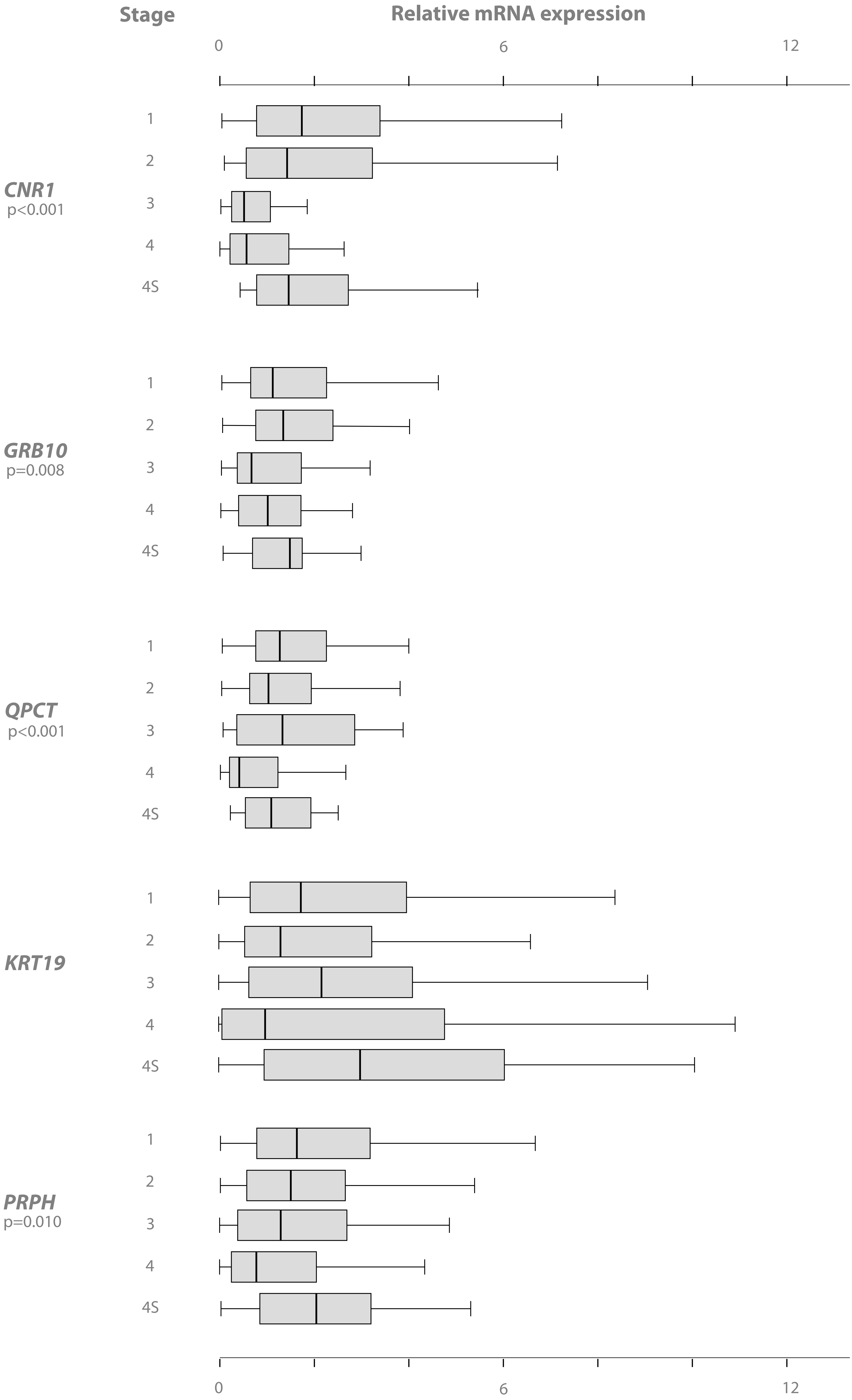

Stage
Relative mRNA expression
0
6
12
CNR1
p<0.001
GRB10
p=0.008
QPCT
p<0.001
KRT19
PRPH
p=0.010
1
2
3
4
4S


Expression

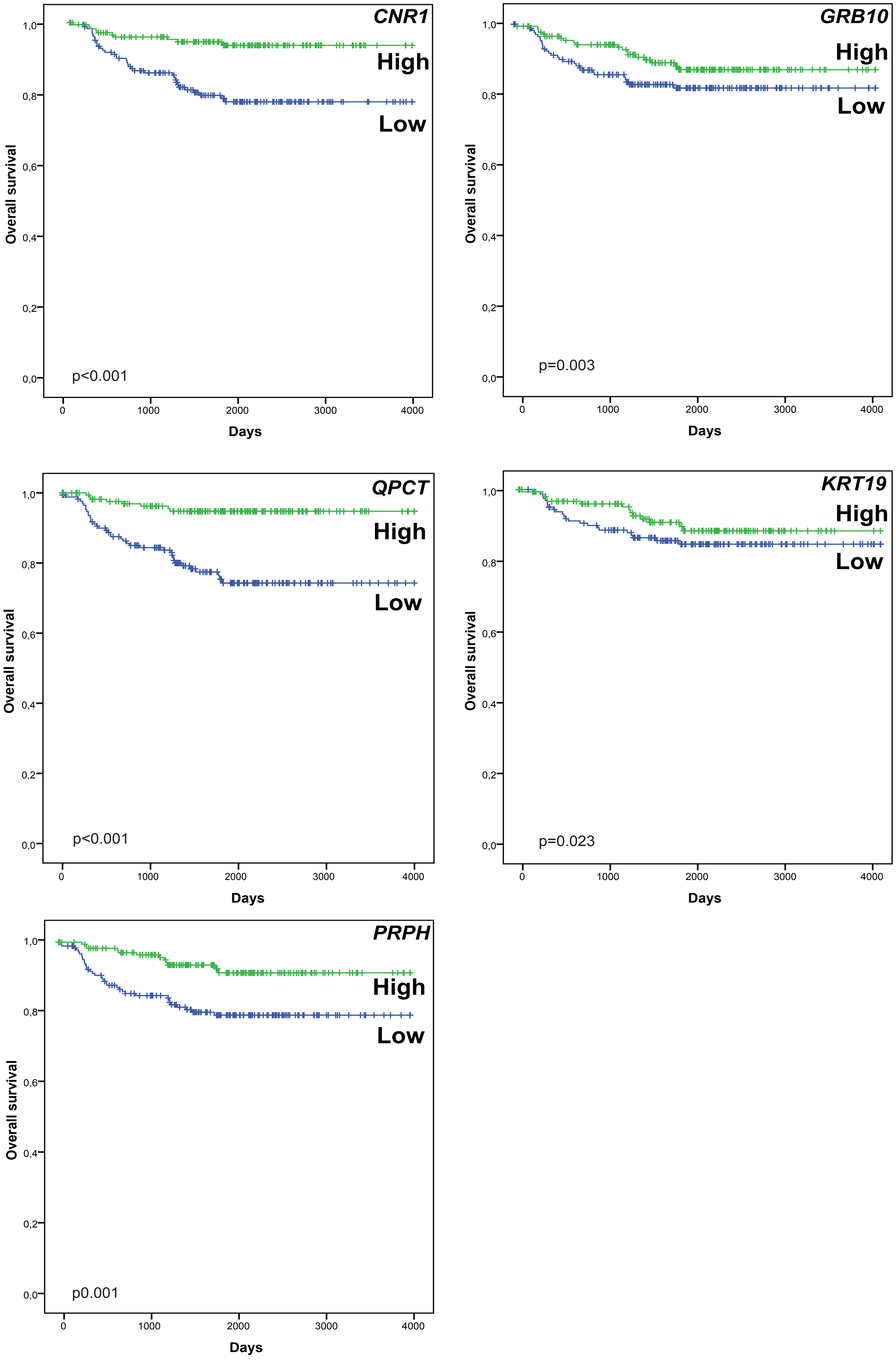
CNR1
High
Low
p<0.001
Overall survival
Days
GRB10
High
Low
p=0.003
QPCT
High
Low
p<0.001
KRT19
High
Low
p=0.023
PRPH
High
Low
p0.001

Survival
