# Supplementary material for: Genome-wide promoter methylation analysis in neuroblastoma identifies prognostic methylation biomarkers
Source: Genome Biol. 2012 Oct 3;13(10):R95. doi: 10.1186/gb-2012-13-10-r95 (PMC3491423; doi:10.1186/gb-2012-13-10-r95)
Supplement: Additional file 5 — Clinical annotation, summary of clinical characteristics, qPCR assays and results of qPCR experiments on 366 NB patient samples (SIOPEN/GPOH cDNA library). Boxplots of the expression levels for CNR1, GRB10, KRT19, PRPH and QPCT in each of the five different NB stages (stages 1, 2, 3, 4 and 4S). A Kaplan-Meier plot shows overall survival according to the relative mRNA expression levels of CNR1, GRB10, KRT19, PRPH and QPCT. [file gb-2012-13-10-r95-S5.pdf]

Part *HIST1H3C* and *ACSS3* qPCR mRNA assay: qPCR mRNA assays used in the mRNA expression profiling of the DNA-methylation biomarkers *HIST1H3C* and *ACSS3*. For each gene, the assay name and corresponding forward and reverse primer (5' to 3') are indicated.

Part *HIST1H3C* – *ACSS3* MSP cell lines: Results of the *HIST1H3C* and *ACSS3* MSP screen on 31 neuroblastoma cell lines. The methylation call (dark blue is methylated, green unmethylated) of each cell line is indicated, as well as the *MYCN* status.

Part *HIST1H3C* qPCR cell lines: Results of the mRNA expression measurement of the DNA-methylation biomarker *HIST1H3C*. The Cq values were converted to relative quantities and converted to log2 values. Relative gene expression levels were then normalized using the geometric mean of the reference sequences *SDHA*, *UBC* and *AluSq*. These logged and normalized qPCR data are given.

**Name**	**Forward primer**	**Reverse primer**
HIST1H3C	AAACAGCTCGCAAGTCTAC	TAGCGATGAGGTTTCTTCAC
ACSS3	GTCATGCTACACTGGTCAAT	AACTGTTGTGTTCCCATGAA

HIST1H3C and *ACSS3* qPCR mRNA assay

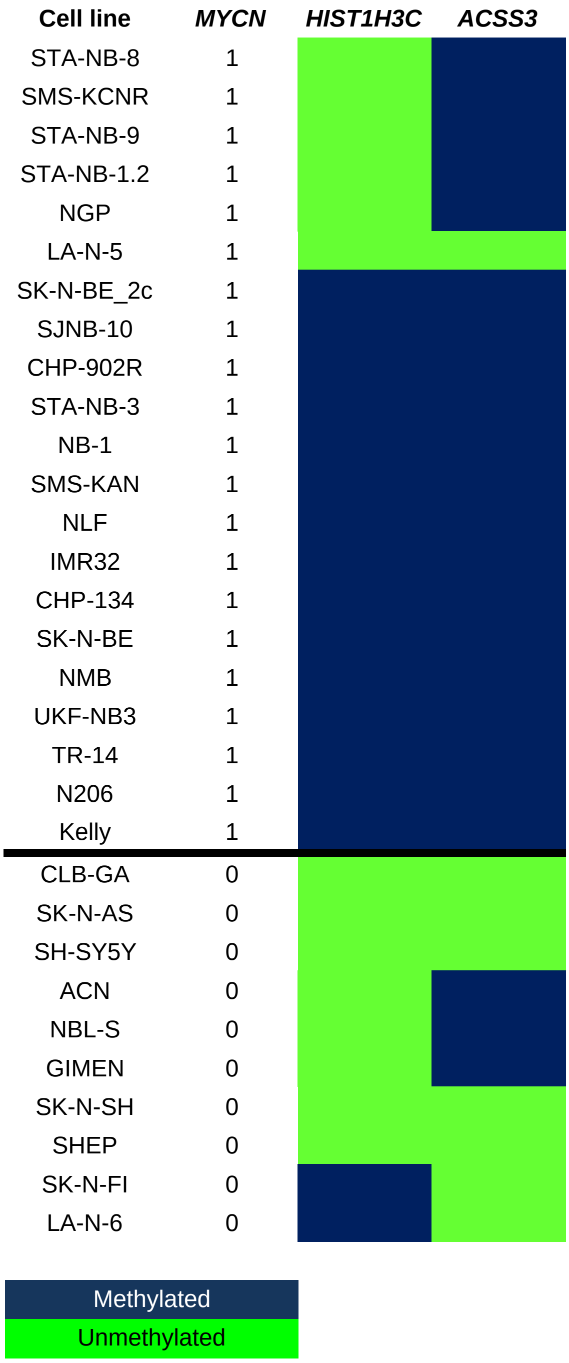

MYCN=1 : *MYCN* amplified
MYCN=0 : *MYCN* single copy

HIST1H3C - ACSS3 MSP cell lines

Cell line	*MYCN* status	*Alu-sq* CNRQ	*Alu-sq* SE(CNRQ)	*SDHA* CNRQ	*SDHA* SE(CNRQ)	*UBC* CNRQ	*UBC* SE(CNRQ)	*HIST1H3C* CNRQ	*HIST1H3C* SE(CNRQ)
SK-N-AS	0	-1,672E-002	1,591E+000	-2,463E-002	1,108E+000	4,135E-002	1,014E+000	1,041E+000	1,330E+000
SJNB-10	1	4,449E-002	4,636E-001	-4,771E-002	5,751E-001	3,221E-003	3,911E+000	-1,264E+000	2,510E-003
NB-1	1	1,077E-001	2,567E+000	-5,825E-002	1,026E+000	-4,946E-002	1,240E+000	8,584E-001	5,764E-001
IMR32	1	1,734E-001	5,734E-001	-5,423E-002	3,672E-001	-1,192E-001	1,387E-001	-1,204E+000	5,426E-003
SKNFI	0	7,861E-002	4,151E-001	-1,114E-001	5,295E-001	3,282E-002	8,039E-001	-1,141E+000	1,790E-002
NLF	1	5,151E-002	3,585E-001	4,812E-002	8,320E-001	-9,963E-002	2,107E-001	-4,591E-001	3,354E-002
NBL-S	0	3,145E-002	1,890E+000	-1,119E-001	1,479E-001	8,049E-002	6,244E-001	1,177E+000	8,499E-001
STA-NB-1.2	1	1,589E-001	2,550E-001	-1,892E-001	2,235E-001	3,031E-002	7,907E-001	1,140E+000	8,258E-001
CLBGA	0	1,941E-002	4,283E-001	7,922E-002	4,496E-002	-9,863E-002	2,067E-002	8,168E-001	5,563E-001
SKNBE(2c)	1	-1,632E-001	4,955E-002	1,254E-001	9,775E-002	3,784E-002	3,014E-001	9,984E-001	7,980E-002
N206	1	-6,137E-002	1,033E-001	4,360E-002	2,519E-001	1,777E-002	4,283E-001	-1,577E+000	1,365E-003
CHP-902R	1	1,539E-002	5,456E-001	-1,280E-001	6,193E-002	1,126E-001	2,575E-001	7,420E-001	1,057E-001
GIMEN	0	-1,075E-001	4,013E-001	-1,052E-003	3,834E+001	1,086E-001	2,767E-001	1,024E+000	2,570E-001
SK-N-SH	0	1,338E-001	2,674E-001	-1,330E-001	1,826E-001	-7,930E-004	8,269E+001	6,542E-001	2,274E-001
SK-N-BE	1	-1,416E-001	6,973E-002	1,108E-001	8,345E-002	3,082E-002	5,712E-001	6,934E-001	1,570E-001
CHP-134	1	-7,442E-002	1,481E-001	1,058E-001	4,231E-001	-3,140E-002	4,359E-001	-2,065E+000	1,000E-003
SMS-KAN	1	4,048E-002	3,663E-001	1,229E-001	3,274E-001	-1,633E-001	4,408E-002	-1,450E+000	4,611E-003
NMB	1	1,343E-003	1,391E+001	1,601E-002	1,515E+000	-1,735E-002	1,811E+000	-1,844E+000	1,913E-004
NGP	1	5,357E-003	1,069E+000	1,099E-002	3,473E-001	-1,635E-002	5,686E-001	1,038E+000	2,869E-001
SH-SY5Y	0	7,710E-002	3,409E-001	1,350E-002	8,436E-001	-9,060E-002	2,215E-001	1,132E+000	8,553E-001
TR-14	1	1,188E-002	1,820E+000	1,274E-001	1,506E-001	-1,393E-001	4,319E-002	-1,033E+000	2,788E-003
LAN-5	1	-1,808E-001	1,372E-001	1,545E-001	4,100E-001	2,630E-002	8,364E-001	1,544E+000	1,587E+000
UKF-NB3	1	-3,674E-003	6,088E+000	1,991E-001	8,749E-002	-1,955E-001	3,945E-002	7,982E-001	6,430E-001
SMS-KCNR	1	5,754E-002	2,699E-001	-7,380E-002	2,602E-001	1,627E-002	8,682E-001	1,177E+000	7,007E-001
STA-NB-3	1	-5,535E-002	1,539E-001	-8,584E-002	5,991E-002	1,412E-001	1,729E-001	-1,690E+000	3,707E-004
SHEP	0	-1,321E-002	4,335E+000	-6,025E-002	3,844E-001	7,346E-002	3,187E-001	4,079E-001	3,260E-001
STA-NB-9	1	-6,589E-002	4,406E-001	-3,918E-002	8,481E-001	1,051E-001	4,568E-001	4,982E-001	1,825E-001
STA-NB-8	1	5,859E-003	3,175E+000	-6,828E-002	6,381E-001	6,242E-002	3,814E-001	4,435E-001	1,474E-001
ACN	0	-1,000E-001	9,114E-002	6,216E-002	5,137E-001	3,784E-002	6,031E-001	1,140E+000	5,969E-001
Kelly	1	2,848E-003	2,507E+000	-5,775E-002	3,371E-001	5,490E-002	1,593E-001	-1,436E+000	4,339E-003
LA-N-6	0	-3,328E-002	3,091E-001	2,504E-002	1,412E+000	8,238E-003	1,436E+000	-2,159E+000	8,909E-005

MYCN=1 : *MYCN* amplified
MYCN=0 : *MYCN* single copy

HIST1H3C qPCR cell lines
